# Effect of imbalance in folate and vitamin B12 in maternal/parental diet on global methylation and regulatory miRNAs

**DOI:** 10.1038/s41598-019-54070-9

**Published:** 2019-11-26

**Authors:** Aatish Mahajan, Divika Sapehia, Shilpa Thakur, Palani Selvam Mohanraj, Rashmi Bagga, Jyotdeep Kaur

**Affiliations:** 1 Department of Biochemistry, Postgraduate Institute of Medical Education and Research, Chandigarh, India; 2 Department of Obstetrics and Gynecology, Postgraduate Institute of Medical Education and Research, Chandigarh, India

**Keywords:** Reverse transcription polymerase chain reaction, miRNAs, Intrauterine growth, DNA methylation, Reverse transcription polymerase chain reaction

## Abstract

DNA methylation, a central component of the epigenetic network is altered in response to nutritional influences. In one-carbon cycle, folate acts as a one-carbon carrier and vitamin B12 acts as co-factor for the enzyme methionine synthase. Both folate and vitamin B12 are the important regulators of DNA methylation which play an important role in development in early life. Previous studies carried out in this regard have shown the individual effects of these vitamins but recently the focus has been to study the combined effects of both the vitamins during pregnancy. Therefore, this study was planned to elucidate the effect of the altered dietary ratio of folate and B12 on the expression of transporters, related miRNAs and DNA methylation in C57BL/6 mice. Female mice were fed diets with 9 combinations of folate and B12 for 4 weeks. They were mated and off-springs born (F1) were continued on the same diet for 6 weeks post-weaning. Maternal and fetal (F2) tissues were collected at day 20 of gestation. Deficient state of folate led to an increase in the expression of folate transporters in both F1 and F2 generations, however, B12 deficiency (BDFN) also led to an increase in the expression in both the generations. B12 transporters/proteins were found to be increased with B12 deficiency in F1 and F2 generations except for TC-II in the kidney which was found to be decreased in the F1 generation. miR-483 was found to be increased with all conditions of folate and B12 in both F1 and F2 generations, however, deficient conditions of B12 led to an increase in the expression of miR-221 in both F1 and F2 generations. The level of miR-133 was found to be increased in BDFN group in F1 generation however; in F2 generation the change in expression was tissue and sex-specific. Global DNA methylation was decreased with deficiency of both folate and B12 in maternal tissues (F1) but increased with folate deficiency in placenta (F1) and under all conditions in fetal tissues (F2). DNA methyltransferases were overall found to be increased with deficiency of folate and B12 in both F1 and F2 generations. Results suggest that the dietary ratio of folate and B12 resulted in altered expression of transporters, miRNAs, and genomic DNA methylation in association with DNMTs.

## Introduction

Folate which is also known as vitamin B9 is derived from dietary sources, acts as a key methyl carrier and is required for the synthesis of nucleotides and methionine^1^. Another member of B vitamin family, vitamin B12 also plays a major role in one-carbon metabolism. When a methyl group is transferred to homocysteine from 5- methyl tetrahydrofolate to form methionine, vitamin B12 acts as co-factor for the enzyme methionine synthase^2^. Folate gets trapped as 5-methyltetrahydrofolate in B12 deficiency which leads to the accumulation of homocysteine and decreased synthesis of methionine^3^.

Only bacteria have the machinery to produce folate and B12 and the requirements of B12 in humans must be met through diet^4^. Being hydrophilic in nature, transporters and binding proteins are there to mediate transfer into cells i.e. proton-coupled folate transporter (PCFT), reduced folate carrier (RFC) and folate receptor (FRα and FRβ) for folate and LMBR1 Domain containing 1 (LMBRD1) and trans-cobalamin II (TC-II) for vitamin B12. Among the folate transporters, RFC has ubiquitous expression while PCFT and FRs have tissue-specific expressions^5,6^. LMBRD1 also known as ‘probable lysosomal cobalamin transporter’ is involved in the conversion of B12 into one of two molecules, methyl cobalamin (MeCbl) or adenosyl cobalamin (AdoCbl) which serve as cofactors for methionine synthase^7^. TC-II encodes for proteins known as R binders and is involved in the transport of cobalamin in the cells^8^.

Folate & vitamin B12 deficiencies, due to dietary factors, are among the most common deficiencies in India^9^ which have been associated with pregnancy-related complications such as NTD. With the mandatory fortification of food with folic acid by USFDA in 1996, the incidence of folate deficiency has become rare^10^. In India, the prevalence of B12 deficiency is high in the reproductive age group due to habits of vegetarian diet and lower social and economic status^9^. People take more than the recommended dose of folic acid as per the practice of prescribing folic acid from periconceptual period throughout the pregnancy and the data from the literature shows that B12 deficiency is masked by high serum levels of folate leading to neurological damage^11^. In this regard, a study conducted in India in Pune has shown that high folate with low B12 levels in pregnant women was associated with the development of insulin resistance in the offspring which suggested that it is the ratio of both vitamins rather than individual levels which is important for normal fetal growth^12^. The incidence of gestational diabetes mellitus (GDM) was also higher with low B12 in a study carried out in Mysore^13^.

Being the key determinants of one carbon metabolism in which S-adenosyl methionine (SAM) is formed, both B12 and folate are essential for DNA methylation and it is a fact that changes in gene expression are caused due to differential DNA methylation patterns that are established in-utero^14,15^. 5 methyl cytosine, a conserved epigenetic mark and associated with gene silencing was found to be high in embryos, which further decreases with age^16,17^. Global DNA methylation decreases with folate depletion whereas increases with folate supplementation^18,19^. In this context, a study carried out in sheep has reported hypomethylation in adult male offspring under maternal folate and B12 dietary restriction which leads to increased adiposity and insulin resistance^20^.

DNA methyltransferases (DNMT) namely DNMT1 maintains methylation patterns during DNA replication, whereas DNMT3A and DNMT3B catalyse de novo DNA methylation^21^. Altered levels of DNMTs can cause hypermethylation and hypo-methylation, which are associated with downregulating and reactivating gene expression^22,23^. Previous studies have shown that folate- and methyl-deficient diet increases the expression of DNMT1, DNMT3A, and DNMT3B in the livers of rats^24^, and folate fortification increases DNMT1 expression in the human cervix^25^. In another study related to folate deficiency, the expression of DNMT1 and DNMT3A were found to be up-regulated in the uteruses of pseudo-pregnant mice^26^. Previously it has been shown that B12 supplementation significantly influenced DNA methylation of genes associated with type 2 diabetes, both alone and when given with folic acid^27^.

The developing embryo obtains all the nutritional requirements from the mother’s diet and various cellular processes depend on in- utero conditions mediated by various genetic as well as epigenetic marks^28^. Therefore, it is quite possible that the dietary ratio of folate and B12 during pregnancy can influence the establishment of DNA methylation marks which can further lead to alterations in various epigenetic mechanisms.

A recent study has shown that B12 supplementation influences the regulation of type 2 diabetes-associated genes by methylation of miR21 which states the epigenetic regulation by alteration of one carbon metabolism^27^. miRNAs are short (19–25 bases) non-coding RNAs, which act as gene transcriptional repressors in animal and plant genomes^29,30^. Several miRNAs have now been identified that are susceptible to regulation by diet. miR-483, located in an intron of the IGF2 gene is one of these. This miRNA controls the ability of adipose tissue to store lipid and has a direct effect on growth differentiation factor 3. Increased expression of miR-483 programmed by nutritional status in early life has been associated with insulin resistance which can lead to type 2 diabetes^31^.

A study has shown that in rats, tumours are induced by methyl deficient diet due to changes in microRNA expression^32^. miR-221 expression was found to be increased in human lymphoblastoid cells under folate deficiency^33,34^. miR-133 is an important regulator of cardiac physiology^35,36^ and its role in cardiomyocytes hypertrophy is well defined which regulates IGF-1 gene expression^35^. In a recent study in mice, it was shown that B12 restriction leads to an increase in the levels of cholesterol and triglycerides^37^. Hence, we planned to study these miRNAs under the experimental setting of altered dietary folate and B12 ratio.

Keeping in view the above findings, the present study was planned to examine the effect of the dietary ratio of folate and B12 on the expression of transporters, global DNA methylation and miRNA associated with micronutrients in maternal and fetal tissues. This study explains the interplay between the nutritional status and epigenetic factors which can have trans-generational effects.

## Results

A Significant interaction of folate and B12 was evident in body weights of mice as analyzed by two-way ANOVA (p < 0.001). The profile plot for interaction is depicted in Supplementary Fig. 1. Pairwise comparison revealed that the % increase in the body weight of mice (F1) was less under the setting of folate and vitamin B12 deficiency i.e. BNFD (39.5%), BDFN (34.5%), BDFD (28.2%) and BDFO (14.7%) groups compared to control (BNFN) animals (66.1%) at the time of mating i.e. after 6 weeks of dietary treatment (Table 1). In BDFO group, most of the animals exhibited a high rate of mortality (5 females (F1) out of 12 were left at the time of mating) and total infertility was observed, therefore no data related to BDFO is present in the manuscript for the F2 generation. There was a significant reduction in pregnancy rate (F1) in BNFD, BDFN and BDFD where only 33%, 41% and 25% of females got pregnant as compared to control (91%).

**Table 1:** Body weight of the females (F1) supplemented with various combination of folate and vitamin B_12_.

Group	% increase in body weight at the time of mating compared to initial body weight(g)
BNFN	66.1%
BNFO	49.5%
BNFD	39.5%^*^
BDFN	34.5%^***^
BDFD	28.2%^***^
BDFO^@^	14.7%^***^
BOFN	52.7%
BOFO	49.9%
BOFD	62.8%

^@^In BDFO group most of the animals died (out of 12 females only 5 left until time of mating). *p < 0.05, **p < 0.01, ***p < 0.001 vs BNFN. The data is presented as mean ± SD. (N = 12).

The sex difference comparison of male and female fetuses (F2) born to pregnant mothers revealed that in BDFN (80% male and 20% female, p < 0.001) and BDFD (66.7% males and 33.3% females, p < 0.01) groups, there was a significant increase in number of males as compared to females in comparison to control, BNFN (50% male and 50% females). In BNFO and BNFD groups, however, 33.3% males and 66.7% females were observed whereas in BOFN and BOFD groups 46.7% males and 53.3% females and in BOFD group 62.5% males and 37.5% females were there in the F2 generation.

Fetuses (F2) born to mothers fed with folate and B12 deficient diet were relatively smaller in size compared to control.

### Effect of diet on serum folate, vitamin B_12_ and homocysteine levels

Folate, vitamin B_12_ and homocysteine levels were estimated in the serum of F1 mothers fed with different dietary combinations of folate and B12 (Table 2).

**Table 2:** Folate, vitamin B_12_ and homocysteine levels in the serum of the animals supplemented with various combination of folate and B_12_.

Diet Group	Folate levels (ng/ml)	Vitamin B12 levels (pg/ml)	Homocysteine levels (μmol/L)
BNFN	33.34 ± 5.87	427.4 ± 4.76	10.56 ± 0.36
BNFO	57.74 ± 5.39^***^	418.92 ± 9.16	8.77 ± 0.55^*^
BNFD	6.01 ± 1.36^***^	429.42 ± 6.77	18.96 ± 1.58^***^
BDFN	49.22 ± 3.66^**^	190 ± 9.00^***^	8.66 ± 0.45^*^
BDFD	5.79 ± 0.98^***###^	169.93 ± 2.86^***#$$$^	15.78 ± 0.18^***###$$$^
BDFO	75.91 ± 6.19^***###&&^	144.38 ± 9.14^***###&&&^	7.81 ± 0.69^**^
BOFN	51.34 ± 3.33**	524.22 ± 2.87^***^	8.67 ± 0.72^*^
BOFO	73.47 ± 8.22^***^^^&&^	511.69 ± 2.40^***&&&^	10.66 ± 0.56^^&^
BOFD	7.70 ± 1.04^***^^^^	521.75 ± 5.93^***$$$^	11.66 ± 0.60^^^^$$$^

Values are expressed as mean ± SD; n = 5.

Analysis of folate, B12 and homocysteine levels in mother by two way ANOVA revealed that for folate levels there was a significant interaction between folate and B12 (p < 0.01), for B12 and homocysteine, the interaction was also found to be significant (p < 0.001). The profile plots for interaction between folate and B12 are depicted in Supplementary Fig. 2.

The pairwise comparison revealed a significant decrease in serum folate levels in BNFD, BDFD and BOFD groups by 81.9%, 82.6% and 76.9% as compared to the control group (BNFN). Serum folate levels were significantly increased in BNFO, BDFO and BOFO groups by 73.1%, 127.6% and 120.3% respectively as compared to animals fed with normal folate/normal B12 diet.

Similarly, serum B_12_ levels were significantly decreased in animals given vitamin B_12_ deficient diet viz. BDFN, BDFD and BDFO by 55.54%, 60.24% and 66.2% respectively. Moreover, serum B_12_ levels were observed to be significantly increased in animals over supplemented with vitamin B_12_. The results demonstrated that the levels of folate and vitamin B_12_ were decreased in deficient conditions and increased in over-supplemented conditions of respective vitamins.

A significant increase in homocysteine (Hcy) levels was observed in serum of the animals fed folate deficient diet. The high levels of Hcy were observed in BNFD and BDFD groups by 79.5% and 49.4%respectively as compared to control animals. However, a slight but non-significant increase in Hcy levels was observed in the BOFD group of animals. As expected, folate and B_12_ over-supplementation significantly decreased the serum Hcy levels in the animals.

### Effect of diet on expression of folate and B12 transporters

#### mRNA expression of RFC

Maternal tissues (F1). Analysis of the RFC gene in mother by two way ANOVA revealed that there was a significant interaction between folate and B12 in the brain (p < 0.01), kidney (p < 0.001), liver (p < 0.001) and placenta (p < 0.001). The profile plots for interaction between folate and B12 for maternal as well as fetal tissues are depicted in Supplementary Fig. 3.

Analyzing the expression of RFC by pairwise comparisons, in comparison to BNFN with all conditions of B12 deficiency (BNFN vs BDFN, BDFD, BDFO), B12 deficiency irrespective of folate status led to an increase in the expression. B12 over-supplementation state combined with folate deficiency (BOFD) led to an increase in expression in all tissues however, expression was also found to be increased in the liver in BOFN and BOFO groups as compared to BNFN.

**Figure 1:**
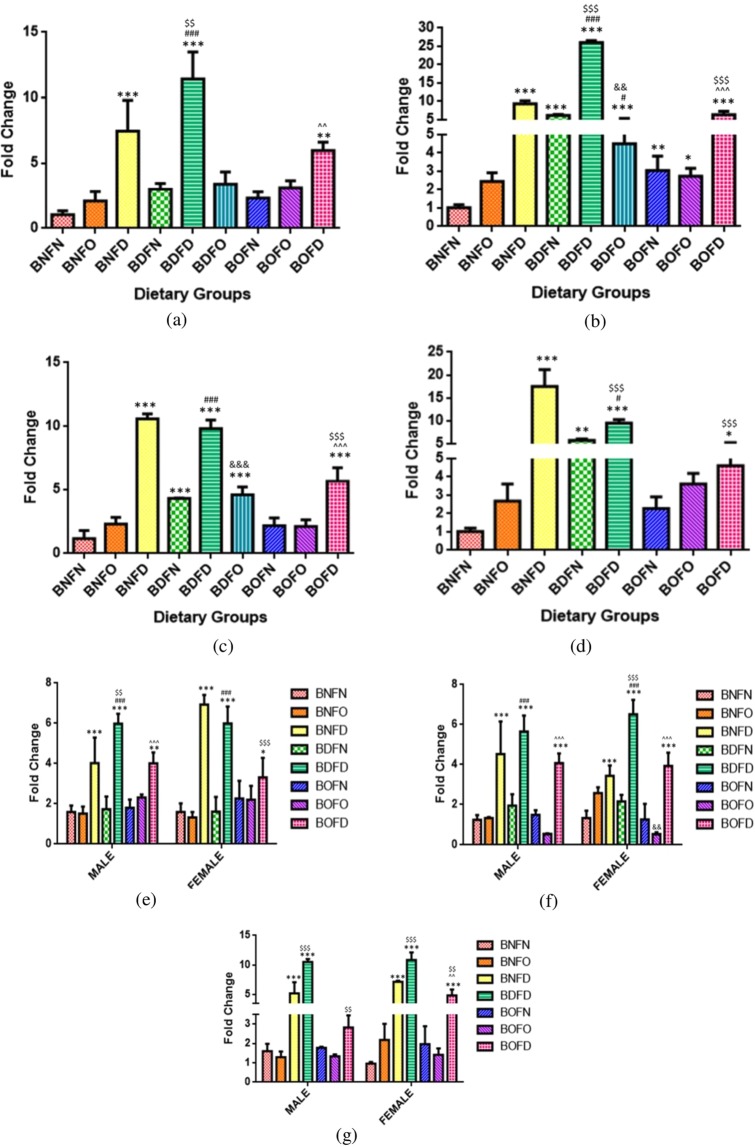
Fold change in mRNA of RFC gene in maternal tissues. (**a**) Brain (F = 31.03,df = 8)). (**b**) Liver (F = 573.3, df = 8). (**c**) Kidney (F = 119.1,df = 8)). (**d**) Placenta (F = 57.45, df = 7), fetal tissues. (**e**) Liver (Male) (F = 28.6, df = 7), (Fe-male) (F = 37.3, df = 7). (**f**) Brain (Male) (F = 28.4, df = 7), (Fe-male) (F = 53.3, df = 7). (**g**) Kidney (Male) (F = 70.7, df = 6), (Fe-male) (F = 53.7, df = 6) normalized with GAPDH. *p < 0.05, **p < 0.01, ***p < 0.001 vs BNFN, ^#^p < 0.05, ^##^p < 0.01, ^###^p < 0.001 vs BDFN, ^p < 0.05, ^^p < 0.01, ^^^p < 0.001 vs BOFN, ^$^p < 0.05, ^$$^p < 0.01, ^$$$^p < 0.001 vs BNFD and ^&^p < 0.05, ^&&^p < 0.01, ^&&&^p < 0.001 vs BNFO. The data is presented as mean ± SD^.^ (N = 4). B12 normal folate normal (BNFN), B12 normal folate over-supplemented ^(^BNFO), B12 normal folate deficient (BNFD), B12 deficient folate normal (BDFN), B12 deficient folate over-supplemented (BDFO), B12 deficient folate deficient (BDFD), B12 over-supplemented folate normal (BOFN), B12 over-supplemented folate over-supplemented (BOFO), B12 over-supplemented folate deficient (BOFD).

On comparing the effect of folate deficiency with either state of B12 in comparison to control, an increase in expression of the RFC gene in all tissues was observed (BNFN vs BNFD, BDFD, BOFD). Folate over-supplementation in combination with B12 normal (BNFO) did not affect the expression however in combination with B12 over-supplementation (BOFO) led to an increase in expression in the liver. (Fig. 1a–d).

Fetal tissues (F2). Analysis of RFC gene in fetal tissues by two way ANOVA revealed that there was a significant interaction between folate and B12 in the brain (female) (p < 0.001), kidney (male) (p < 0.01), kidney (female) (p < 0.01), liver (male) (p < 0.01) and liver (female) (p < 0.001) however in brain (male) (p = 0.29) interaction was not found to be significant.

In the brain (male) on analyzing the independent effects of B12, it was observed that in B12 deficient (BD) group, the expression of RFC was significantly increased whereas, with B12 over-supplementation (BO) expression was not changed as compared to B12 normal (BN). Analyzing the independent effects of folate revealed that the expression was found to be increased in folate- deficient group (FD) and no change was observed with folate over-supplementation (FO) when compared to folate normal (FN).

Analyzing the expression of RFC by pairwise comparisons, in comparison to BNFN with all the conditions of B12 deficiency, combination with folate deficiency (BDFD) led to an increase in expression in the fetus whereas mRNA levels were not changed in combination to normal folate (BDFN) in all fetal tissues. The State of B12 over- supplementation when combined to folate deficiency (BOFD) led to an increase in the expression whereas no change was observed with BOFN and BOFO in fetuses as compared to BNFN.

On comparing the effect of folate deficiency with either state of B12 in comparison to control, an increase in the expression of RFC was observed regardless of the sex of in all the fetal tissues (BNFN vs BNFD, BDFD, BOFD). Folate over-supplementation when combined to B12 normal (BNFO) and B12 over-supplementation (BOFO) had no effect on the expression of RFC as compared to BNFN in all fetal tissues (Fig. 1e–g).

#### mRNA expression of PCFT

Maternal tissues (F1). Analysis of PCFT gene in mother by two-way ANOVA revealed that there was a significant interaction between folate and B12 in the kidney (p < 0.001), liver (p < 0.001) and placenta (p < 0.001) except brain (p = 0.06) where it was not significant. The profile plots for the interaction of folate and B12 in maternal as well as fetal tissues are depicted in Supplementary Fig. 4.

In the brain on analyzing the independent effects of B12, it was observed that in B12 deficient (BD) groups, the expression of PCFT was significantly increased however no change was observed with B12 over-supplementation (BO) as compared to B12 normal (BN). Analyzing the independent effects of folate also revealed that the expression was found to be increased in folate deficient group (FD) in the brain however no change was observed with folate over-supplementation (FO) when compared to folate normal (FN).

Analyzing the expression of PCFT by pairwise comparisons, in comparison to BNFN with all the conditions of B12 deficiency (BNFN vs BDFN, BDFD, BDFO), combination with folate normal as well as folate deficiency (BDFN, BDFD) led to an increase in expression in all maternal tissues whereas that with folate over-supplementation (BDFO) increased the expression only in the liver. B12 over-supplementation, in combination with either normal folate or folate over-supplementation (BOFN, BOFO), had no effect but when combined to folate deficiency (BOFD) increased the PCFT transcript as compared to BNFN in all tissues.

**Figure 2:**
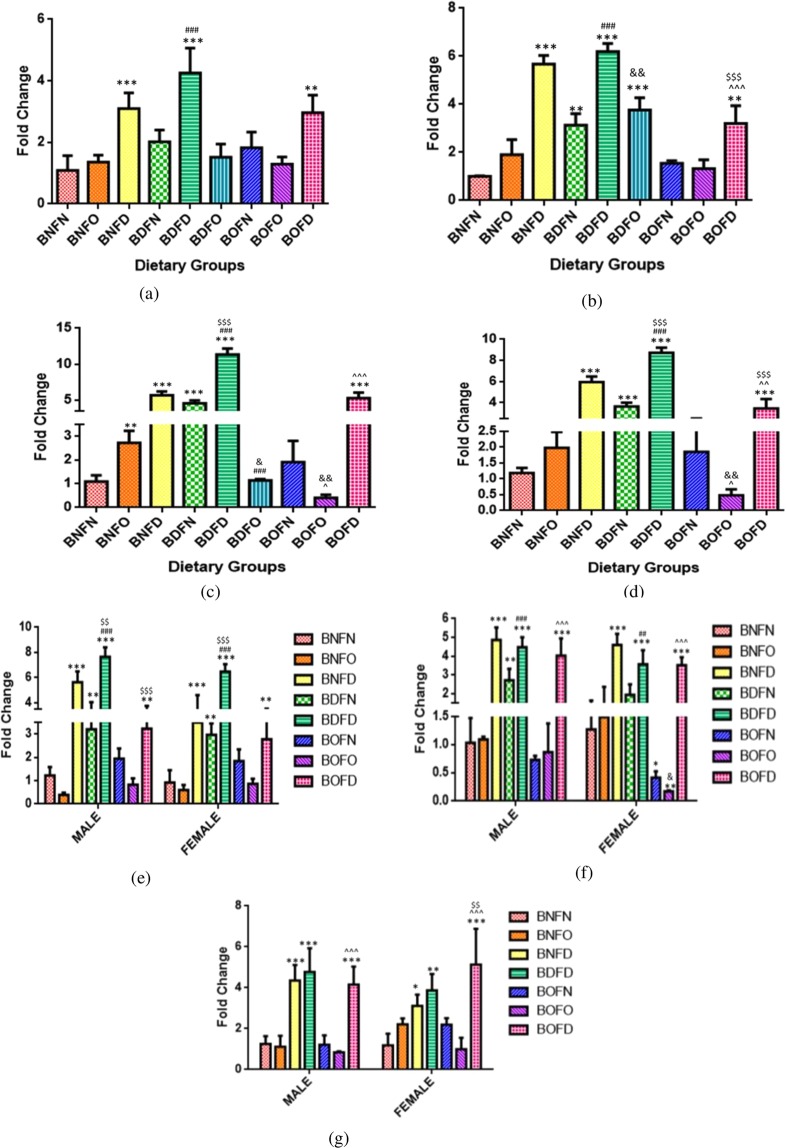
Fold change in mRNA of PCFT gene in maternal tissues. (**a**) Brain (F = 18.62, df = 8). (**b**) Liver (F = 68.35, df = 8). **(c**) Kidney (F = 144.7, df = 8). (**d**) Placenta (F = 110.3, df = 7), fetal tissues. (**e**) Liver (Male) (F = 25.4, df = 7), (Female) (F = 39.8, df = 7). (**f**) Brain (Male) (F = 41.3, df = 7), (Female) (F = 35.5, df = 7). (**g**) Kidney (Male) (F = 26.4, df = 6), (Female) (F = 12.8, df = 6) normalized with GAPDH. *p < 0.05, **p < 0.01, ***p < 0.001 vs BNFN, ^#^p < 0.05, ^##^p < 0.01, ^###^p < 0.001 vs BDFN, ^p < 0.05, ^^p < 0.01, ^^^p < 0.001 vs BOFN, ^$^p < 0.05, ^$$^p < 0.01, ^$$$^p < 0.001 vs BNFD and ^&^p < 0.05, ^&&^p < 0.01, ^&&&^p < 0.001 vs BNFO. The data is presented as mean ± SD^.^ (N = 4). B12 normal folate normal (BNFN), B12 normal folate over-supplemented (BNFO), B12 normal folate deficient (BNFD), B12 deficient folate normal (BDFN), B12 deficient folate over-supplemented (BDFO), B12 deficient folate deficient (BDFD), B12 over-supplemented folate normal (BOFN), B12 over-supplemented folate over-supplemented (BOFO), B12 over-supplemented folate deficient (BOFD).

On comparing the effect of folate deficiency with either state of B12 in comparison to control, an increase in expression of PCFT gene in all maternal tissues was seen (BNFN vs BNFD, BDFD, BOFD). Folate over-supplementation combined with B12 deficiency (BDFO) increased the expression in the liver whereas combination with B12 normal (BNFO) increased the mRNA levels of PCFT in the kidney as compared to BNFN. (Fig. 2a–d).

Fetal tissues (F2). Analysis of PCFT gene in fetal tissues by two way ANOVA revealed that there was a significant interaction between folate and B12 in the brain (male) (p < 0.01), brain (female) (p < 0.01), kidney (female) (p < 0.01), liver (male) (p < 0.001) and liver (female) (p < 0.01) except kidney (male) (p = 0.94) where interaction was not significant.

In the kidney (male) on analyzing the independent effects of B12, it was observed that in B12 deficient (BD) group the expression of PCFT was significantly increased whereas with B12 over-supplementation (BO) expression was not changed as compared to B12 normal (BN). Analyzing the independent effects of folate revealed that mRNA levels of PCFT were increased in folate deficient group (FD) in the kidney (male) however no change was observed with folate over-supplementation (FO) when compared to folate normal (FN).

Analyzing the expression of PCFT by pairwise comparisons, in comparison to BNFN with all the conditions of B12 deficiency, combination with folate deficiency (BDFD) resulted in an increase in expression in all fetal tissues regardless of sex whereas B12 deficiency when combined to folate normal (BDFN) led to an increase in expression in the liver in fetuses of both sex along with increase in brain (male). However, under the state of B12 over supplementation, an increase in transcript levels of PCFT was observed in the fetuses in combination with folate deficiency (BOFD) regardless of sex whereas in the brain (female) expression was decreased in BOFN and BOFO groups.

On comparing the effect of folate deficiency with either condition of B12 in comparison to control, an increase in expression of PCFT gene was observed regardless of sex in all fetal tissues (BNFN) vs BNFD, BDFD, BOFD). Folate over-supplementation) in combination with BNFO and BOFO did not affect n the expression of PCFT as compared to BNFN in all fetal tissues except the brain (female) where expression was reduced in BOFO group (Fig. 2e–g).

#### mRNA expression of FOLR1

Maternal tissues (F1). Analysis of FOLR1 gene in mothers by two way ANOVA revealed that there was a significant interaction between folate and B12 in the brain (p < 0.001), kidney (p < 0.001), liver (p < 0.001) and placenta (p < 0.001). The profile plots for the interaction of folate and B12 in maternal as well as fetal tissues are depicted in Supplementary Fig. 5.

Quantitation of FOLR1 transcript by pairwise comparisons revealed that in comparison to BNFN, condition of B12 deficiency in combination with folate deficiency (BDFD) and folate normal (BDFN) resulted in an increased transcript in all the tissues whereas in combination with folate over-supplementation (BDFO) it was increased in the brain and kidney (BNFN vs BDFN, BDFD, BDFO). Under the conditions of B12 over-supplementation, combination with either normal or deficient (BOFN and BOFD) led to an increase in expression of FOLR1 in the liver, kidney and placenta as compared to BNFN.

**Figure 3:**
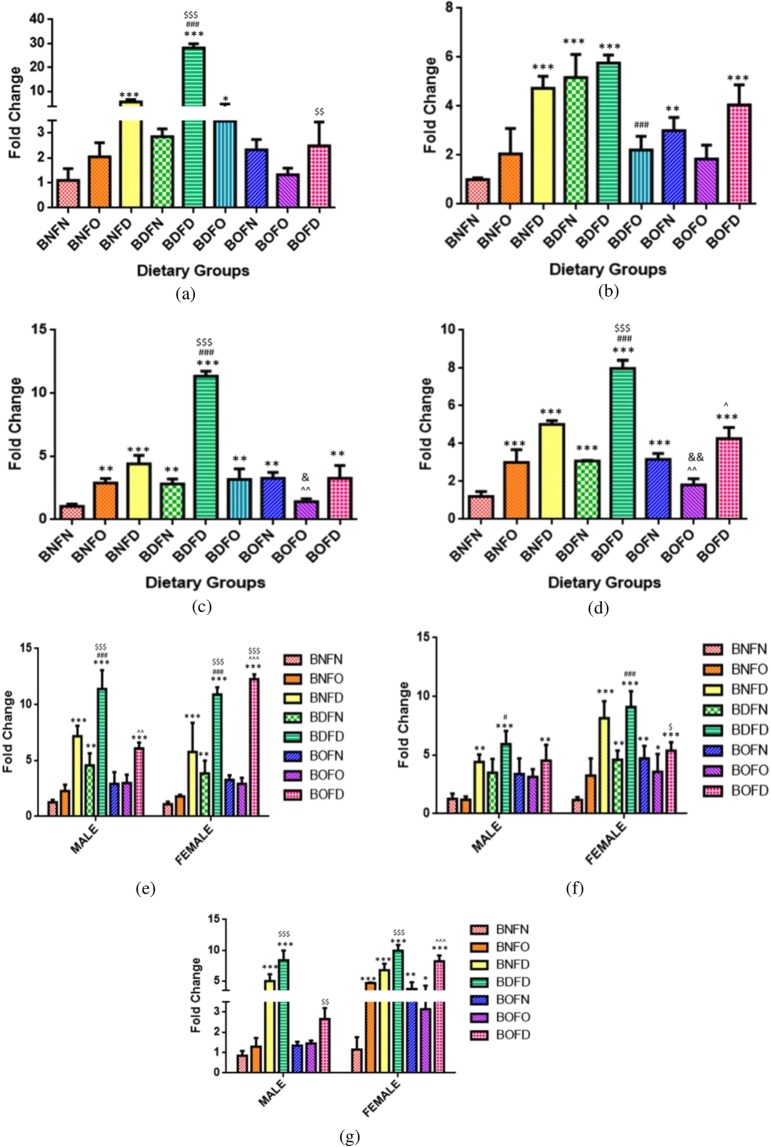
Fold change in mRNA of FOLR1 gene in maternal tissues. (**a**) Brain (F = 364.0, df = 8). (**b**) Liver (F = 25.91, df = 8). (**c**) Kidney (F = 113.6, df = 8). (**d**) Placenta (F = 112.6, df = 7), fetal tissues. (**e**) Liver (Male) (F = 48.5, df = 7), (Female) (F = 60.8, df = 7). (**f**) Brain (Male) (F = 11.2, df = 7), (Female) (F = 19.9, df = 7). (**g**) Kidney (Male) (F = 50.1, df = 6), (Female) (F = 46.5, df = 6) normalized with GAPDH. *p < 0.05, **p < 0.01, ***p < 0.001 vs BNFN, ^#^p < 0.05, ^##^p < 0.01, ^###^p < 0.001 vs BDFN, ^p < 0.05, ^^p < 0.01, ^^^p < 0.001 vs BOFN, ^$^p < 0.05, ^$$^p < 0.01, ^$$$^p < 0.001 vs BNFD and ^&^p < 0.05, ^&&^p < 0.01, ^&&&^p < 0.001 vs BNFO. The data is presented as mean ± SD^.^ (N = 4). B12 normal folate normal (BNFN), B12 normal folate over-supplemented (BNFO), B12 normal folate deficient (BNFD), B12 deficient folate normal (BDFN), B12 deficient folate over-supplemented (BDFO), B12 deficient folate deficient (BDFD), B12 over-supplemented folate normal (BOFN), B12 over-supplemented folate over-supplemented (BOFO), B12 over-supplemented folate deficient (BOFD).

On comparing the effect of folate-deficiency with either state of B12 in comparison to control, an increase in expression of FOLR1 gene was observed (BNFN vs BNFD, BDFD, BOFD). However, the state of folate over-supplementation in combination with B12 deficiency (BDFO) led to an increase in FOLR1 expression in the brain and kidney whereas, in combination with B12 normal (BNFO), it was increased in the kidney and placenta in comparison to BNFN. (Fig. 3a–d).

Fetal tissues (F2). Analysis of FOLR1 gene in fetal tissues by two way ANOVA revealed that there was a significant interaction between folate and B12 in the brain (male) (p < 0.05), brain (female) (p < 0.001), kidney (male) (p < 0.01), kidney (female) (p < 0.001), liver (male) (p < 0.01) and liver (female) (p < 0.001).

Pairwise comparisons of BNFN with the condition of B12 deficiency in combination with either state of folate revealed that combination with folate deficiency (BDFD) led to an increase in mRNA levels of FOLR1 in all fetal tissues regardless of sex whereas, in combination with normal folate l (BDFN), the expression was increased in the liver (male and female) along with the brain (female). Under the conditions of B12 over-supplementation, combination with folate deficiency (BOFD) increased the expression in fetal tissues whereas that with folate normal and over-supplementation (BOFN and BOFO) increased FOLR1 in the brain and kidney (female) in comparison to BNFN.

Analyzing the effect of folate deficiency with either state of B12 in comparison to control revealed an increase in the expression of FOLR1 gene in all fetal tissues regardless of sex (BNFN vs BNFD, BDFD, BOFD). In the case of folate over-supplementation, combination with B12 over- supplementation (BOFO) increased the expression in the brain and kidney (female) as compared to BNFN. (Fig. 3e–g).

#### mRNA expression of LMBRD1

Maternal tissues (F1). LMBRD1 gene expression analysis in mother by two way ANOVA revealed that there was a significant interaction between folate and B12 in the brain (p < 0.001), kidney (p < 0.001), liver (p < 0.001) and placenta (p < 0.001). The profile plots for the interaction of folate and B12 in maternal as well as fetal tissues are depicted in Supplementary Fig. 6.

Further, in comparison to BNFN, B12 deficiency in combination to folate deficiency (BDFD) led to an increase in expression in the liver and placenta, while B12 deficiency in combination with folate over-supplementation (BDFO) resulted in an increase in expression in the brain and liver. However, B12 deficiency with normal folate (BDFN) led to a decrease in expression in the brain. Under the condition of B12 over-supplementation (BNFN vs BOFN, BOFO), expression was reduced in combination with folate over-supplementation (BOFO) in the brain and kidney whereas it was increased in the brain and placenta in BOFN group as compared to BNFN.

**Figure 4:**
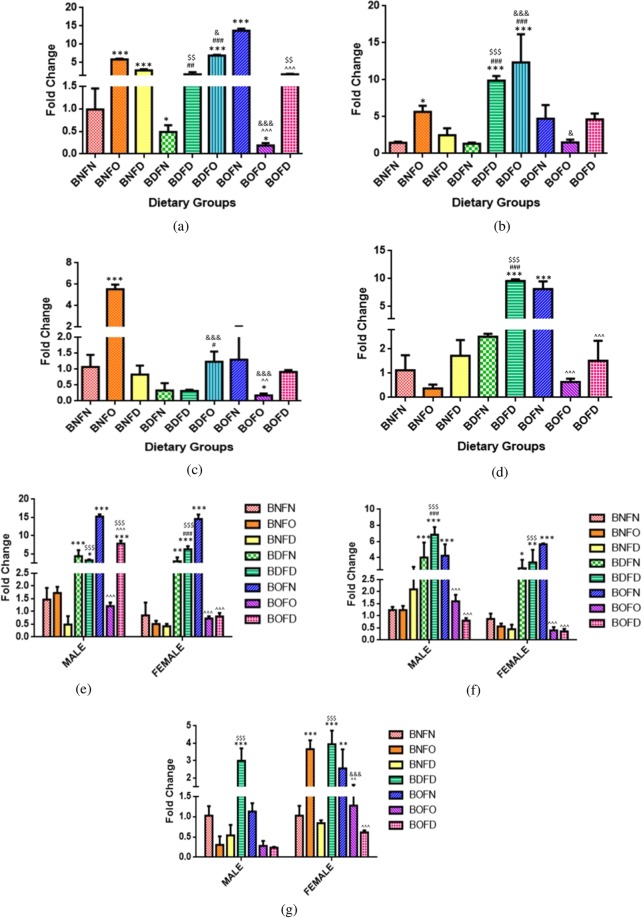
Fold change in mRNA of LMBRD1 gene in maternal tissues. (**a**) Brain (F = 619.9, df = 8). (**b**) Liver (F = 26.20, df = 8) (**c**) Kidney (F = 85.36, df = 8). **(d**) Placenta (F = 114.6, df = 7), fetal tissues. (**e**) Liver (Male) (F = 180.2, df = 7), (Female) (F = 207.2, df = 7). (**f**) Brain (Male) (F = 19.7, df = 7), (Female) (F = 32.6, df = 7). (**g**) Kidney (Male) (F = 36.2, df = 6), (Female) (F = 24.0, df = 6) normalized with GAPDH. *p < 0.05, **p < 0.01, ***p < 0.001 vs BNFN, ^#^p < 0.05, ^##^p < 0.01, ^###^p < 0.001 vs BDFN, ^p < 0.05, ^^p < 0.01, ^^^p < 0.001 vs BOFN, ^$^p < 0.05, ^$$^p < 0.01, ^$$$^p < 0.001 vs BNFD and ^&^p < 0.05, ^&&^p < 0.01, ^&&&^p < 0.001 vs BNFO. The data is presented as mean ± SD. (N = 4). B12 normal folate normal (BNFN), B12 normal folate over-supplemented (BNFO), B12 normal folate deficient (BNFD), B12 deficient folate normal (BDFN), B12 deficient folate over-supplemented (BDFO), B12 deficient folate deficient (BDFD), B12 over-supplemented folate normal (BOFN), B12 over-supplemented folate over-supplemented (BOFO), B12 over-supplemented folate deficient (BOFD).

Comparing the effect of folate deficiency with either state of B12 in comparison to control, combination with B12 deficiency (BDFD) increased the LMBRD1 transcript in the liver and placenta whereas it was increased in the brain in BNFD group (BNFN vs BNFD, BDFD). Under the conditions of folate over-supplementation, mRNA expression of the B12 transporter was increased in the brain, liver and kidney in combination with B12 normal (BNFO), brain and liver in combination with B12 deficiency (BDFO) and decreased in the brain and kidney in BOFO in comparison to BNFN (Fig. 4a–d).

Fetal tissues (F2). LMBRD1 gene analysis in fetal tissues by two way ANOVA revealed that there was a significant interaction between folate and B12 in the brain (male) (p < 0.001), brain (female) (p < 0.001), kidney (female) (p < 0.001), liver (male) (p < 0.001) and liver (female) (p < 0.001) except kidney (male) (p = 0.45) where interaction was not significant.

On analyzing the independent effects of B12 in the case of the kidney (male), it was found that in B12 deficient group (BD) the expression of LMBRD1 was significantly increased whereas, with B12 over-supplementation (BO), expression was not changed as compared to B12 normal (BN). However, with folate, it was found that expression in kidney (male) was not changed with folate-deficient group (FD) whereas folate over-supplementation (FO) led to a decrease in the expression when compared to folate normal (FN).

B12 deficiency with either state of folate in comparison to control (BNFN) revealed that combination with folate normal (BDFN) as well as folate deficiency (BDFD) led to an increase in transcript levels of LMBRD1 in all fetal tissues. Under the condition of B12 over-supplementation, the LMBRD1 mRNA levels were increased in combination with folate normal (BOFN) in all fetal tissues regardless of sex whereas, in combination with folate deficiency (BOFD), the expression was increased in the liver (male) as compared to BNFN.

Condition of folate deficiency in combination with B12 deficiency (BDFD) resulted in a raised level of LMBRD1 gene all fetal tissues regardless of sex whereas combination with B12 over-supplementation (BOFD) showed such effects in the liver (male) only as compared to BNFN. Condition of folate over-supplementation combined with either state of B12 had no significant effect on the expression in comparison to BNFN (Fig. 4e–g).

#### mRNA expression of TC-II

Maternal tissues (F1). Interaction of folate and B12 in the TC-II gene was found to be significant in the brain (p < 0.05), kidney (p < 0.001) and liver (p < 0.001) except placenta (p = 0.19). The profile plots for the interaction of folate and B12 in maternal as well as fetal tissues are depicted in Supplementary Fig. 7.

In the placenta, B12 deficiency (BD) independently led to an increase in the levels of transcripts of TC-II whereas B12 over-supplementation (BO) did not affect the expression of TC-II as compared to B12 normal (BN). Folate deficiency (FD) had no significant effect on mRNA expression of TC-II in the placenta, however with folate over-supplementation (FO) expression was decreased in the placenta when compared to folate normal (FN).

B12 deficiency in combination to either condition of folate compared to control (BNFN), revealed an increase in the expression of TC-II in combination with folate normal (BDFN) and folate deficiency (BDFD) in the liver and placenta whereas expression was found to be decreased in kidney, however, in BDFO group increase in mRNA levels of TC-II was observed in brain and decreased in kidney. Under the conditions of B12 over-supplementation, expression was reduced in the kidney in combination with folate normal (BOFN) and folate deficiency (BOFD) as compared to BNFN.

**Figure 5:**
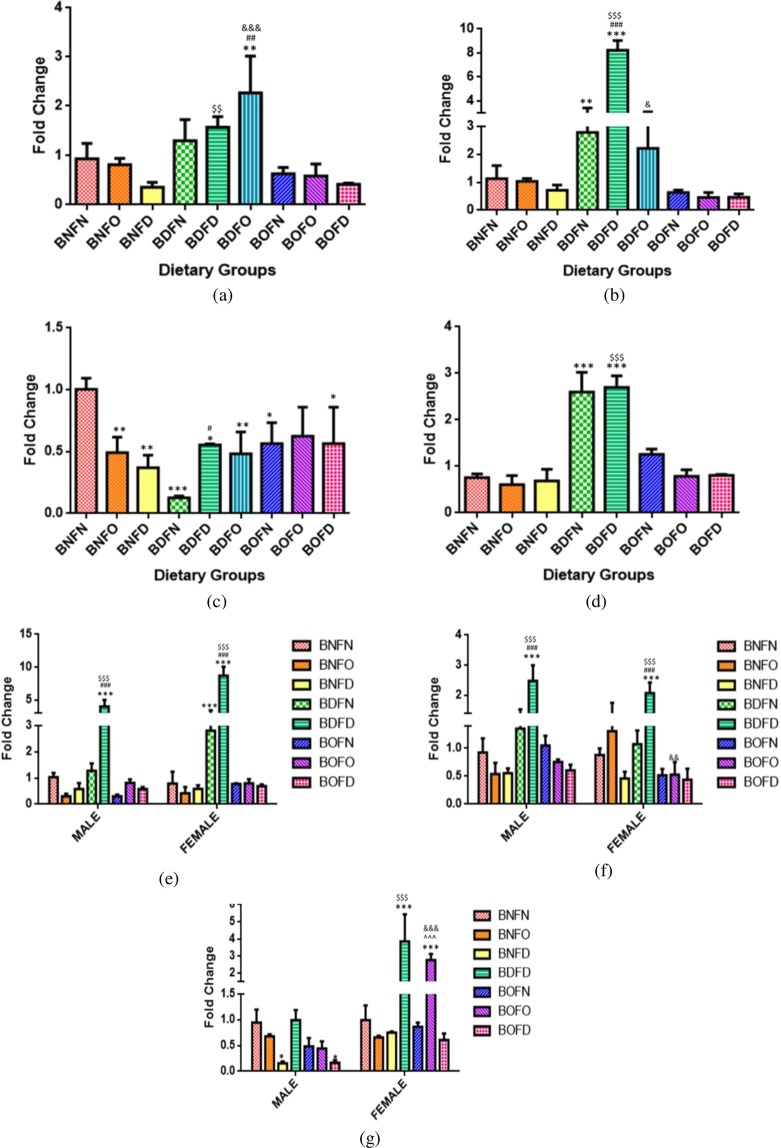
Fold change in mRNA of TC-II gene in maternal tissues. (**a**) Brain (F = 14.25, df = 8). (**b**) Liver (F = 104.1, df = 8). (**c**) Kidney (F = 8.166, df = 8). **(d**) Placenta (F = 47.26, df = 7), fetal tissues. (**e**) Liver (Male) (F = 40.3, df = 7), (Fe-male) (F = 100.2, df = 7). (**f**) Brain (Male) (F = 29.9, df = 7), (Fe-male) (F = 20.5, df = 7). (**g**) Kidney (Male) (F = 20.8, df = 6), (Fe-male) (F = 16.7, df = 6) normalized with GAPDH. *p < 0.05, **p < 0.01, ***p < 0.001 vs BNFN, ^#^p < 0.05, ^##^p < 0.01, ^###^p < 0.001 vs BDFN, ^p < 0.05, ^^p < 0.01, ^^^p < 0.001 vs BOFN, ^$^p < 0.05, ^$$^p < 0.01, ^$$$^p < 0.001 vs BNFD and ^&^p < 0.05, ^&&^p < 0.01, ^&&&^p < 0.001 vs BNFO. The data is presented as mean ± SD. (N = 4). B12 normal folate normal (BNFN), B12 normal folate over-supplemented (BNFO), B12 normal folate deficient (BNFD), B12 deficient folate normal (BDFN), B12 deficient folate over-supplemented (BDFO), B12 deficient folate deficient (BDFD), B12 over-supplemented folate normal (BOFN), B12 over-supplemented folate over-supplemented (BOFO), B12 over-supplemented folate deficient (BOFD).

Folate deficiency combined to either state of B12 revealed a decrease in mRNA of TC-II in the kidney, however, combination with B12 deficiency (BDFD) led to an increase in expression in liver and placenta as compared to BNFN (BNFN vs BNFD, BDFD, BOFD). Condition of folate over-supplementation combined to B12 normal (BNFO) as well as B12 deficiency (BDFO) led to a decrease in the expression in the kidney as compared to BNFN. (Fig. 5a–d).

Fetal tissues (F2). Interaction of folate and B12 in TC-II gene was found to be significant in the brain (male) (p < 0.001), brain (female) (p < 0.001), kidney (male) (p < 0.05), kidney (female) (p < 0.01), liver (male) (p < 0.001) and liver (female) (p < 0.001).

Compared to BNFN, B12 deficiency in combination with folate deficiency (BDFD) resulted in an increase in the expression of TC-II in all fetal tissues regardless of sex whereas no change was observed in combination with normal folate (BDFN). Under the conditions of B12 over-supplementation, TC-II transcript levels were decreased in the kidney (male) in combination with folate deficiency (BOFD) whereas an increase in expression was evident in the kidney (female) in BOFO group as compared to BNFN.

Condition of folate deficiency in combination with B12 deficiency (BDFD) resulted in an increase in the expression of TC-II in all fetal tissues whereas in the kidney (male) mRNA expression was found to be reduced in combination to B12 normal (BNFD) and B12 over-supplementation (BOFD) in comparison to BNFN. Folate over-supplementation resulted in no significant change in the expression of TC-II when combined with either condition of B12 (Fig. 5e–g).

### Effect of diet on expression of microRNAs

#### Expression miR-483

Maternal tissues (F1). A significant interaction of folate and B12 was found in the brain (p < 0.001) and placenta (p < 0.001). The profile plots for the interaction of folate and B12 in maternal as well as fetal tissues are depicted in Supplementary Fig. 8.

B12 deficiency in combination with folate normal (BDFN) and folate deficiency (BDFD) increased the expression of miR-483 in all maternal tissues when compared to control (BNFN). B12 over-supplementation in combination with folate normal (BOFN) as well as folate over-supplementation (BOFO) led to an increase in expression of miR-483 in the brain and placenta, however folate deficiency (BOFD) decreased the miR-483 levels in the placenta.

**Figure 6:**
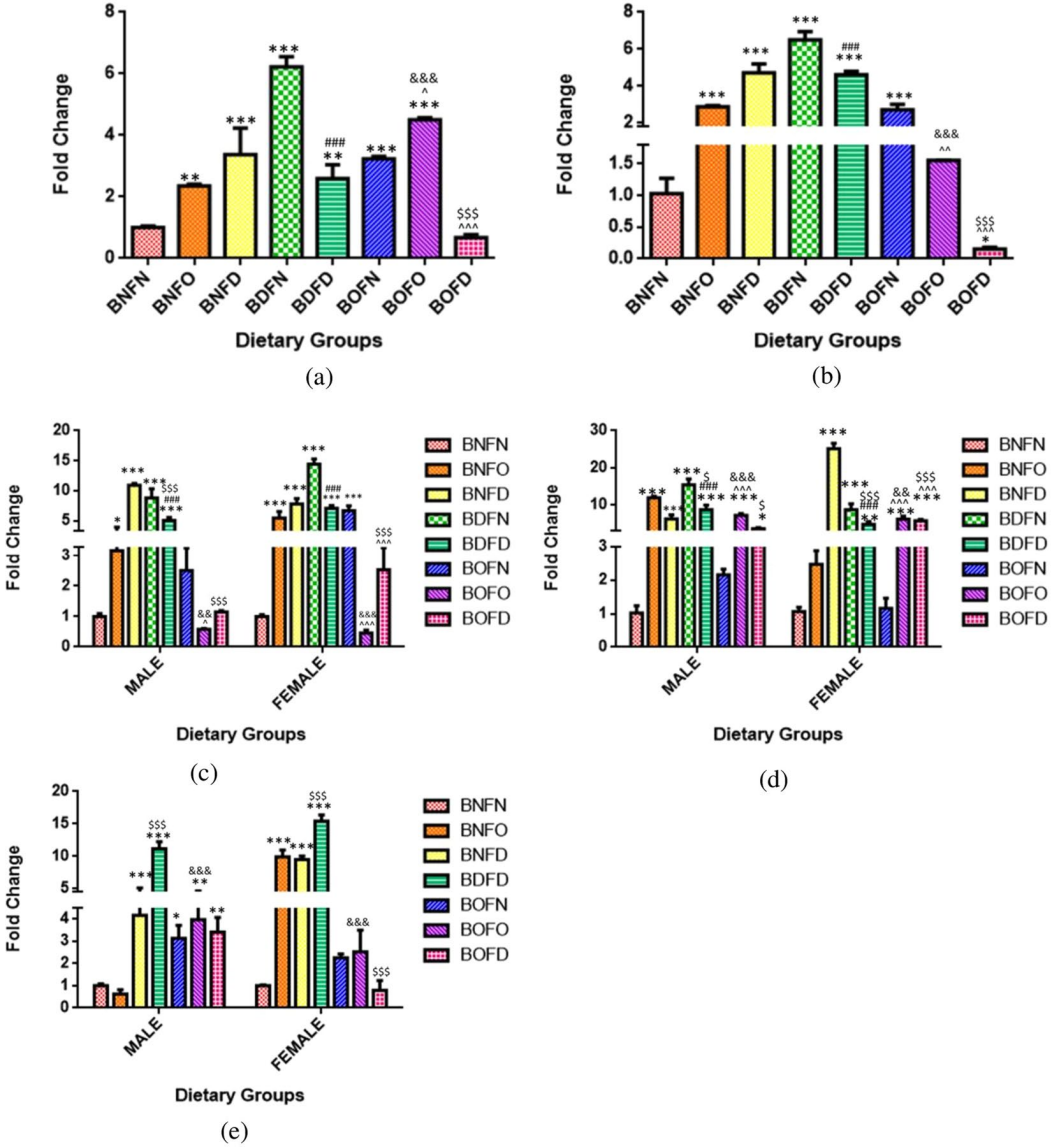
Quantification of miRNA-483 in maternal tissues. (**a**) Brain (F = 74.31, df = 7). (**b**) Placenta (F = 178.3, df = 7), fetal tissues. (**c**) Liver (Male) (F = 94.7, df = 7) (Female) (F = 106.3, df = 7). (**d**) Brain (Male) (F = 95.9, df = 7) (Female) (F = 243.9, df = 7). (**e**) Kidney (Male) (F = 80.1, df = 6) (Female) (F = 215.0, df = 6). *p < 0.05, **p < 0.01, ***p < 0.001 vs BNFN, ^#^p < 0.05, ^##^p < 0.01, ^###^p < 0.001 vs BDFN, ^p < 0.05, ^^p < 0.01, ^^^p < 0.001 vs BOFN, ^$^p < 0.05, ^$$^p < 0.01, ^$$$^p < 0.001 vs BNFD and ^&^p < 0.05, ^&&^p < 0.01, ^&&&^p < 0.001 vs BNFO. The data is presented as mean ± SD. (N = 3). B12 normal folate normal (BNFN), B12 normal folate over-supplemented (BNFO), B12 normal folate deficient (BNFD), B12 deficient folate normal (BDFN), B12 deficient folate over-supplemented (BDFO), B12 deficient folate deficient (BDFD), B12 over-supplemented folate normal (BOFN), B12 over-supplemented folate over-supplemented (BOFO), B12 over-supplemented folate deficient (BOFD).

In comparison to BNFN, the condition of folate deficiency combined with B12 normal (BNFD) and B12 deficiency (BDFD) increased the expression of miR-483 however; combination with B12 over-supplementation (BOFD) led to decrease in the placenta. Folate over-supplementation in combination with B12 normal (BNFO) as well as B12 over-supplementation (BOFO) overall led to an increase in expression of miR-483 in comparison to BNFN. (Fig. 6a–b).

Fetal tissues (F2). A significant interaction in folate and B12 was evident in the brain (male) (p < 0.001), brain (female) (p < 0.001), kidney (male) (p < 0.001), kidney (female) (p < 0.001), liver (male) (p < 0.001) and liver (female) (p < 0.001).

B12 deficiency in combination with folate deficiency (BDFD) as well as folate normal (BDFN) over-all led to an increase in transcript levels of miR-483 in all the fetal tissues regardless of sex (BNFN vs BDFN, BDFD). B12 over-supplementation combined to either state of folate led to an increase in the expression of miR-483 in the brain and kidney (male) whereas in females the mRNA levels were found to be increased in BOFN in the liver and with BOFO, BOFD in the brain (BNFN vs BOFN, BOFO, BOFD).

Condition of folate deficiency in combination with all the states of B12 (BNFD, BDFD, BOFD) resulted in increased expression of miR-483 in male and females’ fetuses in comparison to BNFN. Folate over-supplementation in combination to B12 normal (BNFO) increased the expression of miR-483 in the liver, brain (male) and kidney (females) and also in combination to B12 over-supplementation (BOFO) expression was increased in the brain along with kidney (male) as compared to BNFN (Fig. 6c–e).

#### Expression of miR-221

Maternal tissues (F1). A significant interaction between folate and B12 was present in the brain (p < 0.001), kidney (p < 0.01), liver (p < 0.001) and placenta (p < 0.001). The profile plots for the interaction of folate and B12 in maternal as well as fetal tissues are depicted in Supplementary Fig. 9.

B12 deficiency in combination with either condition of folate led to an increase in the expression of miR-221 except for brain in the BDFO group where it was found to be decreased (BNFN vs BDFN, BDFD, BDFO). B12 over-supplementation combined with either condition of folate led to a decrease in the mRNA levels if miR-221 in the placenta however, BOFO and BOFD led to an increase in the expression in the liver.

**Figure 7:**
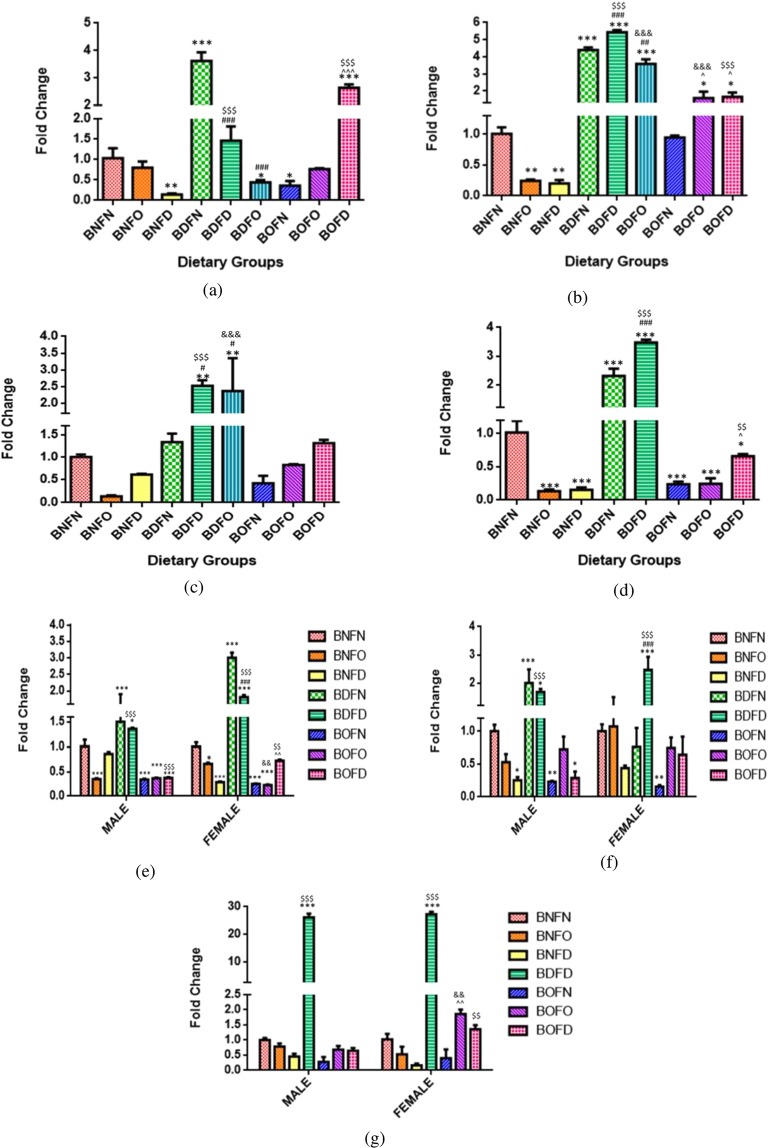
Quantification of miRNA-221 in maternal tissues. (**a**) Brain (F = 107.0, df = 8). (**b**) Liver (F = 291.0, df = 8). (**c**) Kidney (F = 17.03, df = 8). (**d**) Placenta (F = 317.3, df = 7), fetal tissues. (**e**) Liver (Male) (F = 31.5, df = 7), (Female) (F = 611.8, df = 7). (**f**) Brain (Male) (F = 33.8, df = 7), (Female) (F = 19.5, df = 7). (**g**) Kidney (Male) (F = 1461, df = 6), (Female) (F = 2745, df = 6) *p < 0.05, **p < 0.01, ***p < 0.001 vs BNFN, ^#^p < 0.05, ^##^p < 0.01, ^###^p < 0.001 vs BDFN, ^p < 0.05, ^^p < 0.01, ^^^p < 0.001 vs BOFN, ^$^p < 0.05, ^$$^p < 0.01, ^$$$^p < 0.001 vs BNFD and ^&^p < 0.05, ^&&^p < 0.01, ^&&&^p < 0.001 vs BNFO. The data is presented as mean ± SD. (N = 3). B12 normal folate normal (BNFN), B12 normal folate over-supplemented (BNFO), B12 normal folate deficient (BNFD), B12 deficient folate normal (BDFN), B12 deficient folate over-supplemented (BDFO), B12 deficient folate deficient (BDFD), B12 over-supplemented folate normal (BOFN), B12 over-supplemented folate over-supplemented (BOFO), B12 over-supplemented folate deficient (BOFD).

Condition of folate deficiency in combination with B12 normal (BNFD) led to a decrease in transcript levels of miR-221 whereas in combination with B12 deficiency (BDFD) expression was found to be increased, however, with BOFD expression was tissues specific as compared to BNFN. Folate over-supplementation combined with B12 normal (BNFO) and B12 deficiency (BDFO) led to a decrease in the mRNA levels of miR-221 except for liver and kidney where expression was increased in BDFO (Fig. 7a–d).

Fetal tissues (F2). Analysis of miR-221 gene revealed that there was a significant interaction between folate and B12 in the brain (male) (p < 0.01), brain (female) (p < 0.001), kidney (female) (p < 0.001), liver (male) (p < 0.01) and liver (female) (p < 0.001) except kidney (male) (p = 0.20).

In kidney (male), B12 deficiency (BD) independently led to an increase in the expression whereas B12 over-supplementation (BO) resulted in no change in the expression as compared to B12 normal (BN). Folate deficiency (FD) increased the expression of miR-221 in the kidney (male) however, with folate over-supplementation (FO) no change in the expression was observed when compared to folate normal (FN).

B12 deficiency combined with folate deficiency (BDFD) as well as folate normal (BDFN) resulted in increased expression of miR-221 in all the fetal tissues regardless of sex as compared to BNFN. B12 over-supplementation in combination with all states of folate led to reduced mRNA levels. (BNFN vs BOFN, BOFO, BOFD).

In comparison to BNFN, folate deficiency in combination with B12 deficiency (BDFD) led to an increase in transcript levels of miR-221 in all fetal tissues, however, in combination with B12 over-supplementation (BOFD) mRNA levels were found to be decreased in liver and brain (male) along with BNFD in the liver (female). Folate over-supplementation in combination with B12 normal as well as B12 over-supplementation led to reduced expression in the liver. (BNFN vs BNFO, BOFO) (Fig. 7e–g).

#### Expression of miR-133

Maternal tissues (F1). There was a significant interaction between folate and B12 in the brain (p < 0.001), kidney (p < 0.001), liver (p < 0.001) and placenta (p < 0.05). The profile plots for the interaction of folate and B12 in maternal as well as fetal tissues are depicted in Supplementary Fig. 10.

B12 deficiency in combination with folate normal (BDFN) resulted in increased transcript levels of miR133 in all tissues except placenta whereas in combination with folate deficiency (BDFD) levels were reduced in the brain and liver as compared to BNFN. B12 over- supplementation in combination with all conditions of folate (BOFN, BOFO, BOFD) overall led to reduced expression in maternal tissues as compared to BNFN.

**Figure 8:**
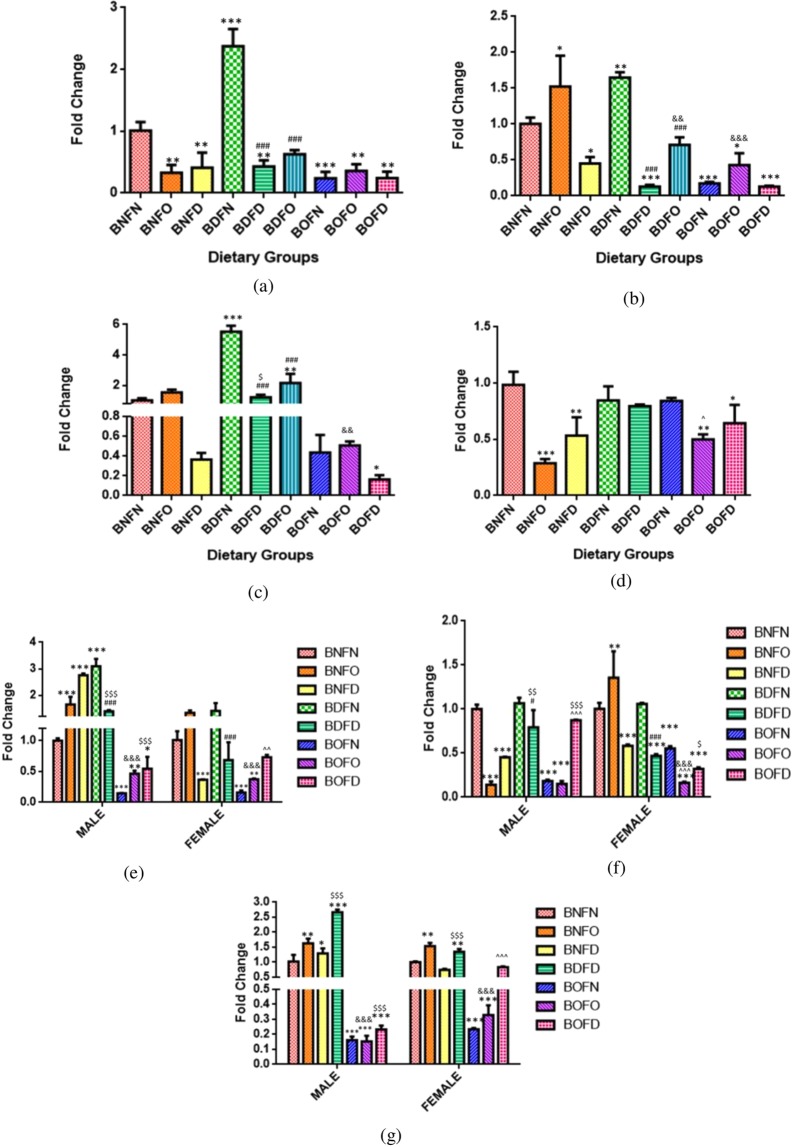
Quantification of miRNA-133 in maternal tissues. (**a**) Brain (F = 57.48, df = 8) (**b**) Liver (F = 37.49, df = 8). (**c**) Kidney (F = 117.0, df = 8). (**d**) Placenta (F = 14.60, df = 7), fetal tissues. (**e**) Liver (Male) (F = 133.9, df = 7), (Female) (F = 25.2, df = 7). (**f**) Brain (Male) (F = 79.7, df = 7), (Female) (F = 41.6, df = 7). (**g**) Kidney (Male) (F = 165.6, df = 6), (Female) (F = 179.7, df = 6). *p < 0.05, **p < 0.01, ***p < 0.001 vs BNFN, ^#^p < 0.05, ^##^p < 0.01, ^###^p < 0.001 vs BDFN, ^p < 0.05, ^^p < 0.01, ^^^p < 0.001 vs BOFN, ^$^p < 0.05, ^$$^p < 0.01, ^$$$^p < 0.001 vs BNFD and ^&^p < 0.05, ^&&^p < 0.01, ^&&&^p < 0.001 vs BNFO. The data is presented as mean ± SD. (N = 3). B12 normal folate normal (BNFN), B12 normal folate over-supplemented (BNFO), B12 normal folate deficient (BNFD), B12 deficient folate normal (BDFN), B12 deficient folate over-supplemented (BDFO), B12 deficient folate deficient (BDFD), B12 over-supplemented folate normal (BOFN), B12 over-supplemented folate over-supplemented (BOFO), B12 over-supplemented folate deficient (BOFD).

Under the conditions of folate deficiency, combination with all conditions of B12 (BNFD, BDFD, BOFD) led to a decrease in mRNA levels of miR-133 in maternal tissues as compared to control (BNFN). Condition of folate over-supplementation combined with B12 over-supplementation (BOFO) led to reduced mRNA levels of microRNA however, with B12 normal (BNFO) the levels were reduced in the brain and placenta and increased in the liver as compared to BNFN (Fig. 8a–d).

Fetal tissues (F2). Analysis of miR-133 gene in fetal tissues revealed that there was a significant interaction between folate and B12 in the brain (male) (p < 0.001), brain (female) (p < 0.001), kidney (male) (p < 0.01), kidney (female) (p < 0.001), liver (male) (p < 0.001) and liver (female) (p < 0.001).

B12 deficiency in combination with folate deficiency (BDFD) led to an increase in transcript levels of miR-133 in the kidney of fetuses of both sexes along with decrease in brain (female) however, in combination with folate normal (BDFN) miR-133 expression was found to be up-regulated in liver (male) as compared to BNFN. B12 over-supplementation combined with either state of folate (BOFN, BOFO, BOFD) led to a decrease in expression in fetuses of both the sexes in comparison to control (BNFN).

Condition of folate deficiency combined with B12 normal (BNFD) led to an increase in expression of miR-133 in the liver, kidney (male) however, expression was reduced in the brain of fetuses of both sex whereas combination with the state of B12 deficiency (BDFD), led to increased levels of miR-133 transcripts in kidney of fetuses of both sex and with B12 over-supplementation (BOFD) transcript levels were found to be decreased in the liver, kidney (male) and brain (female) as compared to BNFN. Folate over-supplementation combined with B12 over-supplementation (BOFO) led to a decrease in mRNA levels however, miR-133 expression was found to be increased with B12 normal (BNFO) in fetal tissues as compared to BNFN (Fig. 8e–g).

#### Effect of diet on global DNA methylation

Maternal tissues (F1). A significant interaction between folate and B12 was evident in global DNA methylation in the brain (p < 0.01), kidney (p < 0.001), liver (p < 0.001) and placenta (p < 0.001). The profile plots for the interaction of folate and B12 in maternal as well as fetal tissues are depicted in Supplementary Fig. 11.

B12 deficiency in combination with all the conditions of folate (BDFN, BDFD, BDFO) led to a decrease in levels of methylation in all maternal tissues except placenta as compared to BNFN. B12 over-supplementation combined with folate deficiency (BOFD) led to a decrease in global methylation except for placenta where it was found to be increased in comparison to BNFN.

Condition of folate deficiency combination with either condition of B12 (BNFD, BDFD, BOFD) decreased the levels of methylation in all maternal tissues except for placenta where it was found to be increased with BNFD and BOFD in comparison to BNFN. Folate over-supplementation combined with B12 deficiency (BDFO) led to a decrease in the methylation in all tissues when compared to BNFN (Supplementary Fig. 12A–D).

Fetal tissues (F2). There was a significant interaction between folate and B12 in the brain (male) (p < 0.05), liver (male) (p < 0.001) and liver (female) (p < 0.01) except brain (female) (p = 0.10), kidney (male) (p = 0.69), kidney (female) (p = 0.66) where interaction was not significant.

In the brain (female), kidney (male and female), B12 deficiency (BDFN) led to an increase in the methylation along with a decrease in the kidney (male) whereas B12 over-supplementation (BOFN) resulted in reduced methylation in all fetal tissues as compared to B12 normal (BNFN). Analyzing the independent effects of folate also revealed that in folate-deficient group (BNFD) the methylation was found to be decreased in the brain (female), kidney (male and female) however, with folate over-supplementation (BNFO) methylation was increased in all fetal tissues when compared to folate normal (BNFN).

On analyzing the effect of B12 deficiency by pairwise comparison in comparison to control (BNFN), combination with folate deficiency (BDFD) as well as folate normal (BDFN) led to an increase in global DNA methylation in the fetuses of both sexes. B12 over-supplementation combined with folate deficiency (BOFD) had no effect on methylation in comparison to BNFN.

Under the conditions of folate deficiency combination with B12 deficiency (BDFD) led to an increase in methylation whereas global DNA methylation was reduced in combination with B12 normal (BNFD) in all the fetal tissues regardless of sex in comparison to BNFN. Folate over supplementation) in combination with B12 normal (BNFO) led to an increase in methylation in fetuses of both sexes as compared to BNFN. (Supplementary Fig. 12E–G).

### Effect of diet on mRNA expression of DNMT1, DNMT3A and DNMT3B

#### Expression of DNMT1

Maternal tissues (F1). Analysis of DNMT1in mother by two way ANOVA revealed that there was a significant interaction between folate and B12 in the brain (p < 0.01), kidney (p < 0.001), liver (p < 0.001), and placenta (p < 0.001). The profile plots for the interaction of folate and B12 in maternal as well as fetal tissues are depicted in Supplementary Fig. 13.

B12 deficiency in combination with folate deficiency (BDFD) as well as over-supplementation (BDFO) led to increased mRNA levels of DNMT1 in the brain and liver whereas levels were decreased in the kidney (BDFN and BDFO) along with an increase in placenta (BDFN and BDFD) as compared to BNFN. B12 over-supplementation combined to folate normal (BOFN), as well as folate over-supplementation (BOFO), resulted in the downregulation of DNMT1 in comparison to BNFN.

Folate deficiency combined with either state of B12 (BNFD, BDFD, BOFD) resulted in an increase in the transcript levels of DNMT1 as compared to BNFN. Folate over-supplementation combined with B12 normal (BNFO) as well as B12 over-supplementation (BOFO) led to no change in expression of DNMT1 in maternal tissues except kidney where expression was found to be increased in BNFO and decreased in BOFO (Supplementary Fig. 14A–D).

Fetal tissues (F2). There was a significant interaction between folate and B12 in the brain (male) (p < 0.001), brain (female) (p < 0.01), kidney (male) (p < 0.01), liver (male) (p < 0.001) and liver (female) (p < 0.001) except kidney (female) (p = 0.82) where interaction was not significant.

In kidney (female), B12 deficiency (BD) independently led to an increase in the expression of DNMT1 whereas with B12 over-supplementation (BO) transcript levels of DNMT1 were not changed as compared to B12 normal (BN). Folate deficiency (FD) led to an increase in the mRNA levels of DNMT1 in the kidney (female), however, with folate over-supplementation (FO) levels were not changed when compared to folate normal (FN).

B12 deficiency in combination with folate deficiency (BDFD) led to an increase in mRNA levels of DNMT1 in all fetal tissues regardless of sex whereas no effect was seen in combination with folate normal (BDFN)in comparison to BNFN. B12 over-supplementation combined with either state of folate did not affect the expression of DNMT1 in fetuses compared to BNFN.

Folate deficiency combined with B12 deficiency (BDFD) increased the mRNA levels of DNMT1 all fetal tissues regardless of sex whereas in combination with the state of B12 normal (BNFD) the expression of DNMT1 was increased in the liver and the brain (female) in comparison to BNFN. Under the conditions of folate over-supplementation combination with either state of B12 (BNFO, BOFO) had no effect on the expression as compared to BNFN (Supplementary Fig. 14E–G).

#### Expression of DNMT3A

Maternal tissues (F1). There was a significant interaction between folate and B12 in the brain (p < 0.01), kidney (p < 0.01), liver (p < 0.001) and placenta (p < 0.001). The profile plots for the interaction of folate and B12 in maternal as well as fetal tissues are depicted in Supplementary Fig. 15.

B12 deficiency combined with folate deficiency (BDFD) led to an increase in DNMT3A expression in all maternal tissues except kidney where no change in expression was observed whereas combination with folate normal (BDFN) and over-supplementation (BDFO) resulted in reduced expression in the kidney in comparison to BNFN. B12 over-supplementation, combined with folate deficiency (BOFD) increased the expression of DNMT3A in all tissues whereas in combination to folate normal (BOFN) expression was increased in kidney and placenta compared to BNFN.

In comparison to BNFN, the condition of folate deficiency combined with either state of B12 (BNFD, BDFD, BOFD) overall led to an increase in expression. Folate over-supplementation, (BNFN vs BNFO, BOFO) in combination with B12 normal (BNFO) as well as B12 over-supplementation (BOFO) did not affect the expression of DNMT3A (Supplementary Fig. 16A–D).

Fetal tissues (F2). There was a significant interaction between folate and B12 in the brain (female) (p < 0.01), kidney (male) (p < 0.05) and liver (male) (p < 0.01) except brain (male) (p = 0.39), kidney (female) (p = 0.13) and liver (female) (p = 0.16) where interaction was not significant.

In brain (male), kidney (female) and liver (female), on analyzing the independent effects of B12, it was observed that in B12 deficient group (BDFN), the mRNA levels of DNMT3A were significantly increased in brain (male), and liver (female) whereas with B12 over-supplementation levels were found to be increased in brain (male) as compared to B12 normal (BNFN). Folate deficiency (BNFD) led to an increase in transcript levels of DNMT3A in the kidney, liver (females) however, with folate over-supplementation (BNFO) expression was increased in the kidney (female) when compared to folate normal (BNFN).

B12 deficiency in combination with folate normal (BDFN) had no effect on the expression of DNMT3A whereas combination with folate deficiency (BDFD) led to an increase in mRNA levels of DNMT3A in the liver of fetuses of both sexes along with the brain (female) in comparison to BNFN. B12 over-supplementation combined with folate normal (BOFN) as well as folate over-supplementation (BOFO) increased the expression of DNMT3A in fetal tissues whereas in combination to folate deficiency (BOFD) expression was increased in female fetal tissues as compared to BNFN.

In comparison to BNFN, folate deficiency combined with either state of B12 (BNFD, BDFD, BOFD) overall led to an increase in DNMT3A levels in all fetal tissues regardless of sex. Folate over-supplementation combined with B12 normal (BNFO) as well as B12 over-supplementation (BOFO) increased the transcripts levels of DNMT3A in the liver (male) and kidney (female) (Supplementary Fig. 16E–G).

#### Expression of DNMT3B

Maternal tissues (F1). There was a significant interaction of folate and B12 in DNMT3B in the brain (p < 0.01), kidney (p < 0.001), liver (p < 0.001) and placenta (p < 0.001). The profile plots for the interaction of folate and B12 in maternal as well as fetal tissues are depicted in Supplementary Fig. 17.

B12 deficiency in combination with folate deficiency (BDFD) increased transcript levels of DNMT3B whereas in combination with folate normal (BDFN) levels were decreased in the brain and increased in the liver as compared to BNFN. B12 over-supplementation in combination with folate deficiency (BOFD) led to an increase in the mRNA levels of DNMT3B in all tissues along with an increase in the placenta in BOFO group and decrease in the brain in BOFN group as compared to control (BNFN).

In comparison to BNFN, folate deficiency in combination with all conditions of B12 (BNFD, BDFD, BOFD) over-all led to an increase in expression. Folate over-supplementation combined with B12 normal (BNFO) led to an increase in expression in kidney and placenta in comparison to BNFN (Supplementary Fig. 18A–D).

Fetal tissues (F2). A significant interaction between folate and B12 in DNMT3B was found in the brain (female) (p < 0.001), kidney (male) (p < 0.01), kidney (female) (p < 0.001), liver (male) (p < 0.001) and liver (female) (p < 0.001) except brain (male) (p = 0.20) where interaction was not significant.

In the brain (male) on analyzing the independent effects of B12, it was observed that in B12 deficient group (BD) the expression of DNMT3B was increased however with B12 over-supplementation (BO) no change was observed as compared to B12 normal (BN). Folate deficiency (FD) increased in the transcript levels of DNMT3B however, with folate over-supplementation (FO) transcript levels were not changed in the brain (male) when compared to folate normal (FN).

B12 deficiency in combination with folate deficiency (BDFD) resulted in an increase in levels of DNMT3B in all the fetal tissues regardless of sex whereas no change was observed with folate normal (BDFN) as compared to BNFN. B12 over-supplementation, combined with either state of folate (BOFD, BOFO) overall led to an increase in the expression as compared to BNFN.

In comparison to BNFN, folate deficiency combined with all conditions of B12 (BNFD, BDFD and BOFD) led to an increase in the expression. However, folate over-supplementation combined with either state of B12 (BNFO, BOFO) had no effect on the expression in comparison to control (BNFN). (Supplementary Fig. 18E–G).

## Discussion

Previous studies carried out in this field had demonstrated the individual effects of folate and B12 deficiencies on the pregnancy outcome but there are very few studies that have focused on studying the combined effects of folate and B12 during pregnancy. To the best of our knowledge, this is the first study that tried to address how the dietary imbalance of both the vitamins during pregnancy can influence the health of the offspring and cause alteration in the expression of genes. In our study, the group with high folate and low B12 status was the worst affected condition, where most of the animals had died. Dietary imbalance of folate and B12 affected the expression of folate transporters. Interestingly, the expression of folate transporters was also affected by the B12 deficiency and that of B12 transporters/proteins was affected by folate deficiency as well as over-supplementation. The expression of DNMTs was significantly increased upon the deficiency of folate and B12 whereas global DNA methylation was found to be decreased with deficiency of both the vitamins in the maternal tissues except placenta. Global DNA methylation was increased in the placenta as well as in the fetal tissues. Dietary imbalance of folate and B12 also led to changes in the expression of the studied miRNAs.

Poor dietary intake is one of the predominant factors which results in B12 deficiency in a significant segment of population^38^. It is well known that damaging effects of deficiency of B12 can be masked by an excess amount of folic acid by correcting the state of megaloblastic anemia^39^. Moreover, in the developing countries like India, people have a greater preference towards a vegetarian diet due to social and religious beliefs and are more likely to be B12 deficient^40,41^. Moreover, in these countries, folic acid is supplemented during the preconception period and throughout the gestation period at a much higher level than that recommended by WHO guidelines (600 µg vs 5 mg)^42^. Such practices might lead to a state of high folate with low vitamin B12 levels in pregnant women. In this study, we tried to evaluate the combined effects of altered ratios of both the vitamins i.e. folate and B12 in maternal/parental diet on the expression of folate and B12 transporters, DNA methylation and miRNA in maternal and fetal tissues.

As expected, serum levels of folate and vitamin B12 were significantly decreased under the conditions of dietary deficiency and increased with supplementation of the respective vitamins. Serum levels of homocysteine were significantly increased under the conditions of folate deficiency. Body weight of the mice was decreased maximally in BDFO group which showed the highest mortality rate too. The observations are in support of the literature explaining the ill effects of excess folate in combination with low B12^12^. Moreover, none of the females in the BDFO group got pregnant.

In the present study, folate deficiency resulted in increased expression of folate transporters RFC, PCFT and FOLR1 required to mediate transport into the cell however, over supplementation of folate was associated with a decrease in the expression. Interestingly, in this study B12 deficiency (BDFN) too led to an increased expression of folate transporters which can be due the compensatory mechanism due to altered dietary ratio of folate and B12. (Figs. 1–3). Previous studies carried out in our lab^43^ and other studies^44,45^ have reported that deficiency of folate can lead to an increase in intestinal uptake of folate thereby increasing the expression of folate transporters.

In the case of B12 transporters, B12 binding protein, namely LMBRD1 was increased with B12 deficiency in the maternal as well as fetal tissues. In the maternal brain, however, it was interesting to note that folate deficiency (BNFD) independently led to an increased expression and B12 deficiency (BDFN) was associated with decreased expression. This decrease in the expression induced by B12 deficiency might reduce the availability of vitamin B12 as LMBRD1 gene mutations are associated with the production of abnormal non-functional short LMBD1 protein and prevent the release of vitamin B12 from lysosomes^46,47^. B12 over-supplementation as well as folate over-supplementation overall, were associated with an increase in transcript levels of LMBRD1 (Fig. 4).

TC-II is mainly involved in cellular delivery and absorption of vitamin B12^48^. B12 deficiency in combination with all conditions of folate increased the expression of TC-II in the maternal as well as fetal tissues except kidney, where it was decreased. Previous studies have suggested that the kidney is the major site of daily vitamin B12 processing where TC-II bound B12 (holo-TCII which delivers B12 to all the cells) is filtered and reabsorbed in the proximal tubules via megalin, the multi-ligand receptor for TCII^49^. Thus, reduced expression of TC-II under dietary deficiency of B12 may lead to an alteration in the metabolism of TC-II. (Fig. 5).

Further, we analyzed the expression of miRNAs which, in previous studies, were found to be altered with the diet. Over-expression of miR-483, has been shown to be associated with hepatocellular carcinoma^50^. The IGF2 mRNA itself is transcriptionally up-regulated by miR-483. In our study, miRNA-483 was found to be up-regulated under all the conditions of folate and B12 in the maternal and fetal tissues. (Fig. 6). It has been previously reported that increased miR-483 expression limits the storage of lipids in adipose tissue and is linked to insulin resistance and type 2 diabetes^31,51^. This finding along with our observations of increased expression of miRNA-483 suggests that altered dietary ratio of folate and B12 might predispose an individual to type 2 diabetes.

Another microRNA which is known to be altered under methyl deficient conditions is miR-221. B12 deficiency combined with either state of folate led to an upregulation in the expression of miR-221 in the maternal and fetal tissues with the maximum fold in the kidney (F2). (Fig. 7). Increased miR-221 expression was earlier found to be associated with renal cell carcinoma where it inhibited tumor suppressor gene TIMP2^52,53^. Hence, upregulated miR-221 expression under our setting of B12 deficiency might increase the susceptibility of mice towards the development of renal cell carcinoma.

As per the “Pune maternal nutrition study”, low maternal vitamin B12 and high erythrocyte folate concentrations during pregnancy were associated with augmented insulin resistance in the offspring. A previous study has established that downregulation of miR-133 expression was found to be associated with pathogenesis leading to cardiomyopathy associated with diabetes^35^. These findings intrigued us to determine miR-133 expression in our experimental setup. We found that all the conditions of dietary folate and B12 led to a decrease in the expression of miR-133 except in BDFN in the maternal tissues. In fetal tissues, the change in expression was tissue- and sex-specific. (Fig. 8). Thus, diet-associated alterations in the expression of this micro-RNA may be involved in the pathogenesis of metabolic diseases.

DNA methylation quantification at the global level revealed that deficiency of both the vitamins resulted in a decrease in methylation in all maternal tissues except the placenta where it was increased in BNFD and BOFD groups. A previous study from our lab by Rahat *et al*.^44^ suggested that folate deficiency resulted in the hypo-methylation of CpGs within the DNMT1 and DNMT3A in association with their overexpression and increased global DNA methylation which could be a possible explanation of increased methylation observed in the placenta in our study. However, hypomethylation as observed in other tissues might be due to decreased level of S-adenosyl-methionine (SAM)^45^. In fetal tissues, methylation was overall found to be increased in all conditions except folate-deficient condition (BNFD) where methylation was decreased. (Supplementary Fig. 12). The results suggest that dietary imbalance in the ratio of folate and B12 resulted in altered global DNA methylation.

DNA methyltransferase enzymes namely DNMT1, DNMT3A and DNMT3B catalyze DNA methylation reactions^54^. The dietary combination of folate and B12 deficiency overall led to an increase in the expression of DNMT1 (Supplementary Fig. 14), DNMT3A (Supplementary Fig. 16) and DNMT3B (Supplementary Fig. 18) in maternal and fetal tissues. Our results are consistent with a study which has reported up-regulation of DNMTs under folate and methionine deficient diet^24^. Early life programming is dependent on genomic imprinting which is established in a parent of origin- specific manner based on DNA methylation and any alterations in methylation state can affect the imprinting status of developing fetus^55^. This suggests that the maternal dietary factors regulating the state of DNA methylation and DNMTs are imperative to study considering the importance of imprinting genes in the development.

Therefore, the results revealed that altered dietary ratios of folate and B12 can have more severe effects than the individual deficiencies. The study supports the recommendation to maintain a balance of folate and B12 in the peri-conception period and during pregnancy. However, there are certain limitations to this study. Unexplained deaths of females fed with B12 deficient folate over-supplemented diet (BDFO) and infertility observed in the same dietary group warrants further studies. We could not determine the size and weight of the pups (F2). Dietary fiber sources such as cellulose and pectin used in different dietary formulations deficient diets can lead to alterations in the microbiome which was also not assessed in the present study.

## Materials and Method

This study was carried out in accordance with the CPCSEA guidelines (committee for the purpose of control and supervision of experimental animals), Govt. of India. The experimental procedures were approved by the Post Graduate Institute of Medical Education & Research (PGIMER) Institutional Animal Ethics Committee (PGIMER/IAEC/465).

### Animals

C57BL/6 male (n = 36) and female mice (n = 108) of average weight 15–20 g were obtained from animal house facility of PGIMER, Chandigarh. The mice were randomized into 4 groups, each group consisted of 4 males and 12 females. They were maintained at 22 °C in a controlled 12-hr light and 12 h dark cycle with appropriate ventilation system.

### Diets

The composition of the control and the treatment diets was as per AIN 93 purified diets for laboratory rodents^56^. A total of 9 diets were formulated based on different combinations of folate and B12 namely, B12 normal folate normal (BNFN), B12 normal folate over-supplemented (BNFO), B12 normal folate deficient (BNFD), B12 deficient folate normal (BDFN), B12 deficient folate over-supplemented (BDFO), B12 deficient folate deficient (BDFD), B12 over-supplemented folate normal (BOFN), B12 over-supplemented folate over-supplemented (BOFO), B12 over-supplemented folate deficient (BOFD). The diets used in study were same as used in study by Kulkarni *et al*., 2011^37,57–59^.

Folate deficiency was established by omitting folate from the diet and adding succinyl sulfathiazole (10 g/kg). The lowest level, i.e. 2 mg/kg represents the normal level of folic acid while 8 mg/kg is 4 times the requirement of a normal mice represents over-supplemented folate levels.

B12 deficiency was induced by adding pectin as a dietary fiber. Control and over -supplemented B12 diets contained 0.025 mg/kg and 0.1 mg/kg of added vitamin B12 respectively with cellulose as dietary fiber, whereas B12 deficient diet has no added B12 and pectin as dietary fiber. Pectin binds the intrinsic factor in the intestine and makes vitamin B12 less bioavailable

### Breeding (F1 generation)

Female mice divided into 9 groups were fed diet accordingly for the period of 4 weeks and the male mice received normal diet *ad libitum*.

The male and female mice were allowed to mate, and mating was confirmed by detection of vaginal plug and this was denoted as day 0, and pregnant mice (total of 6 pregnant females in each group) were individually housed. This was denoted as F0 generation and fetus born to F0 mothers were denoted as F1 generation fetus.

For F1 generation to grow, 3 female pups and 1 male pup from 6 pregnant females (F0) in each group was used i.e. 6 males and 18 females in each group (F1).

The grown fetuses (F1 generation) were weaned at 3 weeks of age and then continued same diet as fed to their mothers for 6 weeks. Mating was carried out (4 males and 12 females in each group) among the same dietary group, and pregnant mice (6 mothers in each group) were individually housed and sacrificed on day 20 of gestation.

The fetal tissues (F2) were pooled according to sex after sex determination from each female of the group. Fetuses (3male and 3 female) born to each mother in a group were pooled according to sex in each group so that we have 6 male pooled fetal tissues (brain, liver and kidney) and 6 female pooled fetal tissues (brain, liver and kidney) in each group from 6 pregnant females. The work flow of the study is depicted in Supplementary Fig. 19.

Blood was collected, serum isolated and stored at −80 °C for biochemical parameters. Maternal (F1) and fetal tissues (F2) were isolated and kept in −80 °C for further use.

### Fetal sex determination

DNA was isolated from tail of fetuses’ using HiPura Mammalian Genomic DNA purification Kit (Hi-media, India) as per manufacturer’s instruction for fetal sex determination. Absorbance (A260/280) of 1.8 was taken as acceptable value. Conventional PCR was carried out for detection of the male-specific gene, SRY, with the autosomal gene, MYOGENIN serving as a positive control. The primers used are shown in Supplementary Table 1.

### Maternal serum folic acid, vitamin B12 and homocysteine levels

Serum folic acid levels were measured by electrochemiluminescence method on cobas ECLIA e 411 (Roche Diagnostics GmbH, Mannheim, Germany) whereas serum vitamin B12 levels were measured using ELISA kit Mouse vitamin B12 kit according to the manufacturer’s protocol (CUSABIO, China) and homocysteine level was determined by using ADVIA Centaur Hcy assay (ADVIA Centaur, Bayer, USA).

### Gene expression

To quantify mRNA expression of target genes, Real-time PCR and comparative Ct method (ΔΔCT)^60^ was used. Total RNA was extracted by using TRIzol reagent (Ambion, Life Technologies Corporation, CA, USA). RNA (1 µg) was reverse transcribed using cDNA synthesis kit (MBI Fermentas, Life Sciences, USA) and Real-time PCR (Life Technologies Corporation, California) was performed using gene specific primers as listed in Supplementary Table 2 and GAPDH was used as endogenous control. The data was analyzed, and the relative expression was calculated. The melt curves are depicted in (Supplementary Fig. 20).

### miRNA expression studies

TRIzol (Ambion, Life Technologies Corporation, California) was used for isolation of total RNA. cDNA conversion was followed by using miscript II RT kit (Qiagen, Germany) according to manufactures guidelines. Expression of miRNA was quantified by using miRNA specific primers using real time PCR (Supplementary Table 3). The relative expression of each miRNA was normalized against SNORD 70. The melt curves for microRNAs are depicted in Supplementary Fig. 20.

### Statistical methods

Data is presented as mean ± SD. Statistical significance was set at p < 0.05. Two-way ANOVA was applied to study the interaction as well as independent effects of folate and B12 among various dietary groups of folate and B12 and if interaction was present then we applied one-way analyses of variance (ANOVA) (α = 0.05) followed by Tukey post-hoc test. GraphPad Prism (v.6.0.1) and IBM SPSS program (v.23, NY, USA) were used for statistical analysis.

## Supplementary information

supplementary figures and tables

## Data Availability

No datasets were generated or analyzed during the current study.
