# Supplementary material for: Effect of imbalance in folate and vitamin B12 in maternal/parental diet on global methylation and regulatory miRNAs
Source: Sci Rep. 2019 Nov 26;9:17602. doi: 10.1038/s41598-019-54070-9 (PMC6879517; doi:10.1038/s41598-019-54070-9)
Supplement: Supplementary file 1 — supplementary figures and tables [file 41598_2019_54070_MOESM1_ESM.pdf]

Effect of imbalance in folate and vitamin B12 in maternal/ parental diet on global methylation and regulatory miRNAs

Aatish Mahajan[1], Divika Sapehia[1], Shilpa Thakur[1], Mohan Raj P.S[1], Rashmi Bagga[2] and Jyotdeep Kaur[1]*

[1] Department of Biochemistry, Postgraduate Institute of Medical Education and Research, India

[2]Department of Obstetrics and Gynecology, Postgraduate Institute of Medical Education and Research, India

*Corresponding author

Prof. Jyotdeep Kaur

Department of Biochemistry

Research Block-A,

PGIMER, Chandigarh, PIN-160012

Tel: 91-172-2755181

E-mail: jyotdeep2001@yahoo.co.in

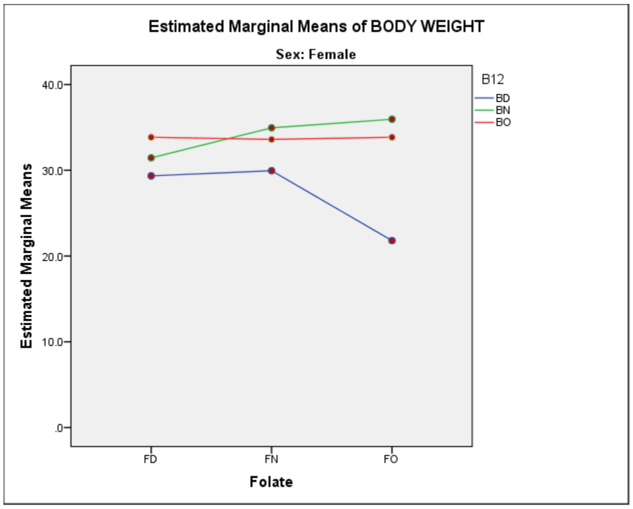

Supplementary figure 1 : Profile plot of body weight (F= 57.7, df= 8)

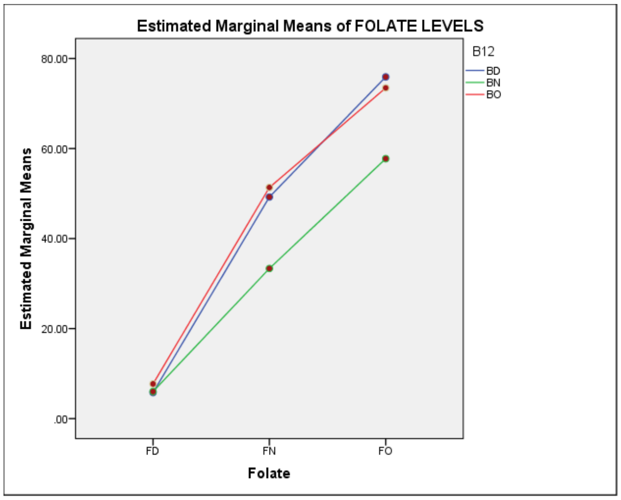

(A)

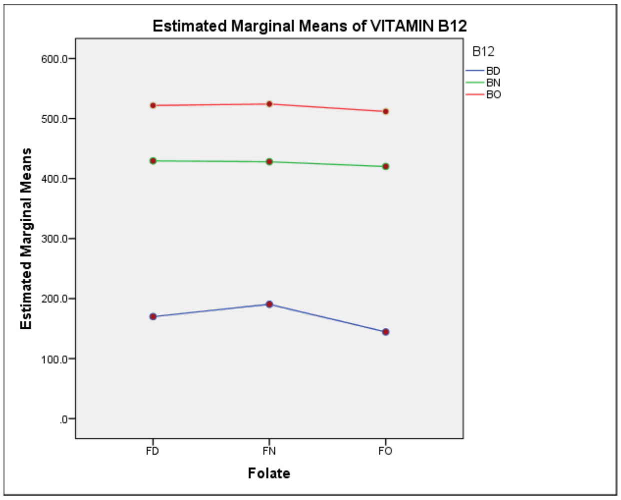

(B)

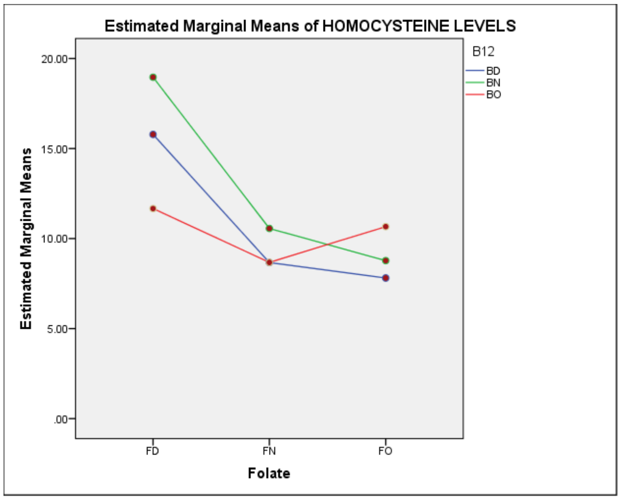

(C)

Supplementary figure 2 : Profile plots of serum levels of folate, B12 and homocysteine (A) Folate (F= 144.3, df=8) (B) Vitamin B12 (F= 3500.2, df=8) (C) Homocysteine (F= 103.1, df=8)

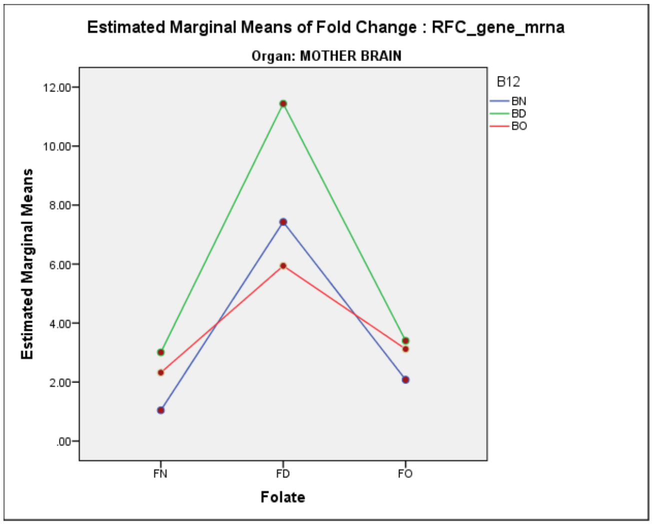
Estimated Marginal Means of Fold Change : RFC_gene_mrna
Organ: MOTHER BRAIN
B12
BN
BD
BO
12.00
10.00
8.00
6.00
4.00
2.00
.00
Estimated Marginal Means
FN
FD
FO
Folate

(A)

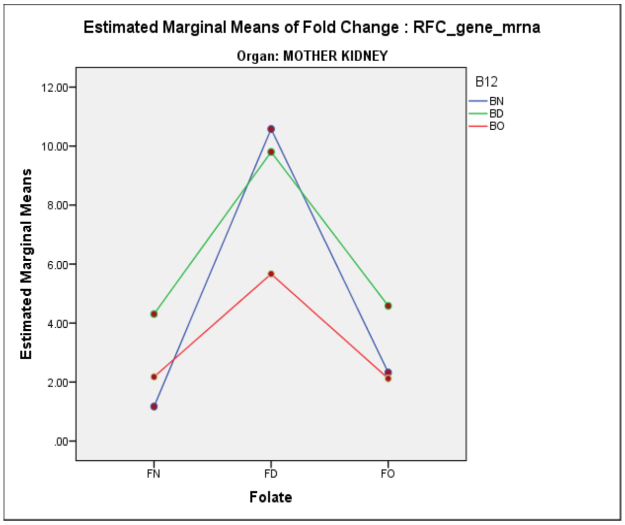
Estimated Marginal Means of Fold Change : RFC_gene_mrna
Organ: MOTHER KIDNEY
B12
BN
BD
BO
12.00
10.00
8.00
6.00
4.00
2.00
.00
Estimated Marginal Means
FN
FD
FO
Folate

(B)

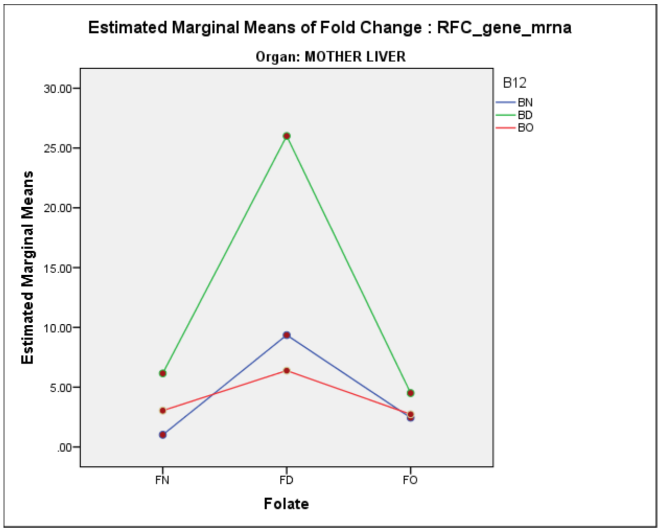
Estimated Marginal Means of Fold Change : RFC_gene_mrna
Organ: MOTHER LIVER
B12
BN
BD
BO
30.00
25.00
20.00
15.00
10.00
5.00
.00
Estimated Marginal Means
FN
FD
FO
Folate

(C)

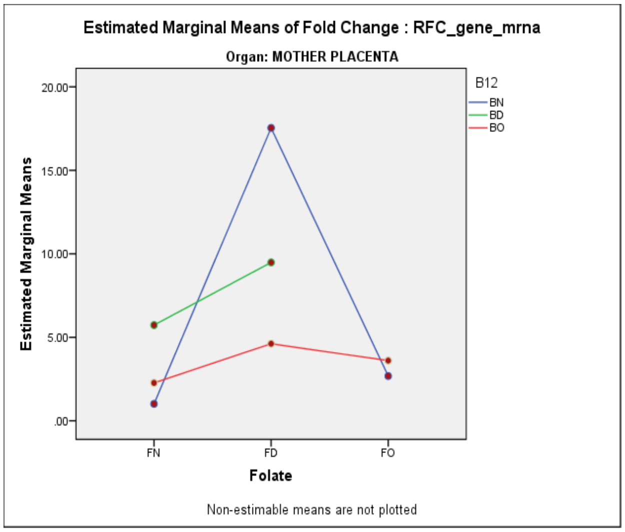
Estimated Marginal Means of Fold Change : RFC_gene_mrna
Organ: MOTHER PLACENTA
B12
BN
BD
BO
20.00
15.00
10.00
5.00
.00
Estimated Marginal Means
FN
FD
FO
Folate
Non-estimable means are not plotted

(D)

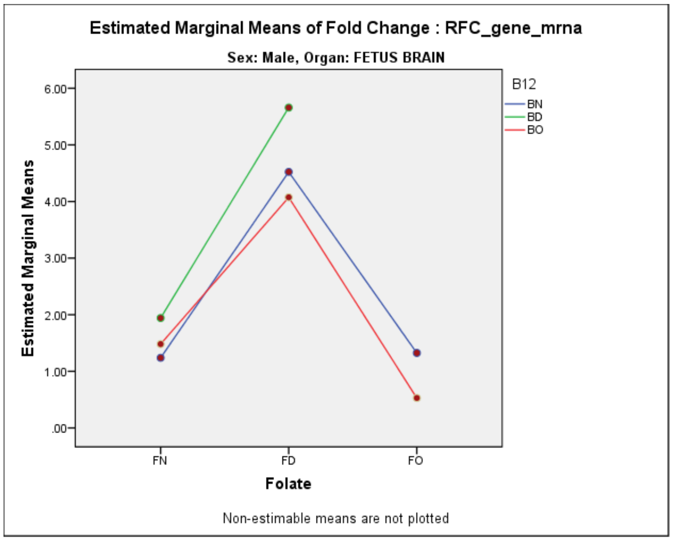
Estimated Marginal Means of Fold Change : RFC_gene_mrna
Sex: Male, Organ: FETUS BRAIN
B12
BN
BD
BO
6.00
5.00
4.00
3.00
2.00
1.00
.00
Estimated Marginal Means
FN
FD
FO
Folate
Non-estimable means are not plotted

E (i)

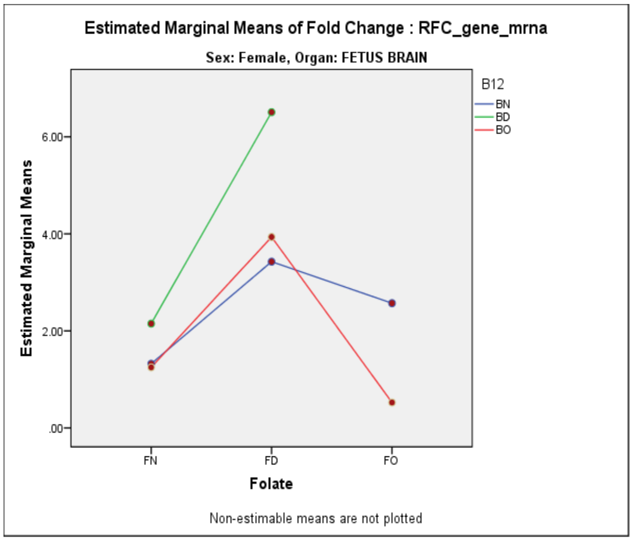
Estimated Marginal Means of Fold Change : RFC_gene_mrna
Sex: Female, Organ: FETUS BRAIN
B12
BN
BD
BO
6.00
4.00
2.00
.00
Estimated Marginal Means
FN
FD
FO
Folate
Non-estimable means are not plotted

E (ii)

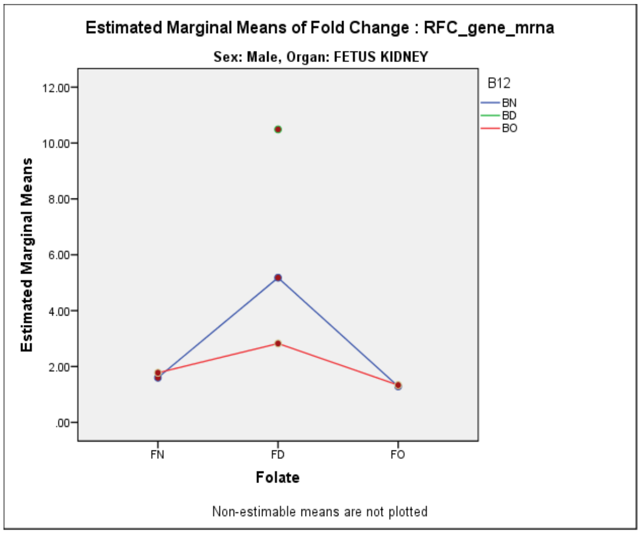

F (i)

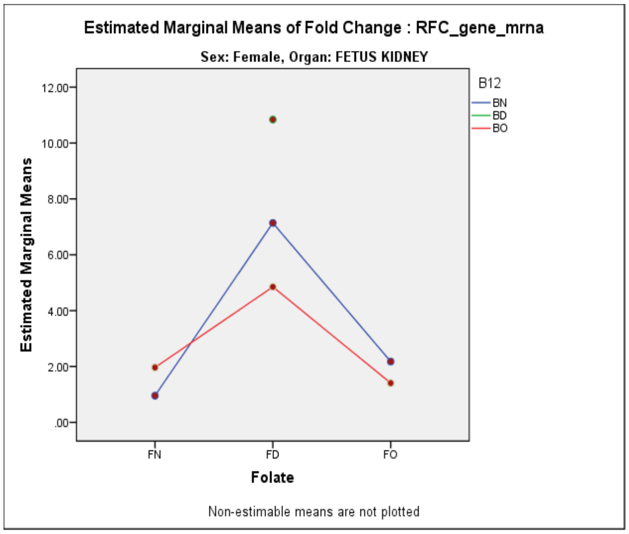

F (ii)

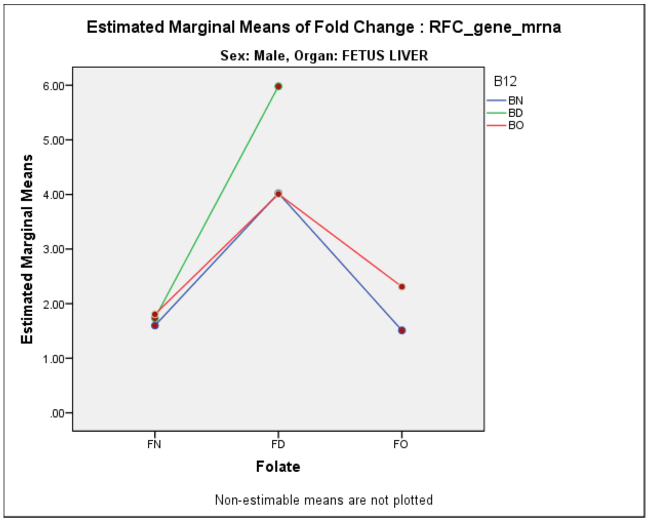

G (i)

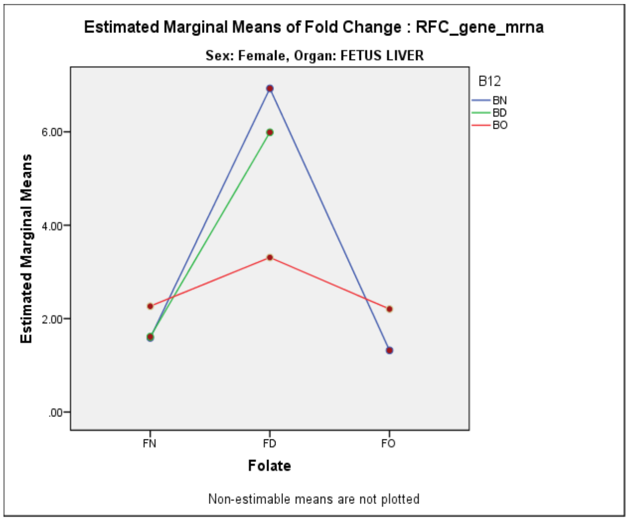

G (ii)

Supplementary figure 3 : Profile plots of mRNA expression of RFC in maternal tissues (A) Brain (F=6.0, df=4) (B) Kidney (F=25.9, df=4) (C) Liver (F=270.5, df=4) (D) Placenta (F=50.4, df=3), fetal tissues (E) (i) Brain (Male) (F=1.29, df=3) (ii) Brain (Female) (F=14.9, df=3), (F) (i) Kidney (Male) (F= 6.3, df=2) (ii) Kidney (Fe-male) (F=8.7, df=2), (G) (i) Liver (Male) (F=5.13, df=3) (ii) Liver (Fe-male) (F=18.7,df=3)

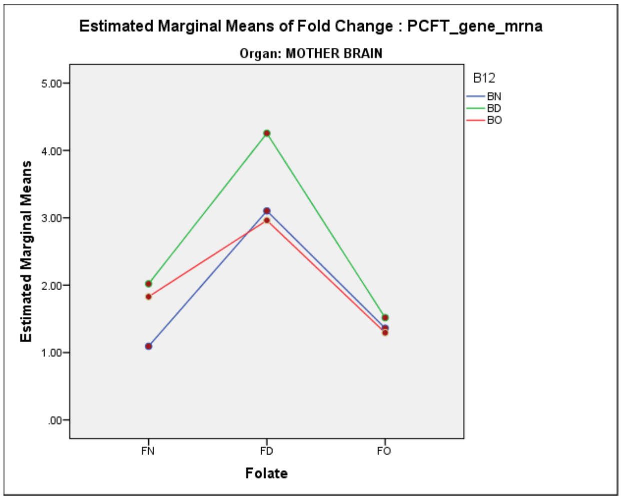
Estimated Marginal Means of Fold Change : PCFT_gene_mrna
Organ: MOTHER BRAIN
Estimated Marginal Means
5.00
4.00
3.00
2.00
1.00
.00
FN
FD
FO
Folate
B12
BN
BD
BO

(A)

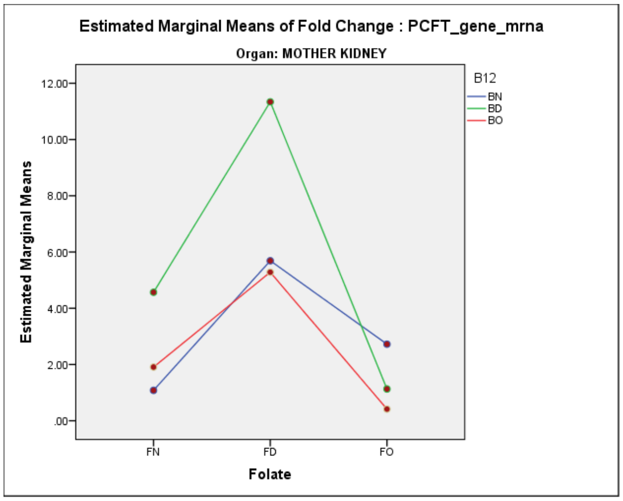
Estimated Marginal Means of Fold Change : PCFT_gene_mrna
Organ: MOTHER KIDNEY
Estimated Marginal Means
12.00
10.00
8.00
6.00
4.00
2.00
.00
FN
FD
FO
Folate
B12
BN
BD
BO

(B)

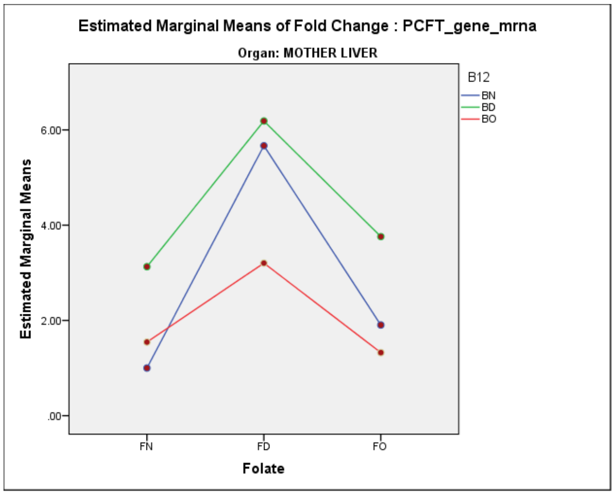
Estimated Marginal Means of Fold Change : PCFT_gene_mrna
Organ: MOTHER LIVER
Estimated Marginal Means
6.00
4.00
2.00
.00
FN
FD
FO
Folate
B12
BN
BD
BO

(C)

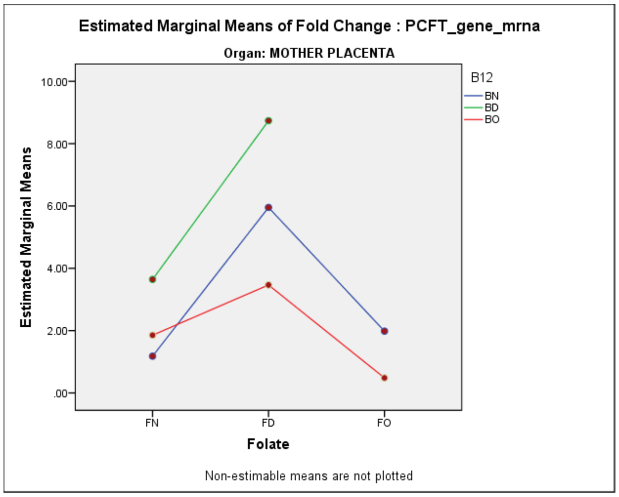
Estimated Marginal Means of Fold Change : PCFT_gene_mrna
Organ: MOTHER PLACENTA
Estimated Marginal Means
10.00
8.00
6.00
4.00
2.00
.00
FN
FD
FO
Folate
Non-estimable means are not plotted
B12
BN
BD
BO

(D)

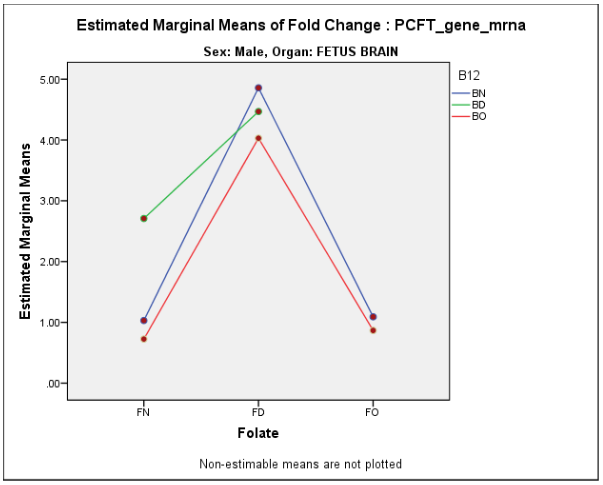
Estimated Marginal Means of Fold Change : PCFT_gene_mrna
Sex: Male, Organ: FETUS BRAIN
Estimated Marginal Means
5.00
4.00
3.00
2.00
1.00
.00
FN
FD
FO
Folate
Non-estimable means are not plotted
B12
BN
BD
BO

E (i)

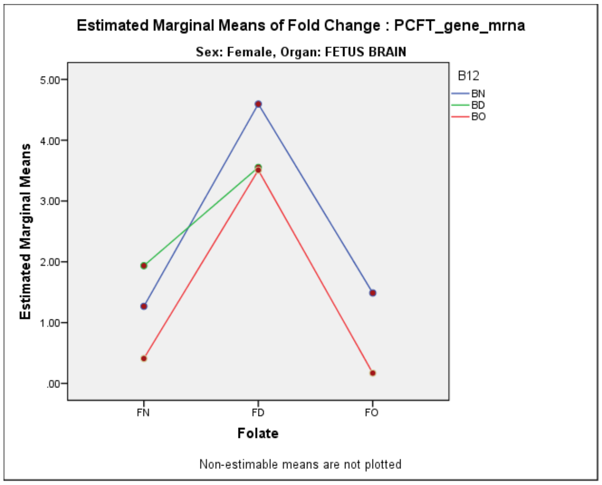
Estimated Marginal Means of Fold Change : PCFT_gene_mrna
Sex: Female, Organ: FETUS BRAIN
Estimated Marginal Means
5.00
4.00
3.00
2.00
1.00
.00
FN
FD
FO
Folate
Non-estimable means are not plotted
B12
BN
BD
BO

E (ii)

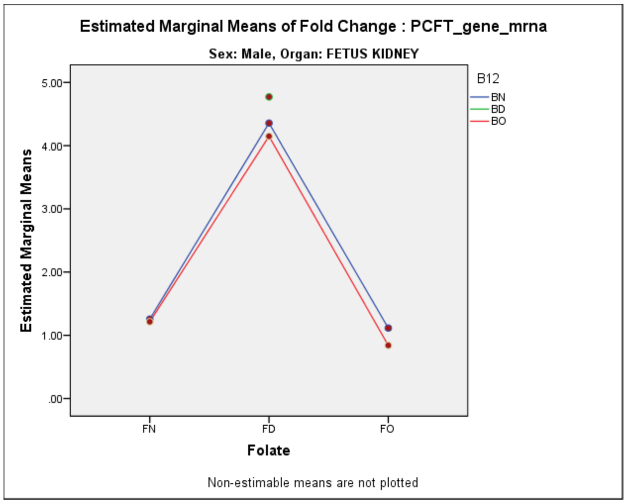

F (i)

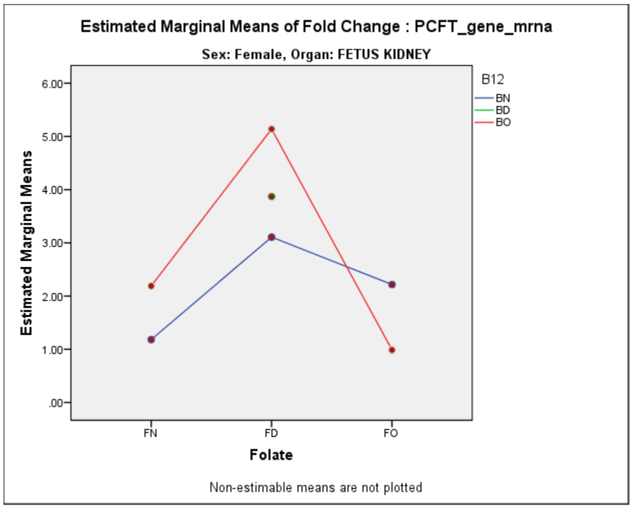

F (ii)

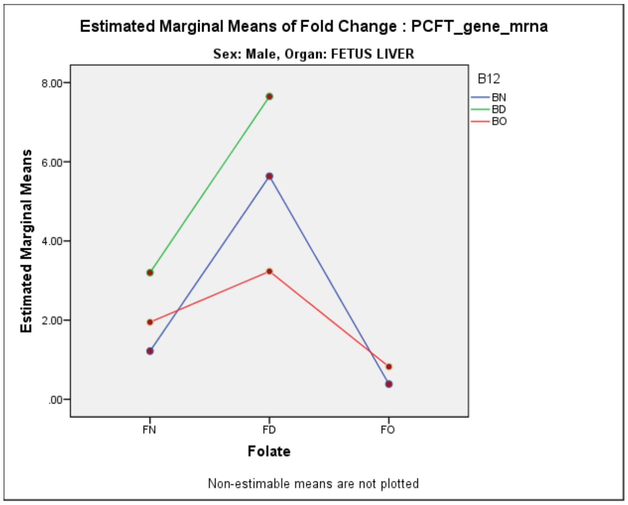

G (i)

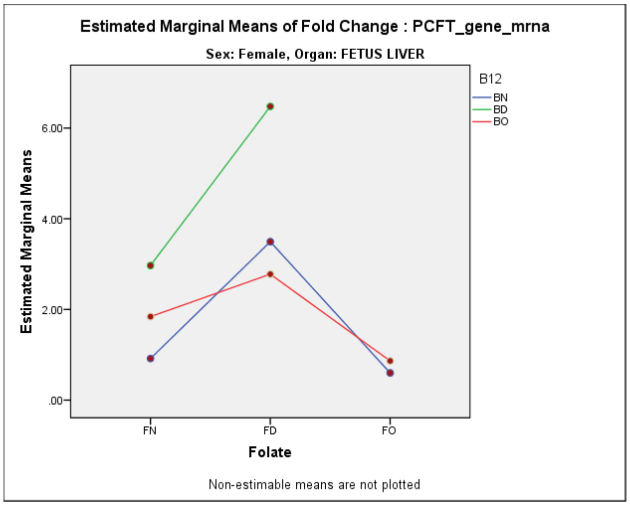

G (ii)

Supplementary figure 4: Profile plots **of mRNA expression of PCFT in maternal tissues (A) Brain (F=2.4, df=4) (B) Kidney (F=48.5, df=4) (C) Liver (F=11.5, df=4) (D) Placenta (F=18.5, df=3), fetal tissues (E) (i) Brain (male) (F=5.2, df=3) (ii) Brain (female) (F=4.1, df=3) (F) (i) Kidney (male) (F=0.05, df=2) (ii) Kidney (female) (F=8.08, df=2) (G) (i) Liver (male) (F=15.2, df=3) (ii) Liver (female) (F=6.0,df=3)**

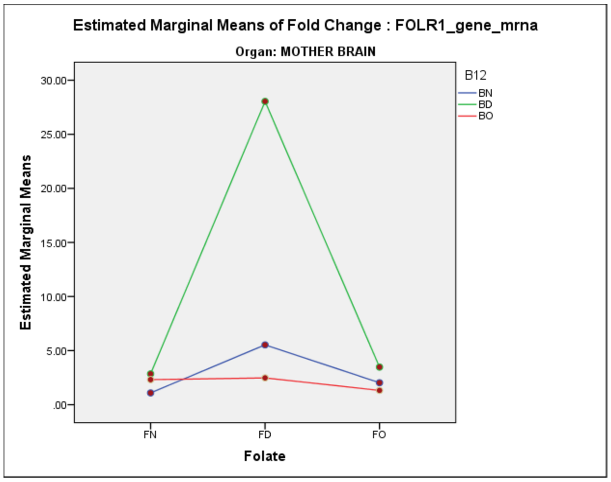
Estimated Marginal Means of Fold Change : FOLR1_gene_mrna
Organ: MOTHER BRAIN
Estimated Marginal Means
30.00
25.00
20.00
15.00
10.00
5.00
.00
FN
FD
FO
Folate
B12
BN
BD
BO

(A)

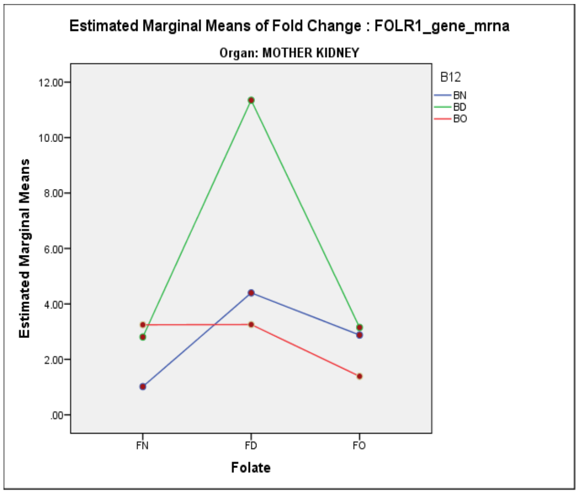
Estimated Marginal Means of Fold Change : FOLR1_gene_mrna
Organ: MOTHER KIDNEY
Estimated Marginal Means
12.00
10.00
8.00
6.00
4.00
2.00
.00
FN
FD
FO
Folate
B12
BN
BD
BO

(B)

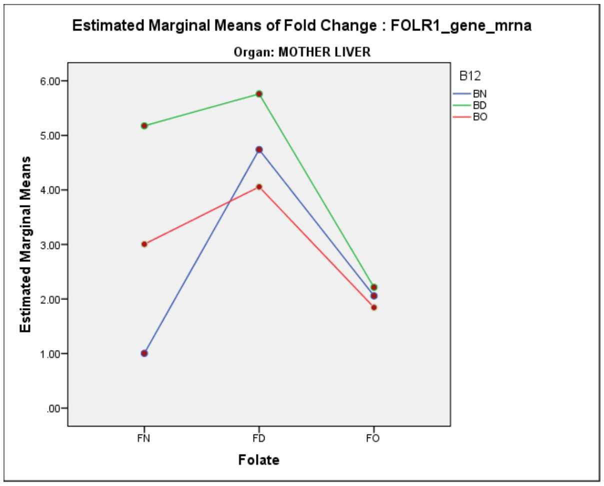
Estimated Marginal Means of Fold Change : FOLR1_gene_mrna
Organ: MOTHER LIVER
Estimated Marginal Means
6.00
5.00
4.00
3.00
2.00
1.00
.00
FN
FD
FO
Folate
B12
BN
BD
BO

(C)

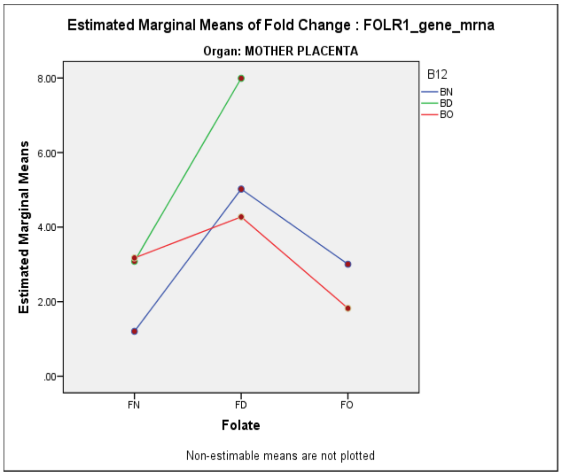
Estimated Marginal Means of Fold Change : FOLR1_gene_mrna
Organ: MOTHER PLACENTA
Estimated Marginal Means
8.00
6.00
4.00
2.00
.00
FN
FD
FO
Folate
Non-estimable means are not plotted
B12
BN
BD
BO

(D)

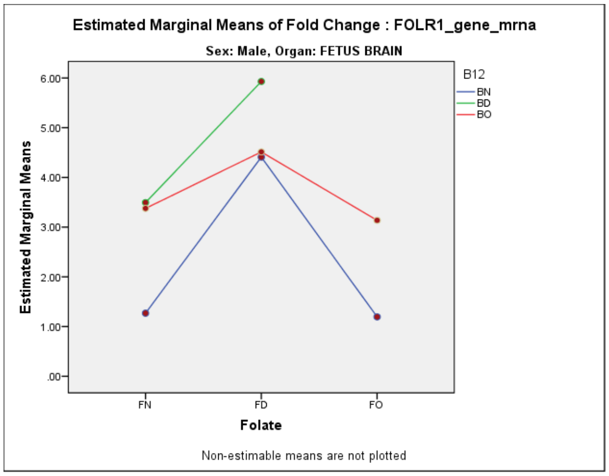
Estimated Marginal Means of Fold Change : FOLR1_gene_mrna
Sex: Male, Organ: FETUS BRAIN
Estimated Marginal Means
6.00
5.00
4.00
3.00
2.00
1.00
.00
FN
FD
FO
Folate
Non-estimable means are not plotted
B12
BN
BD
BO

E (i)

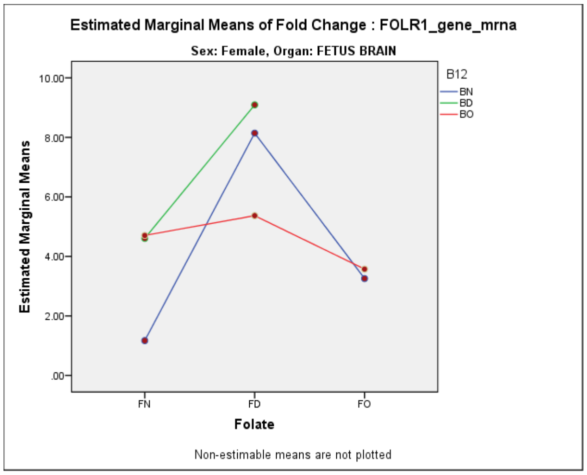
Estimated Marginal Means of Fold Change : FOLR1_gene_mrna
Sex: Female, Organ: FETUS BRAIN
Estimated Marginal Means
10.00
8.00
6.00
4.00
2.00
.00
FN
FD
FO
Folate
Non-estimable means are not plotted
B12
BN
BD
BO

E (ii)

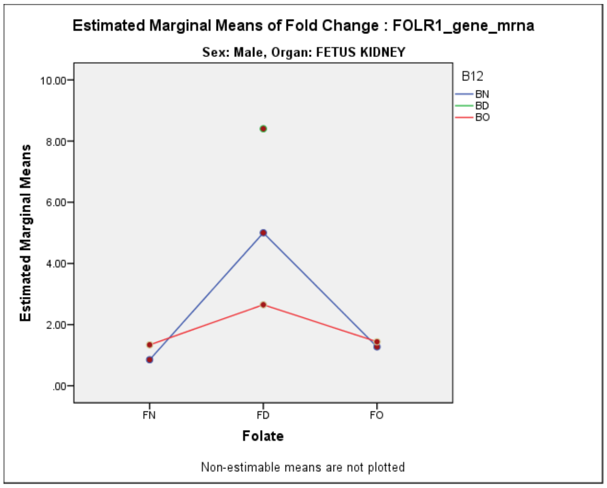

F (i)

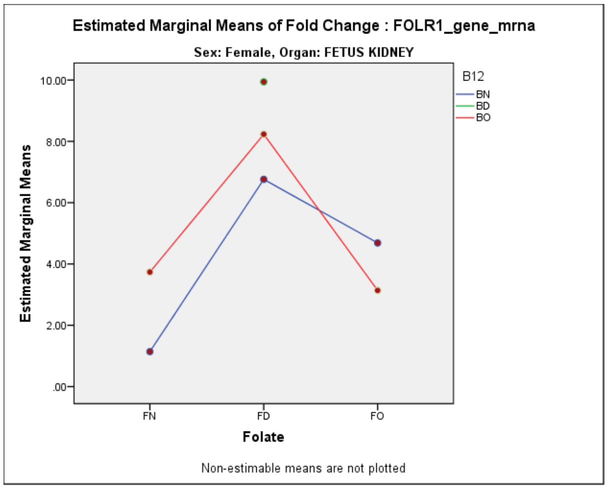

F (ii)

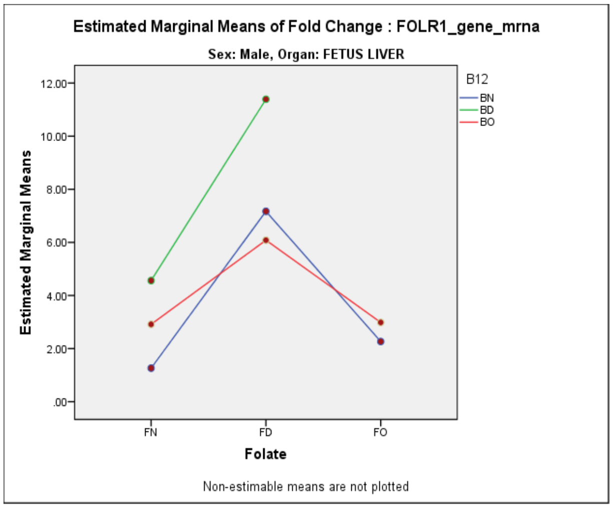

G (i)

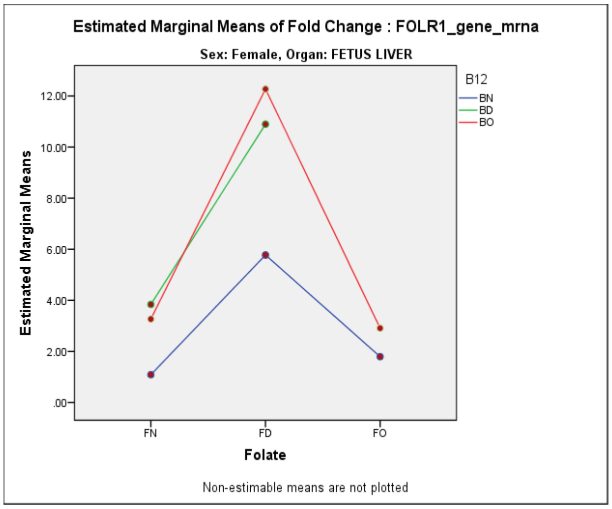

G (ii)

Supplementary figure 5: Profile plots **of mRNA expression of FOLR1 in maternal tissues (A) Brain (F=286.2, df=4) (B) Kidney (F=74.1, df=4) (C) Liver (F=11.3, df=4) (D) Placenta (F=41.1, df=3), fetal tissues (E) (i) Brain (male) (F=1.8, df=3) (ii) Brain (female) (F=10.0, df=3) (F) (i) Kidney (male) (F=7.8, df=2) (ii) Kidney (female) (F=11.2, df=2) (G) (i) Liver (male) (F=5.4, df=3) (ii) Liver (female) (F=9.4,df=3)**

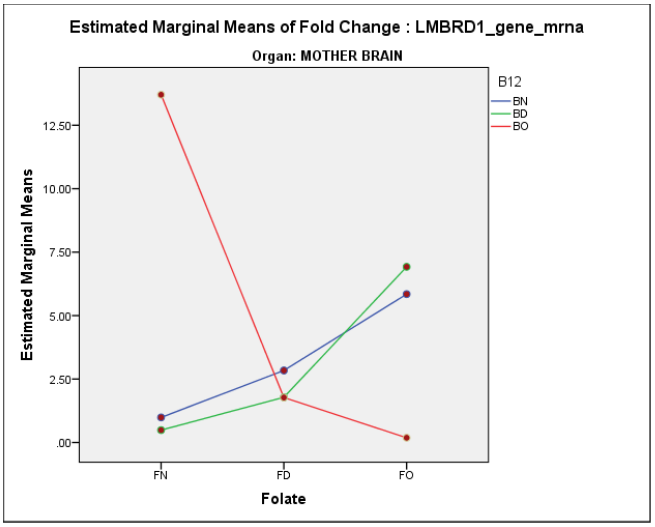
Estimated Marginal Means of Fold Change : LMBRD1_gene_mrna
Organ: MOTHER BRAIN
B12
BN
BD
BO
Estimated Marginal Means
12.50
10.00
7.50
5.00
2.50
.00
FN
FD
FO
Folate

(A)

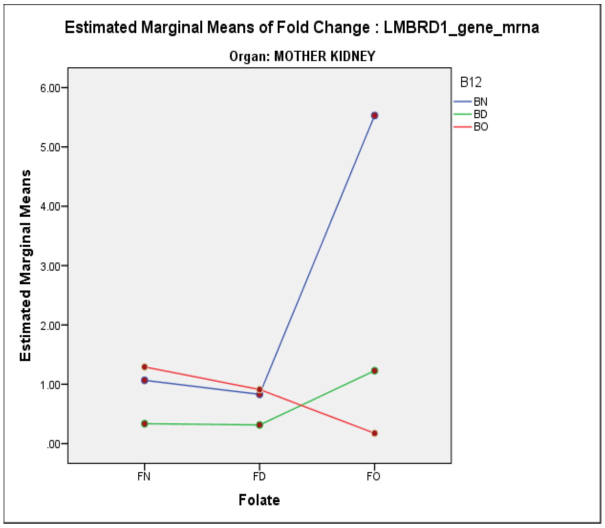
Estimated Marginal Means of Fold Change : LMBRD1_gene_mrna
Organ: MOTHER KIDNEY
B12
BN
BD
BO
Estimated Marginal Means
6.00
5.00
4.00
3.00
2.00
1.00
.00
FN
FD
FO
Folate

(B)

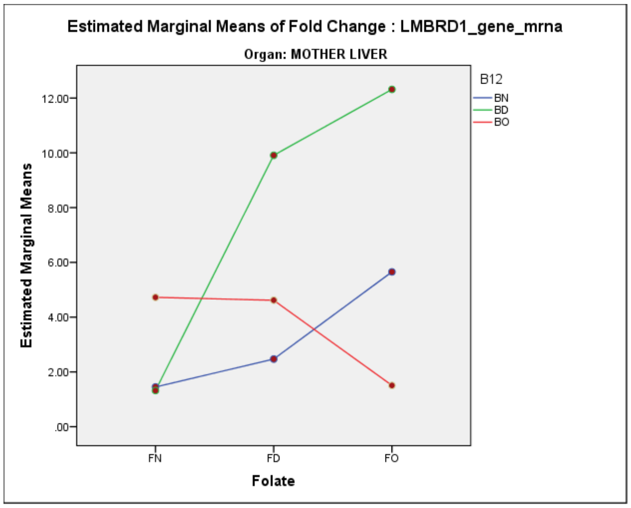
Estimated Marginal Means of Fold Change : LMBRD1_gene_mrna
Organ: MOTHER LIVER
B12
BN
BD
BO
Estimated Marginal Means
12.00
10.00
8.00
6.00
4.00
2.00
.00
FN
FD
FO
Folate

(C)

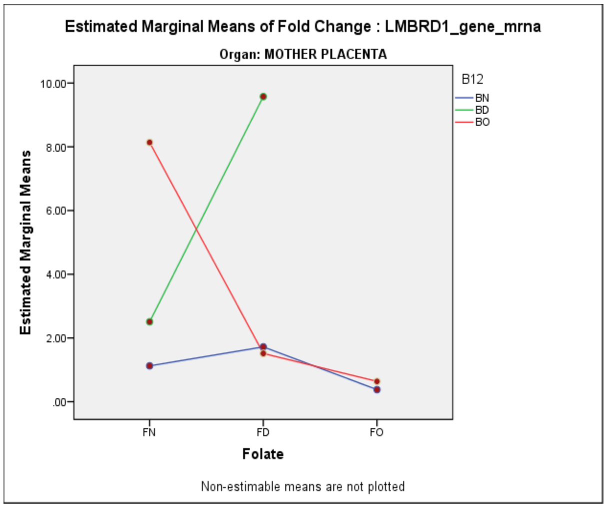
Estimated Marginal Means of Fold Change : LMBRD1_gene_mrna
Organ: MOTHER PLACENTA
B12
BN
BD
BO
Estimated Marginal Means
10.00
8.00
6.00
4.00
2.00
.00
FN
FD
FO
Folate
Non-estimable means are not plotted

(D)

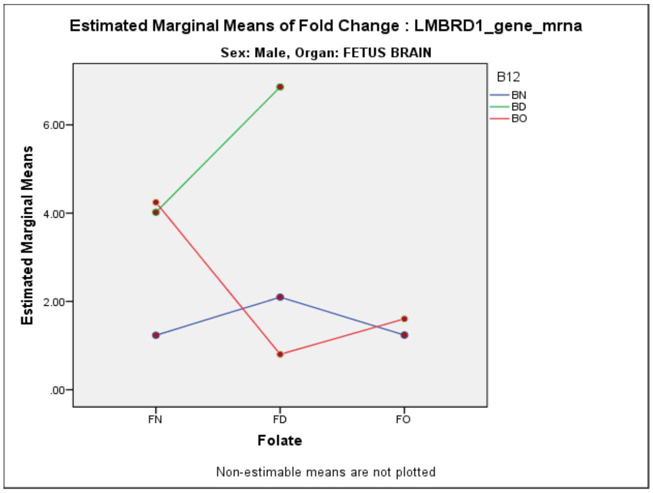
Estimated Marginal Means of Fold Change : LMBRD1_gene_mrna
Sex: Male, Organ: FETUS BRAIN
B12
BN
BD
BO
Estimated Marginal Means
6.00
4.00
2.00
.00
FN
FD
FO
Folate
Non-estimable means are not plotted

E (i)

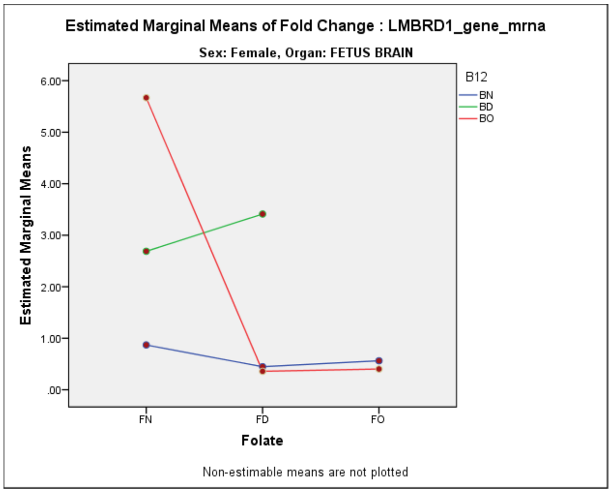
Estimated Marginal Means of Fold Change : LMBRD1_gene_mrna
Sex: Female, Organ: FETUS BRAIN
B12
BN
BD
BO
Estimated Marginal Means
6.00
5.00
4.00
3.00
2.00
1.00
.00
FN
FD
FO
Folate
Non-estimable means are not plotted

E (ii)

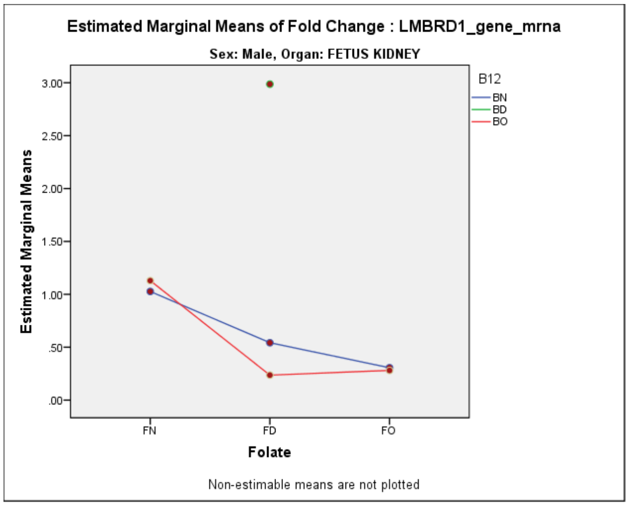

F (i)

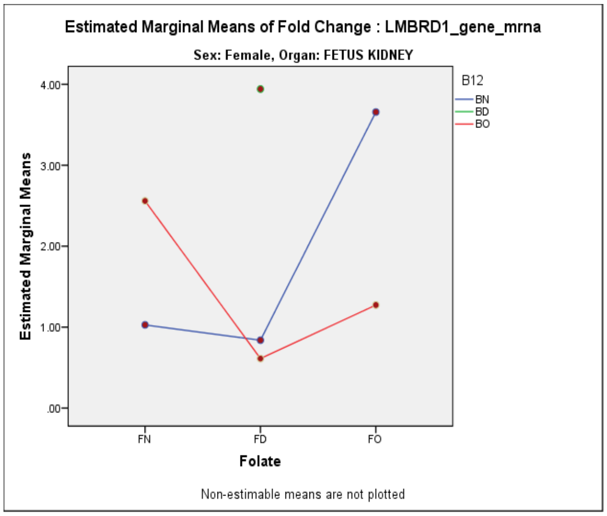

F (ii)

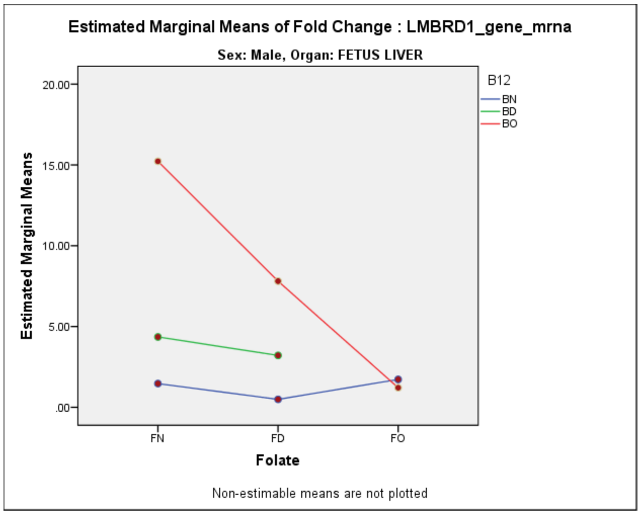

G (i)

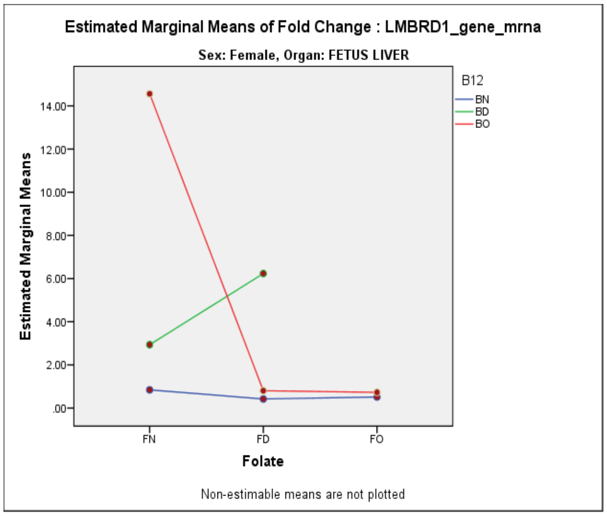

G (ii)

Supplementary figure 6: Profile plots **of mRNA expression of LMBRD1 in maternal tissues (A) Brain (F=1056.8, df=4) (B) Kidney (F=83.6, df=4) (C) Liver (F=24.0, df=4) (D) Placenta (F=150.2, df=3), fetal tissues (E) (i) Brain (male) (F=15.5, df=3) (ii) Brain (female) (F=35.4, df=3) (F) (i) Kidney (male) (F=0.82, df=2) (ii) Kidney (female) (F=23.9, df=2) (G) (i) Liver (male) (F=132.9, df=3) (ii) Liver (female) (F=272.4,df=3)**

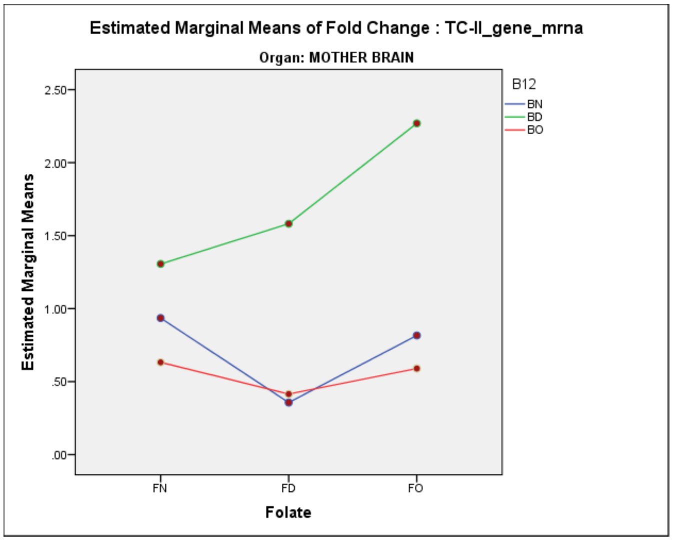
Estimated Marginal Means of Fold Change : TC-II_gene_mrna
Organ: MOTHER BRAIN
Estimated Marginal Means
2.50
2.00
1.50
1.00
.50
.00
B12
BN
BD
BO
FN
FD
FO
Folate

(A)

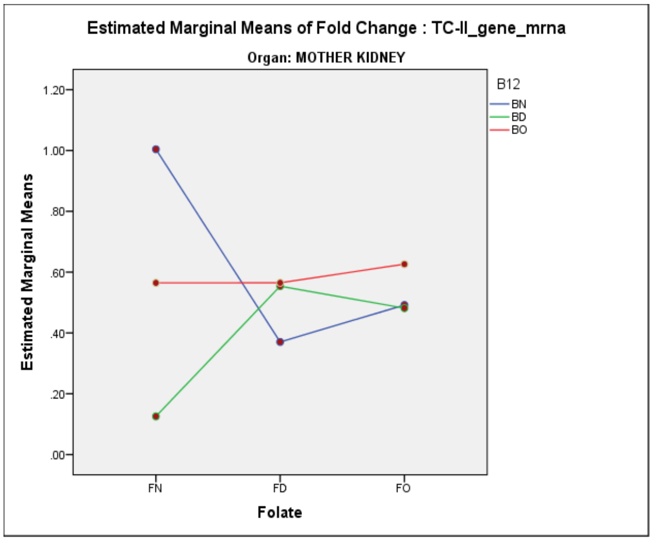
Estimated Marginal Means of Fold Change : TC-II_gene_mrna
Organ: MOTHER KIDNEY
Estimated Marginal Means
1.20
1.00
.80
.60
.40
.20
.00
B12
BN
BD
BO
FN
FD
FO
Folate

(B)

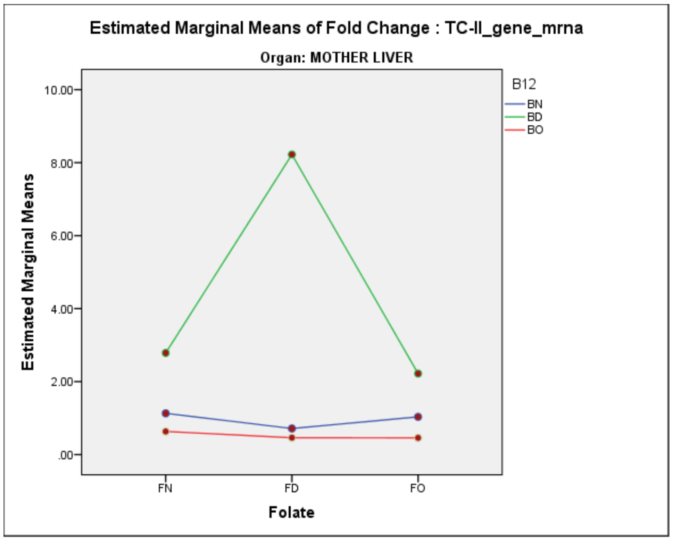
Estimated Marginal Means of Fold Change : TC-II_gene_mrna
Organ: MOTHER LIVER
Estimated Marginal Means
10.00
8.00
6.00
4.00
2.00
.00
B12
BN
BD
BO
FN
FD
FO
Folate

(C)

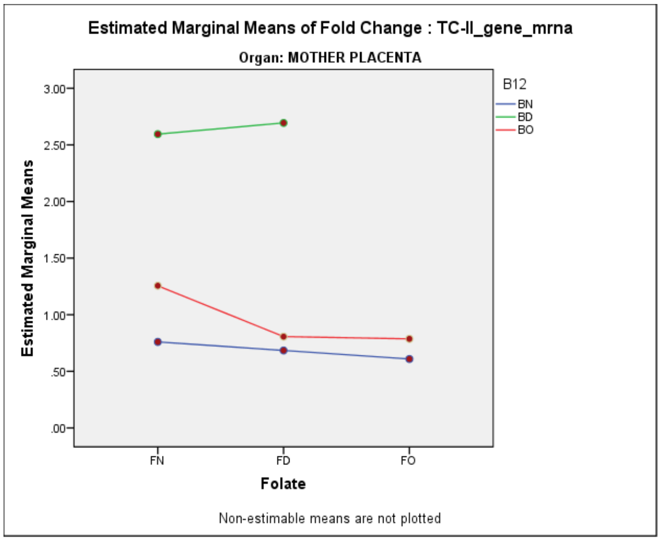
Estimated Marginal Means of Fold Change : TC-II_gene_mrna
Organ: MOTHER PLACENTA
Estimated Marginal Means
3.00
2.50
2.00
1.50
1.00
.50
.00
B12
BN
BD
BO
FN
FD
FO
Folate
Non-estimable means are not plotted

(D)

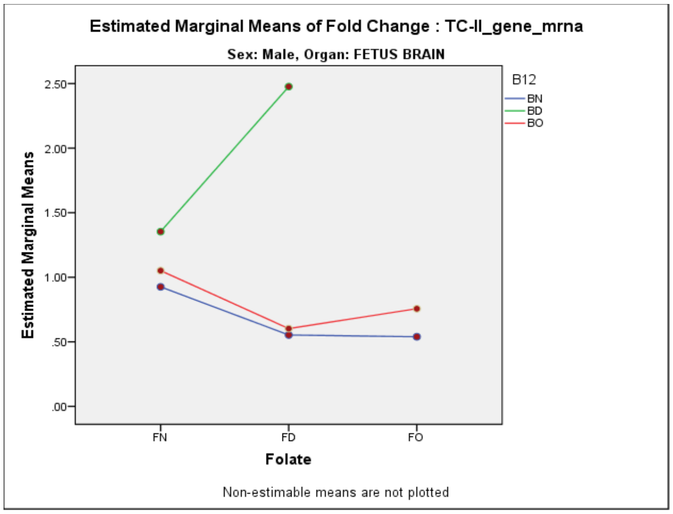
Estimated Marginal Means of Fold Change : TC-II_gene_mrna
Sex: Male, Organ: FETUS BRAIN
Estimated Marginal Means
2.50
2.00
1.50
1.00
.50
.00
B12
BN
BD
BO
FN
FD
FO
Folate
Non-estimable means are not plotted

E (i)

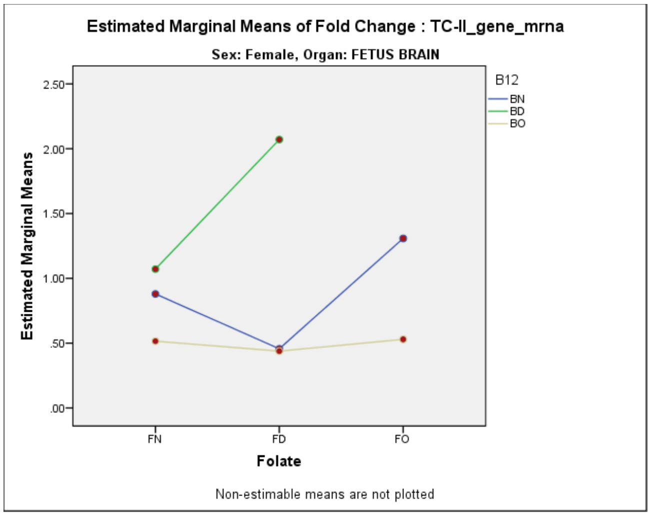
Estimated Marginal Means of Fold Change : TC-II_gene_mrna
Sex: Female, Organ: FETUS BRAIN
Estimated Marginal Means
2.50
2.00
1.50
1.00
.50
.00
B12
BN
BD
BO
FN
FD
FO
Folate
Non-estimable means are not plotted

E (ii)

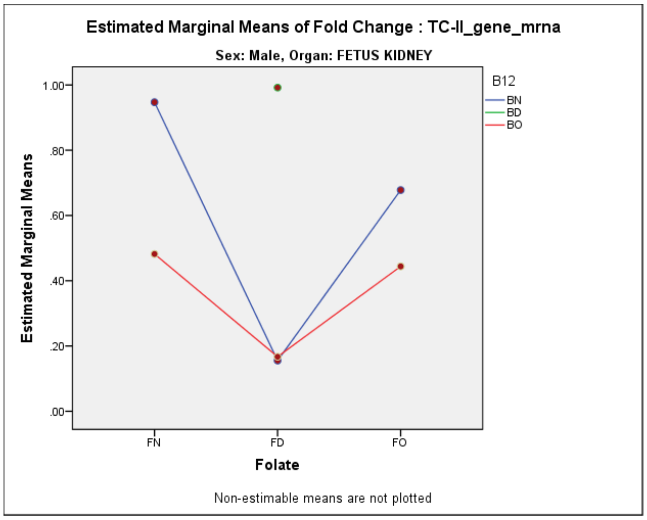

F (i)

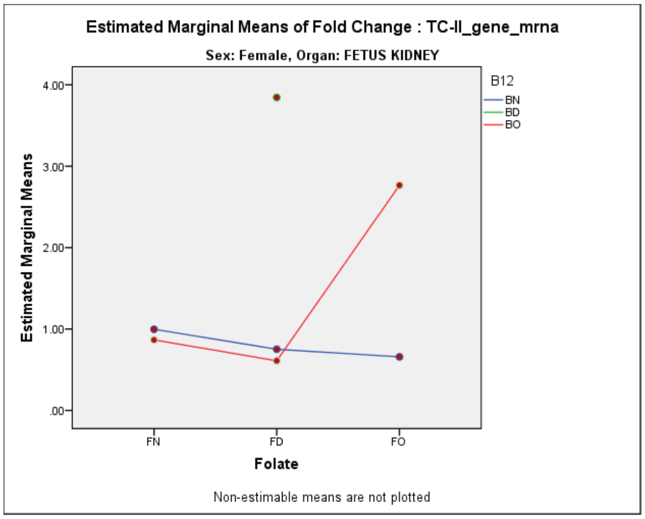

F (ii)

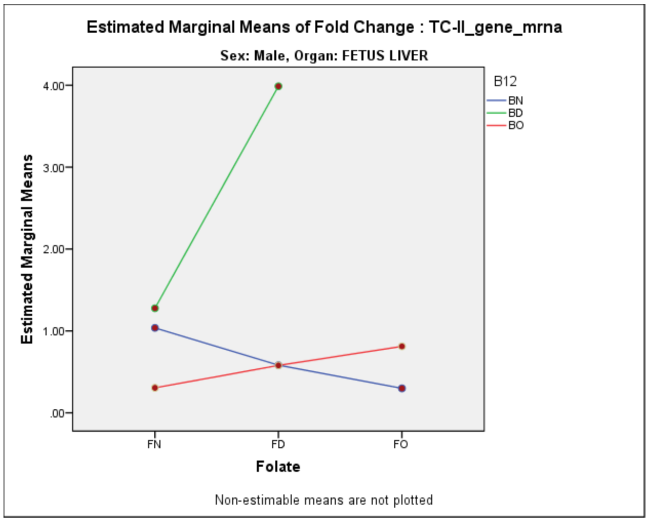

G (i)

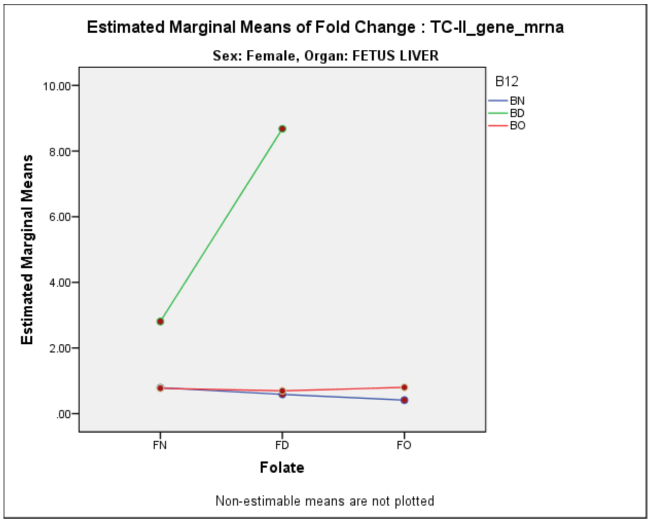

G (ii)

Supplementary figure 7: Profile plots **of mRNA expression of TC-II in maternal tissues (A) Brain (F=3.7, df=4) (B) Kidney (F=12.4, df=4) (C) Liver (F=66.6, df=4) (D) Placenta (F=1.76, df=3), fetal tissues (E) (i) Brain (male) (F=18.8, df=3) (ii) Brain (female) (F=14.0, df=3) (F) (i) Kidney (male) (F=5.1, df=2) (ii) Kidney (female) (F=8.5, df=2) (G) (i) Liver (male) (F=27.5, df=3) (ii) Liver (female) (F=50.6,df=3)**

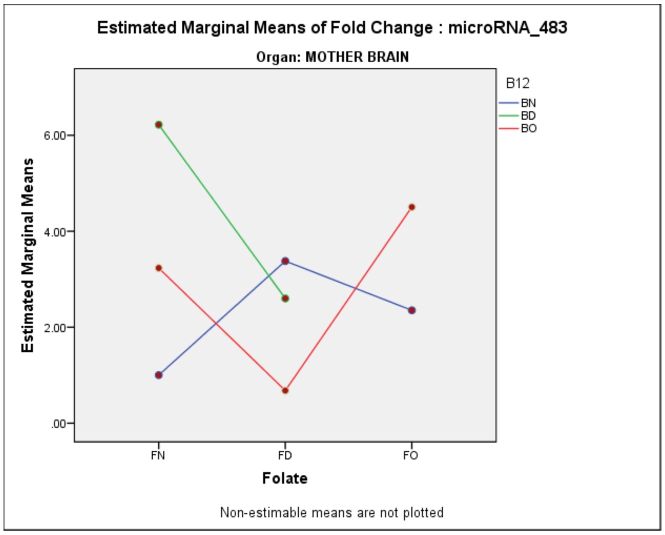

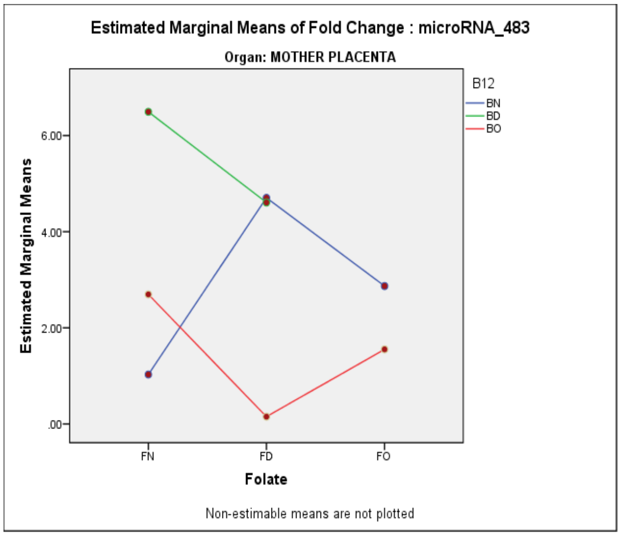

(A)

(B)

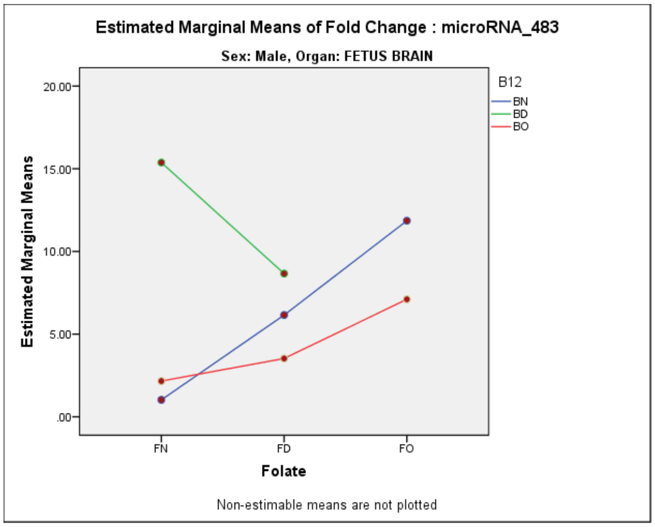

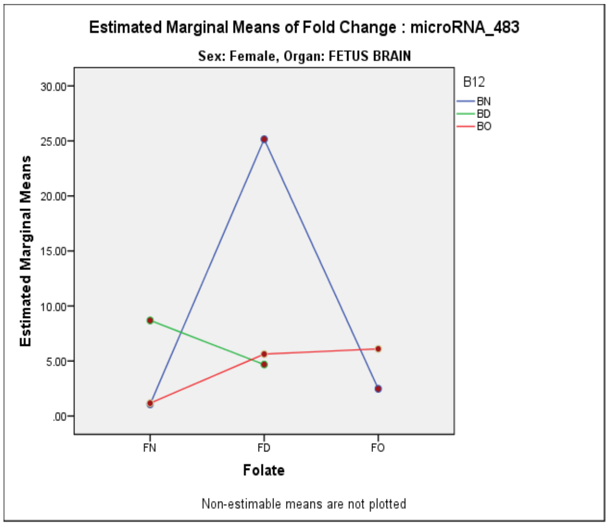

C (i)

C (ii)

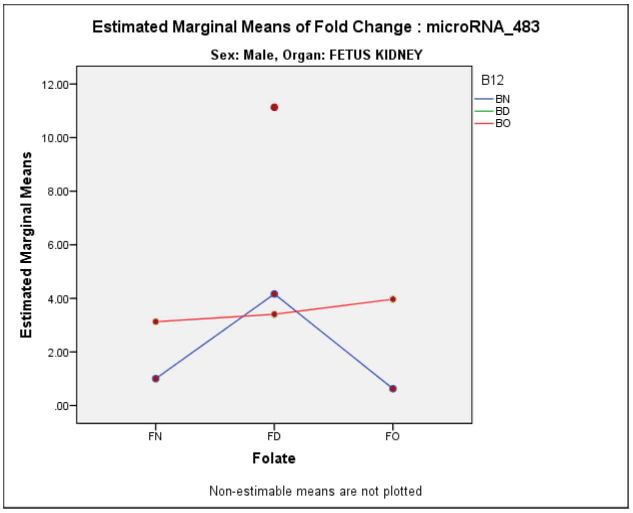

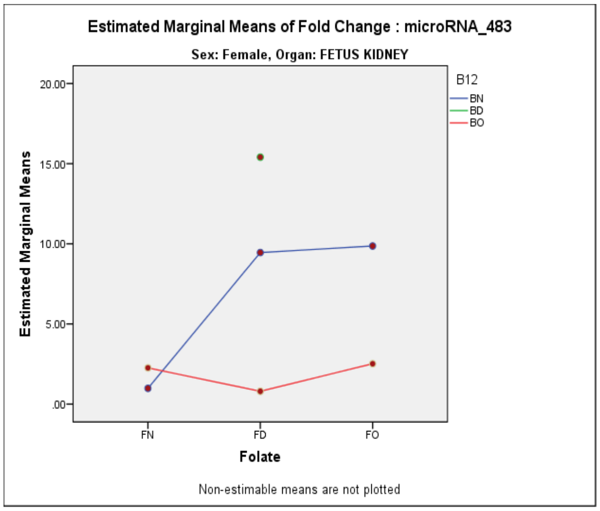

D (i)

D (ii)

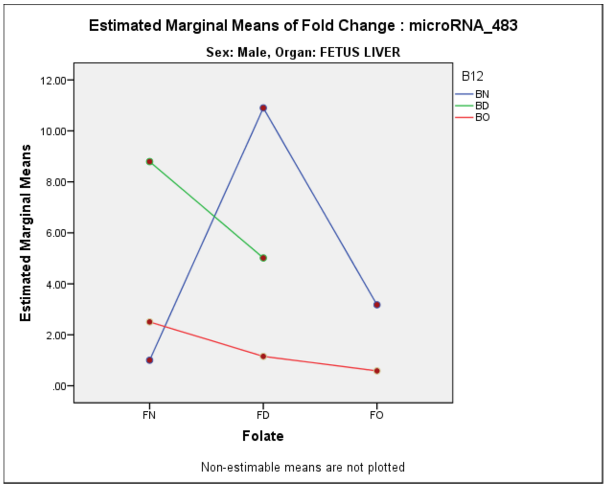

E (i)

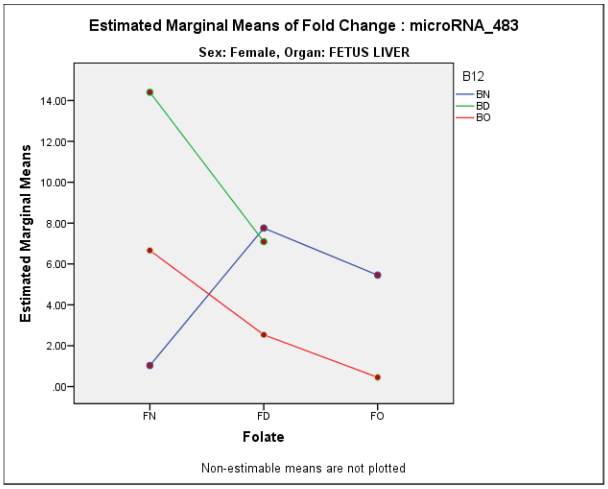

E (ii)

Supplementary figure 8: Profile plots **of mRNA expression of miR483 in maternal tissues (A) Brain (F=92.3, df=3) (B) Placenta (F=152.3, df=3), fetal tissues (C) (i) Brain (male) (F=55.8, df=3) (ii) Brain (female) (F=353.3, df=3) (D) (i) Kidney (male) (F=14.7, df=2) (ii) Kidney (female) (F=101.6, df=2) (E) (i) Liver (male) (F=115.8, df=3) (ii) Liver (female) (F=97.1,df=3)**

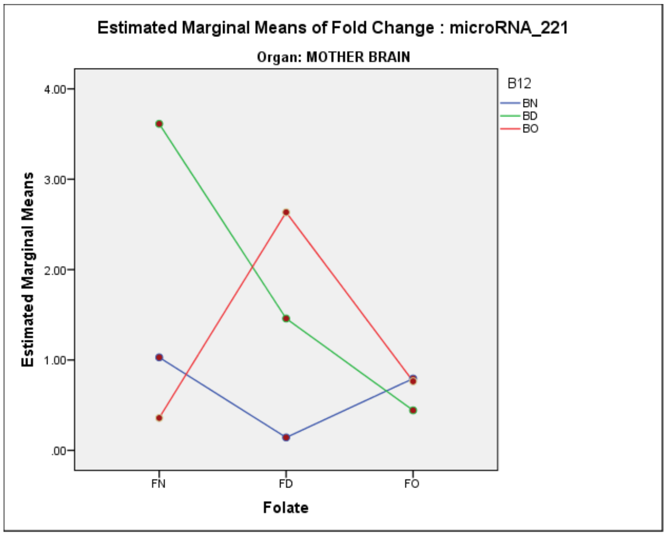
Estimated Marginal Means of Fold Change : microRNA_221
Organ: MOTHER BRAIN
Estimated Marginal Means
4.00
3.00
2.00
1.00
.00
FN
FD
FO
Folate
B12
BN
BD
BO

(A)

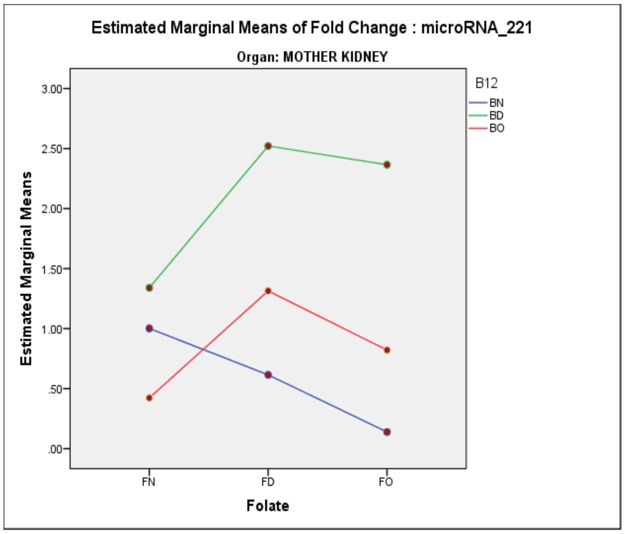
Estimated Marginal Means of Fold Change : microRNA_221
Organ: MOTHER KIDNEY
Estimated Marginal Means
3.00
2.50
2.00
1.50
1.00
.50
.00
FN
FD
FO
Folate
B12
BN
BD
BO

(B)

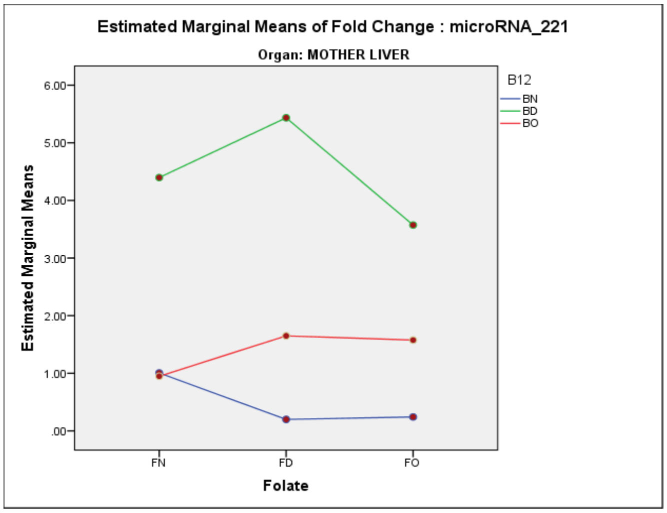
Estimated Marginal Means of Fold Change : microRNA_221
Organ: MOTHER LIVER
Estimated Marginal Means
6.00
5.00
4.00
3.00
2.00
1.00
.00
FN
FD
FO
Folate
B12
BN
BD
BO

(C)

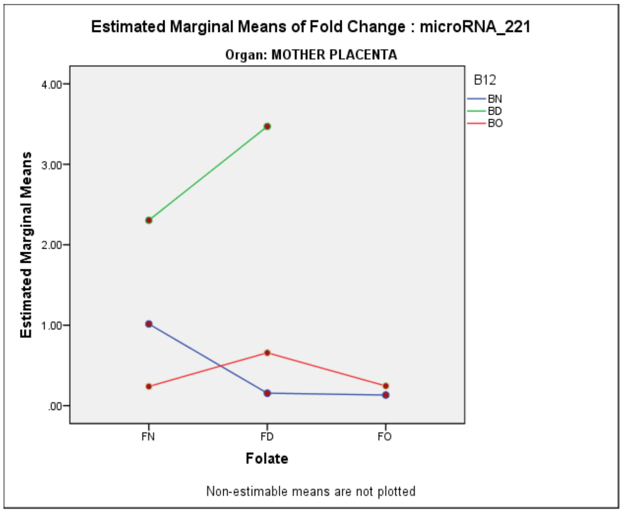
Estimated Marginal Means of Fold Change : microRNA_221
Organ: MOTHER PLACENTA
Estimated Marginal Means
4.00
3.00
2.00
1.00
.00
FN
FD
FO
Folate
B12
BN
BD
BO
Non-estimable means are not plotted

(D)

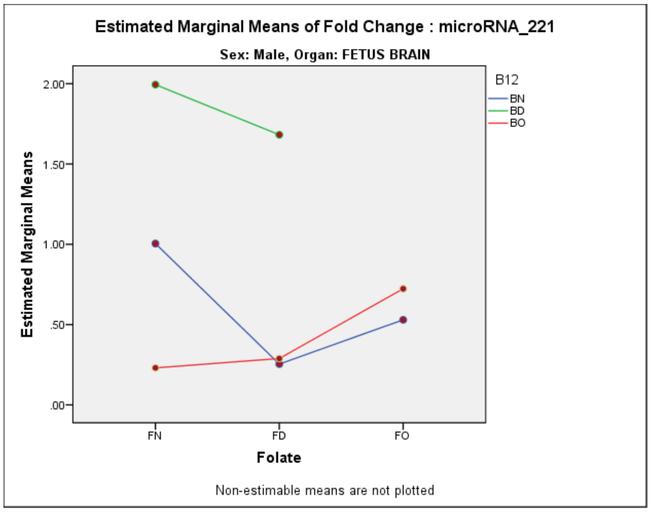
Estimated Marginal Means of Fold Change : microRNA_221
Sex: Male, Organ: FETUS BRAIN
Estimated Marginal Means
2.00
1.50
1.00
.50
.00
FN
FD
FO
Folate
B12
BN
BD
BO
Non-estimable means are not plotted

E (i)

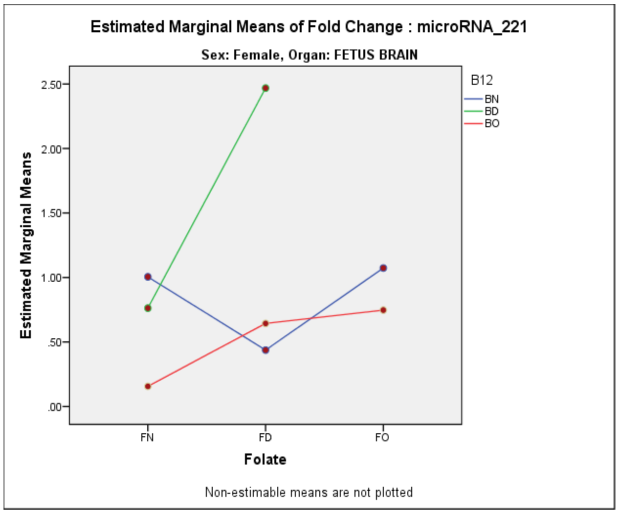
Estimated Marginal Means of Fold Change : microRNA_221
Sex: Female, Organ: FETUS BRAIN
Estimated Marginal Means
2.50
2.00
1.50
1.00
.50
.00
FN
FD
FO
Folate
B12
BN
BD
BO
Non-estimable means are not plotted

E (ii)

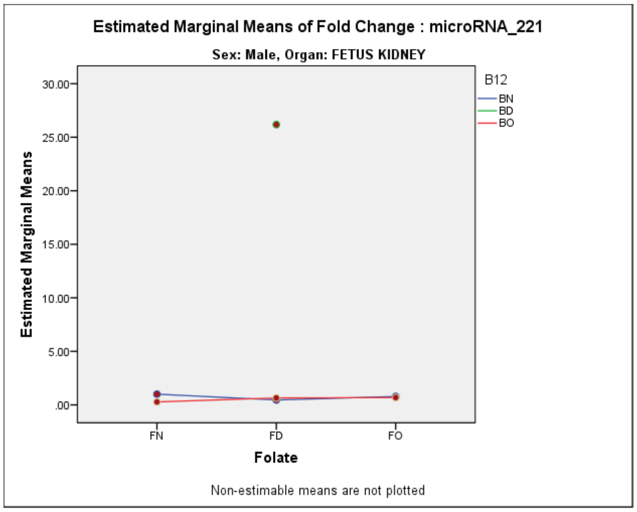

F (i)

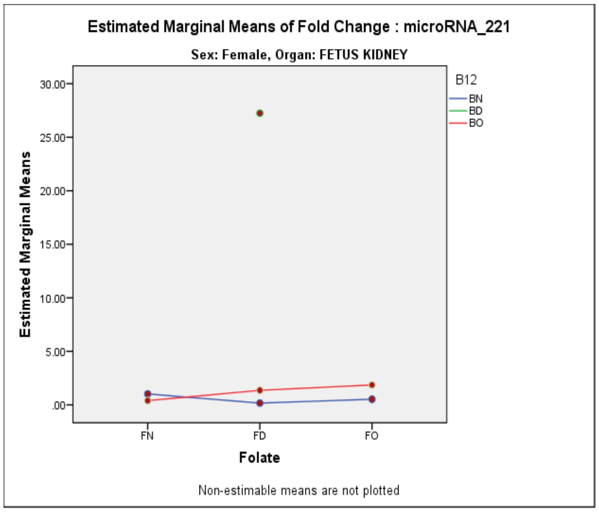

F (ii)

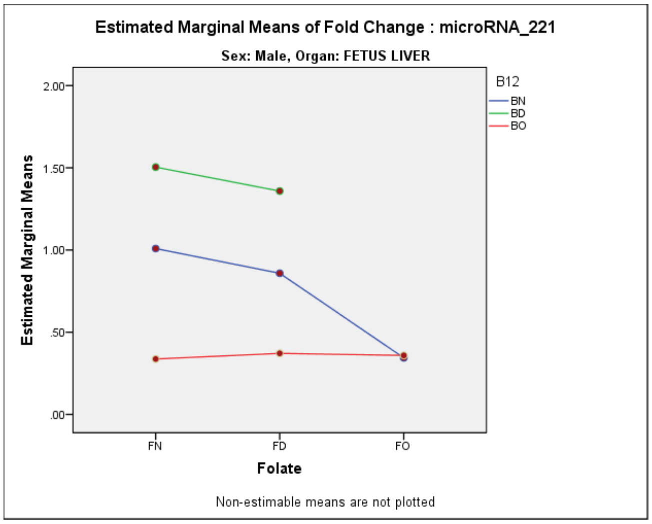

G (i)

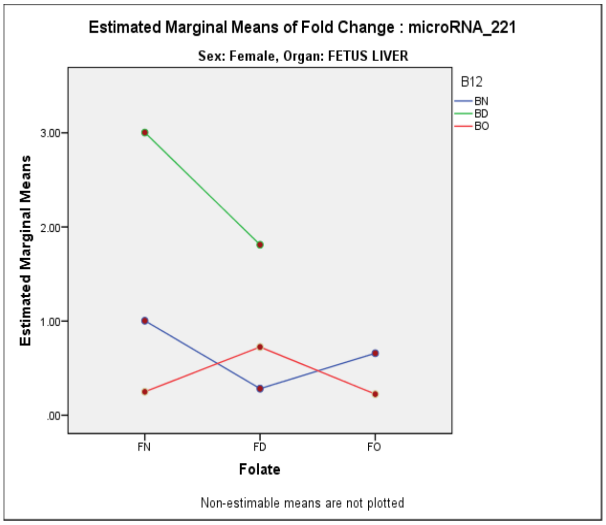

G (ii)

Supplementary figure 9: Profile plots **of mRNA expression of miR221 in maternal tissues (A) Brain (F=139.6, df=4) (B) Kidney (F=6.9, df=4) (C) Liver (F=37.6, df=4) (D) Placenta (F=75.3, df=3), fetal tissues (E) (i) Brain (male) (F=6.6, df=3) (ii) Brain (female) (F=17.5, df=3) (F) (i) Kidney (male) (F=1.7, df=2) (ii) Kidney (female) (F=16.4, df=2) (G) (i) Liver (male) (F=5.7, df=3) (ii) Liver (female) (F=167.0,df=3)**

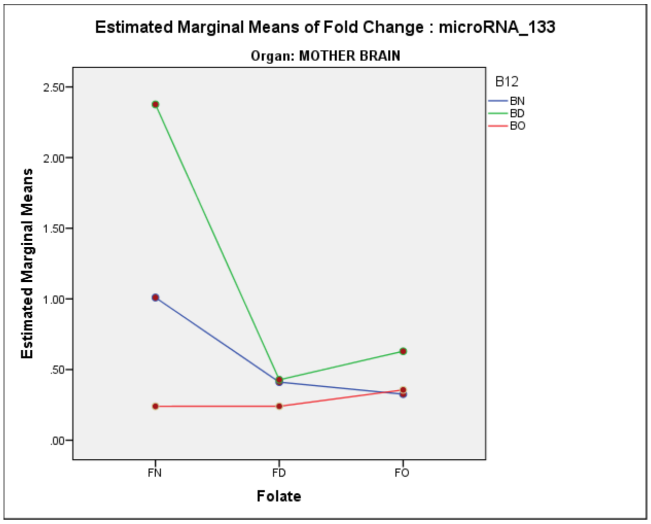
Estimated Marginal Means of Fold Change : microRNA_133
Organ: MOTHER BRAIN
Estimated Marginal Means
2.50
2.00
1.50
1.00
.50
.00
FN
FD
FO
Folate
B12
BN
BD
BO

(A)

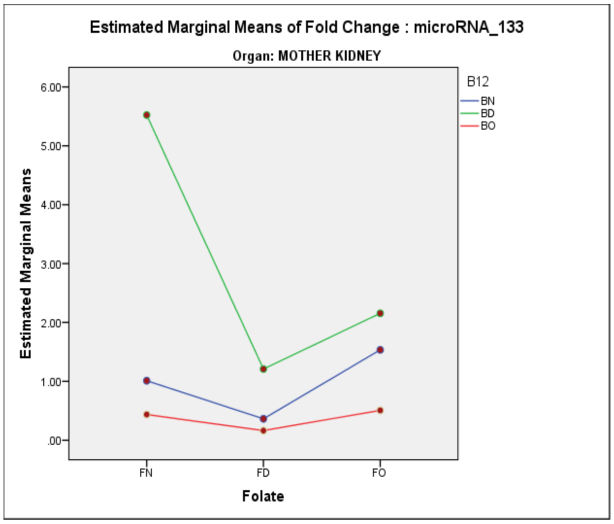
Estimated Marginal Means of Fold Change : microRNA_133
Organ: MOTHER KIDNEY
Estimated Marginal Means
6.00
5.00
4.00
3.00
2.00
1.00
.00
FN
FD
FO
Folate
B12
BN
BD
BO

(B)

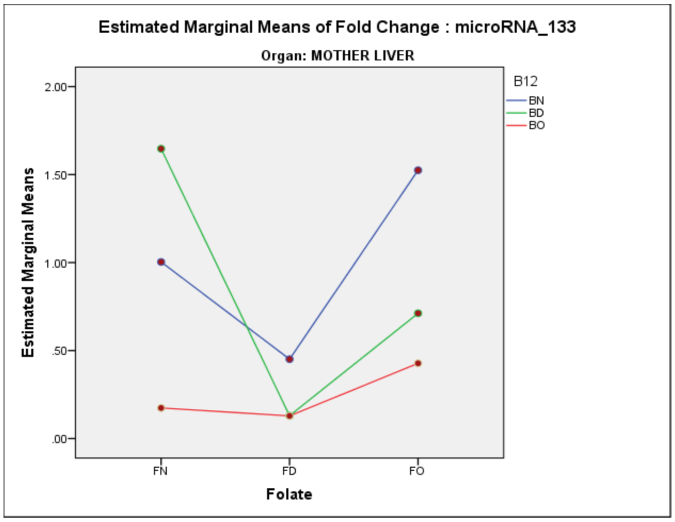
Estimated Marginal Means of Fold Change : microRNA_133
Organ: MOTHER LIVER
Estimated Marginal Means
2.00
1.50
1.00
.50
.00
FN
FD
FO
Folate
B12
BN
BD
BO

(C)

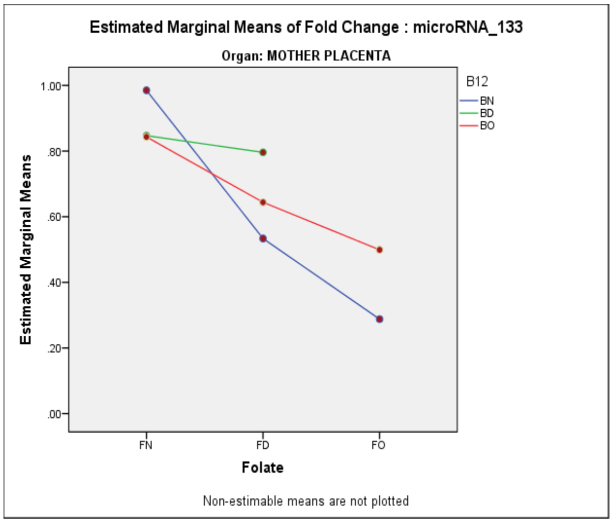
Estimated Marginal Means of Fold Change : microRNA_133
Organ: MOTHER PLACENTA
Estimated Marginal Means
1.00
.80
.60
.40
.20
.00
FN
FD
FO
Folate
B12
BN
BD
BO
Non-estimable means are not plotted

(D)

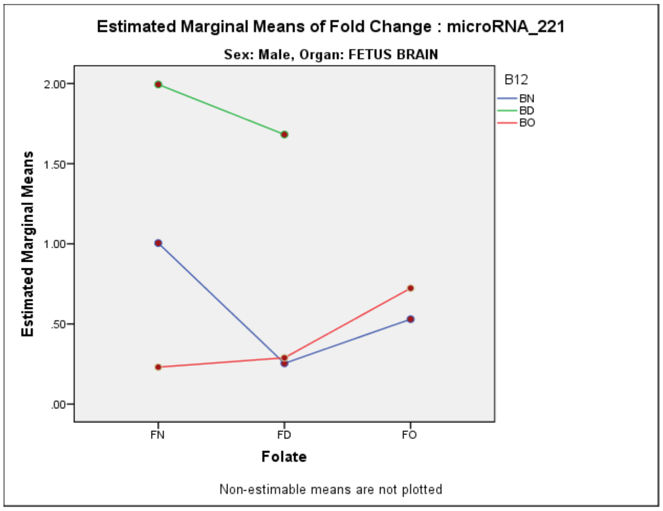
Estimated Marginal Means of Fold Change : microRNA_221
Sex: Male, Organ: FETUS BRAIN
Estimated Marginal Means
2.00
1.50
1.00
.50
.00
FN
FD
FO
Folate
B12
BN
BD
BO
Non-estimable means are not plotted

E (i)

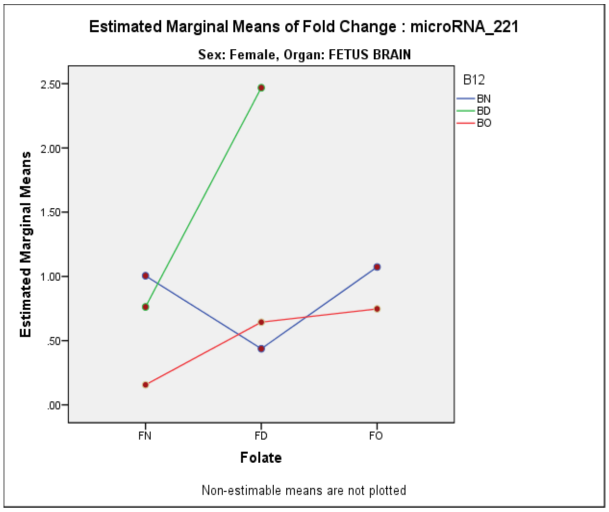
Estimated Marginal Means of Fold Change : microRNA_221
Sex: Female, Organ: FETUS BRAIN
Estimated Marginal Means
2.50
2.00
1.50
1.00
.50
.00
FN
FD
FO
Folate
B12
BN
BD
BO
Non-estimable means are not plotted

E (ii)

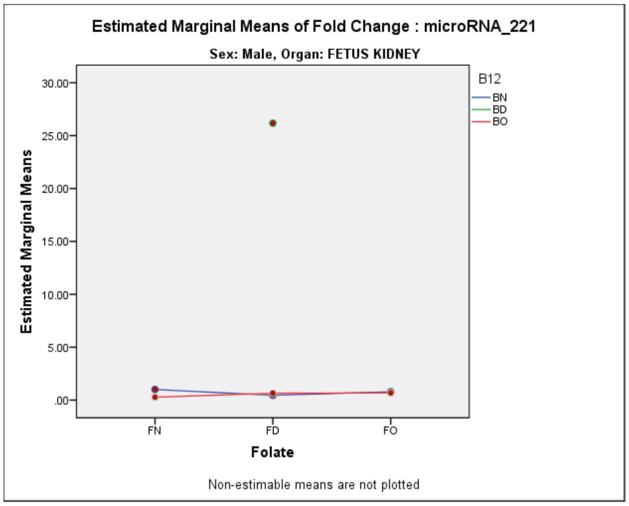

F (i)

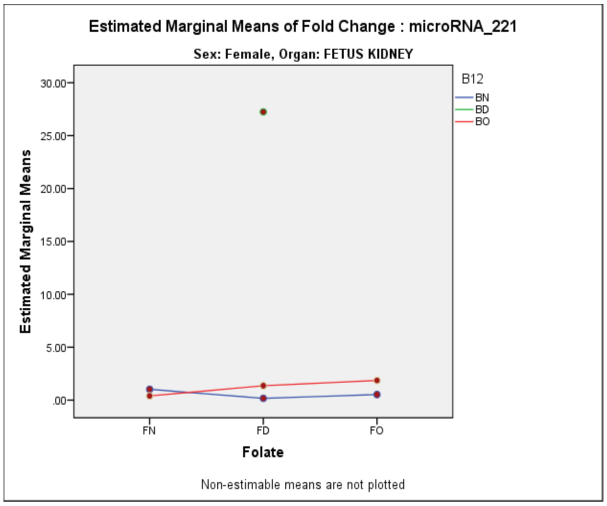

F (ii)

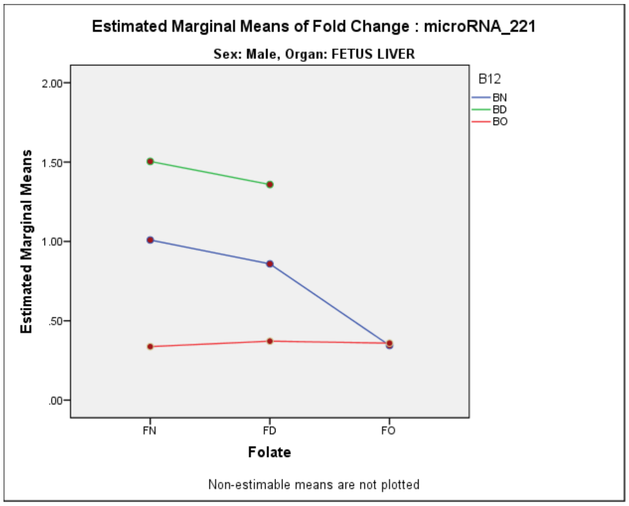

G (i)

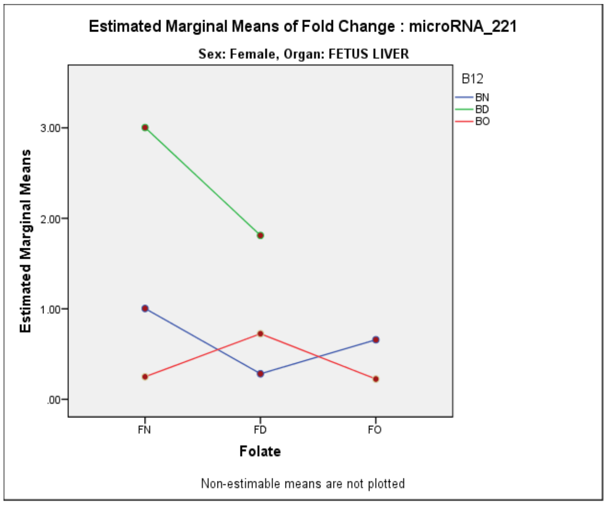

G (ii)

Supplementary figure 10: Profile plots **of mRNA expression of miR133 in maternal tissues (A) Brain (F=38.7, df=4) (B) Kidney (F=68.4, df=4) (C) Liver (F=24.0, df=4) (D) Placenta (F=5.3, df=3), fetal tissues (E) (i) Brain (male) (F=6.6, df=3) (ii) Brain (female) (F=17.5, df=3) (F) (i) Kidney (male) (F=1.71, df=2) (ii) Kidney (female) (F=16.4, df=2) (G) (i) Liver (male) (F=5.7, df=3) (ii) Liver (female) (F=167.0,df=3)**

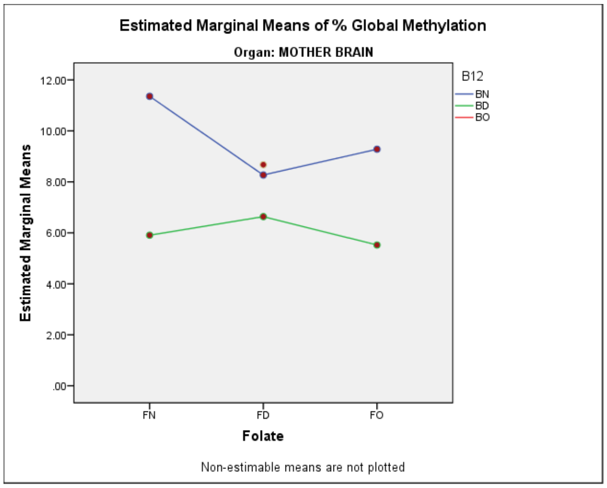
Estimated Marginal Means of % Global Methylation
Organ: MOTHER BRAIN
Estimated Marginal Means
12.00
10.00
8.00
6.00
4.00
2.00
.00
FN
FD
FO
Folate
B12
BN
BD
BO
Non-estimable means are not plotted

(A)

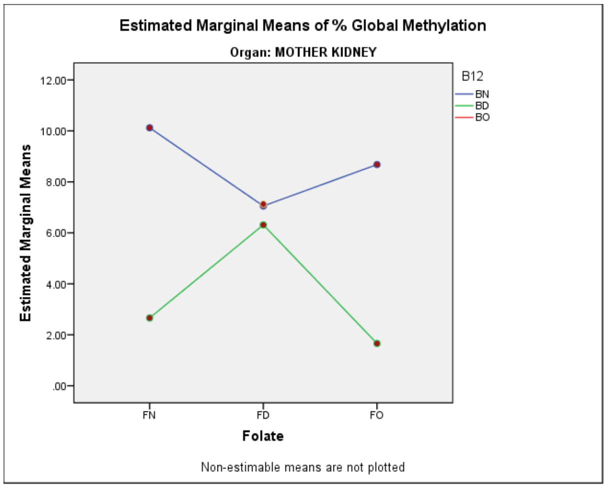
Estimated Marginal Means of % Global Methylation
Organ: MOTHER KIDNEY
Estimated Marginal Means
12.00
10.00
8.00
6.00
4.00
2.00
.00
FN
FD
FO
Folate
B12
BN
BD
BO
Non-estimable means are not plotted

(B)

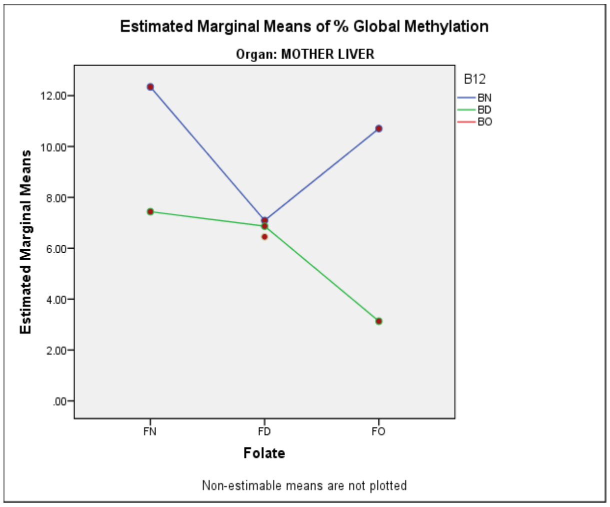
Estimated Marginal Means of % Global Methylation
Organ: MOTHER LIVER
Estimated Marginal Means
12.00
10.00
8.00
6.00
4.00
2.00
.00
FN
FD
FO
Folate
B12
BN
BD
BO
Non-estimable means are not plotted

(C)

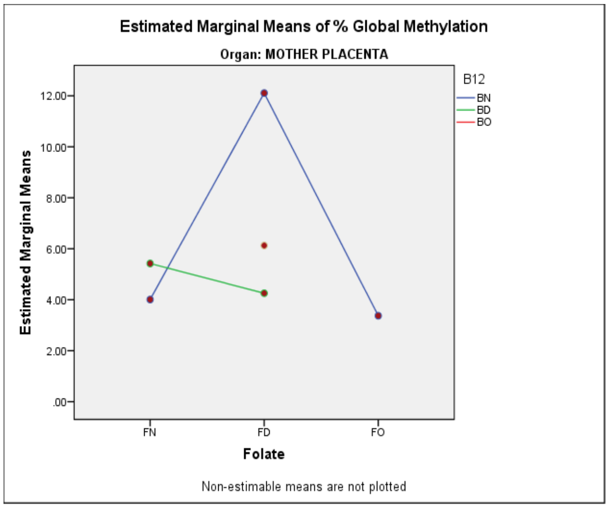
Estimated Marginal Means of % Global Methylation
Organ: MOTHER PLACENTA
Estimated Marginal Means
12.00
10.00
8.00
6.00
4.00
2.00
.00
FN
FD
FO
Folate
B12
BN
BD
BO
Non-estimable means are not plotted

(D)

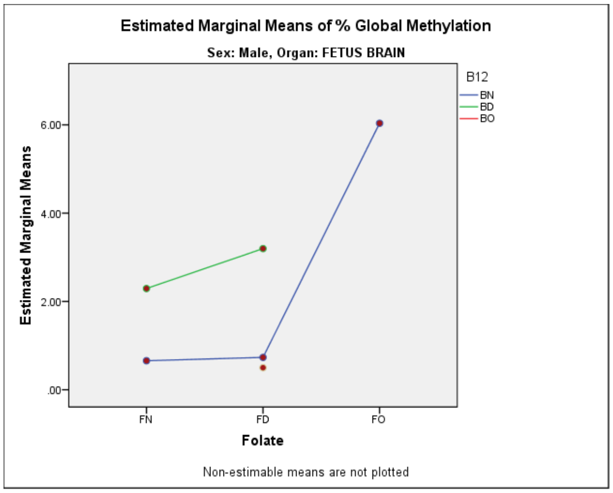
Estimated Marginal Means of % Global Methylation
Sex: Male, Organ: FETUS BRAIN
Estimated Marginal Means
6.00
4.00
2.00
.00
FN
FD
FO
Folate
B12
BN
BD
BO
Non-estimable means are not plotted

E (i)

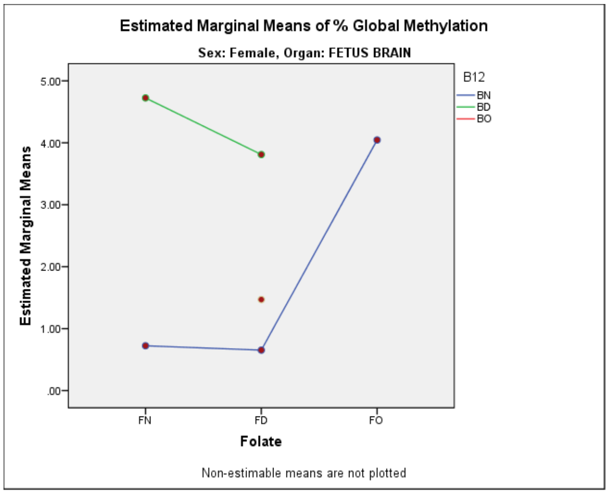
Estimated Marginal Means of % Global Methylation
Sex: Female, Organ: FETUS BRAIN
Estimated Marginal Means
5.00
4.00
3.00
2.00
1.00
.00
FN
FD
FO
Folate
B12
BN
BD
BO
Non-estimable means are not plotted

E (ii)

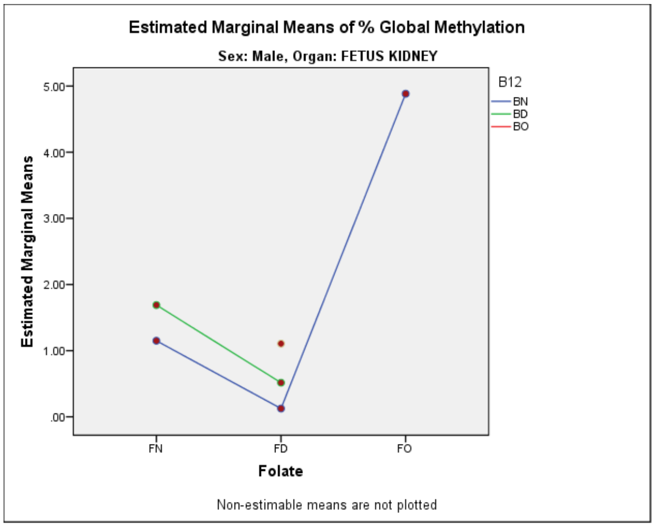

F (i)

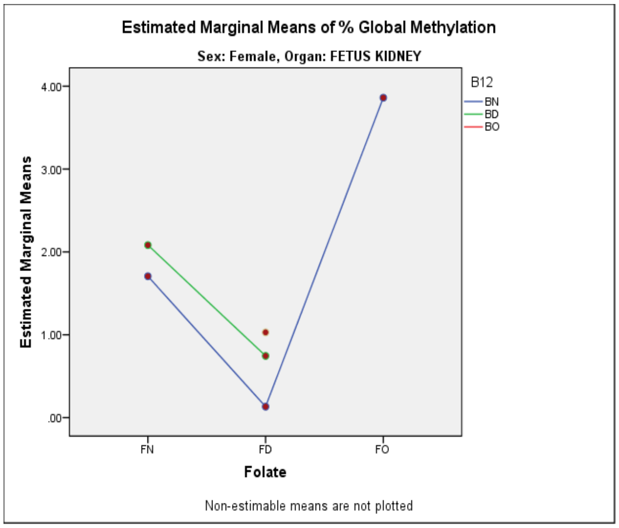

F (ii)

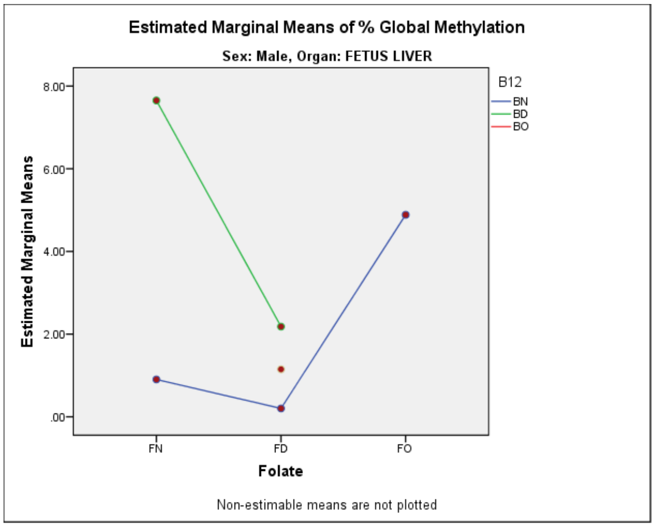

G (i)

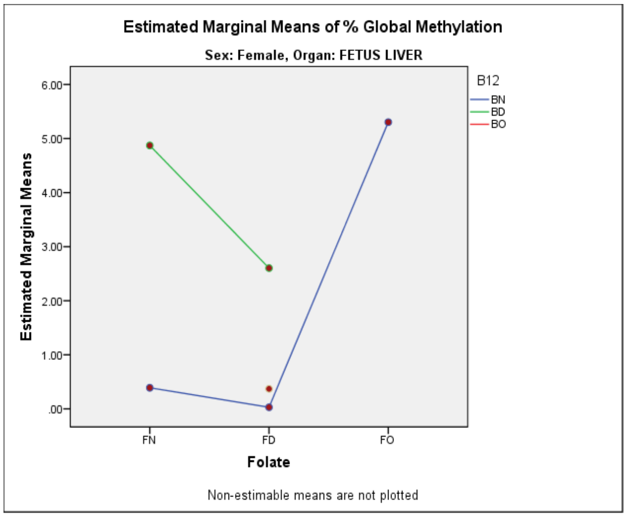

G (ii)

Supplementary figure 11: Profile plots **of global DNA methylation in maternal tissues (A) Brain (F=7.3, df=2) (B) Kidney (F=44.1, df=2) (C) Liver (F=32.5, df=2) (D) Placenta (F=105.0, df=1), fetal tissues (E) (i) Brain (male) (F=7.0, df=1) (ii) Brain (female) (F=2.9, df=1) (F) (i) Kidney (male) (F=0.16, df=1) (ii) Kidney (female) (F=0.19, df=1) (G) (i) Liver (male) (F=65.3, df=1) (ii) Liver (female) (F=17.7,df=1)**

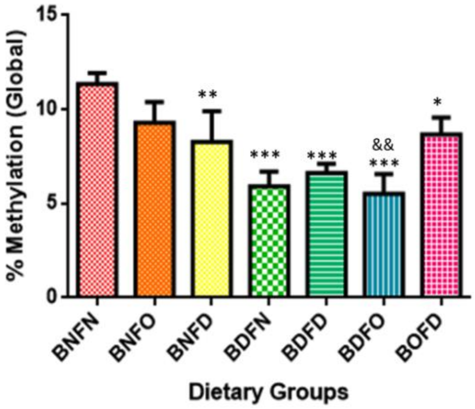

(A)

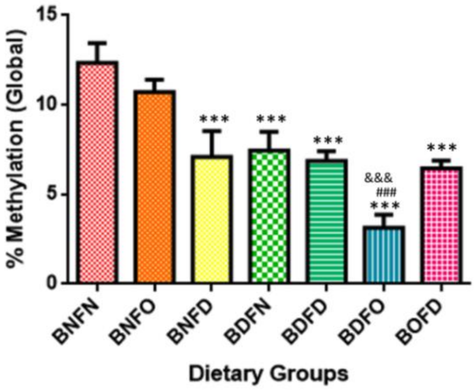

(B)

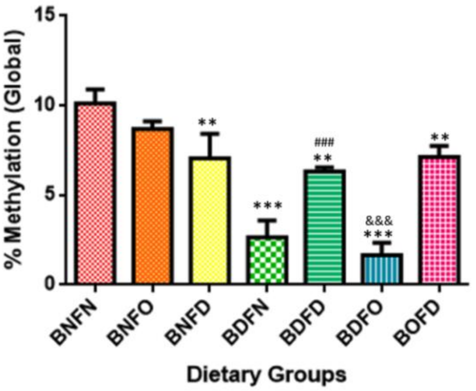

(C)

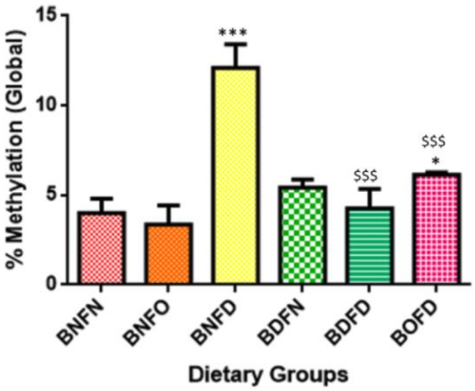

(D)

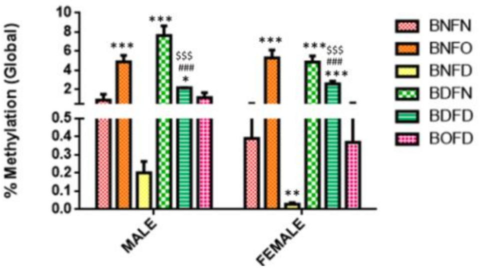

(E)

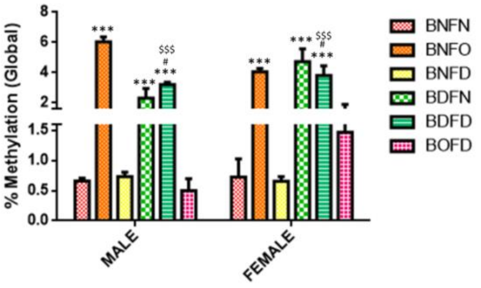

(F)

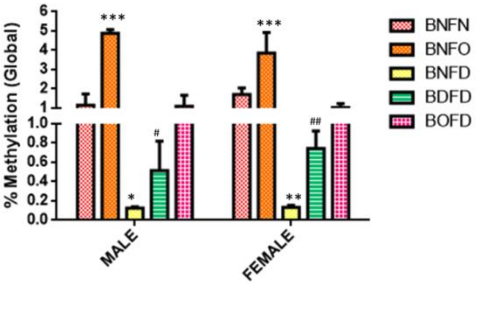

(G)

Supplementary figure 12: Percentage global DNA methylation in maternal tissues (A) Brain (F= 17.22, df=6) **(B) Liver** (F= 42.42, df=6) **(C) Kidney** (F= 58.55, df=6) **(D) Placenta** (F= 50.40, df=5)**, fetal tissues (E) Liver (Male) (F=95.1, df=5), (Female) (F=110.1, df=5) (F) Brain (Male) (F=191.3, df=5), (Female) (F=55.1, df=5) (G) Kidney (Male) (F=83.5, df=5), (Female) (F=23.4, df=5)** *p < 0.05, **p < 0.01, ***p < 0.001 vs BNFN, #p < 0.05, ##p<0.01, ###p < 0.001 vs BDFN, $^{\$}p < 0.05$, $^{\$\$}p<0.01$, $^{\$\$\$}$ p < 0.001 vs BNFD and $^{\&}p < 0.05$, $^{\&\&}p<0.01$, $^{\&\&\&}$ p < 0.001 vs BNFO. The data is presented as mean ± SD. **(N=4)**

B12 normal folate normal (BNFN), B12 normal folate over-supplemented (BNFO), B12 normal folate deficient (BNFD), B12 deficient folate normal (BDFN), B12 deficient folate over-supplemented (BDFO), B12 deficient folate deficient (BDFD), B12 over-supplemented folate normal (BOFN), B12 over-supplemented folate over-supplemented (BOFO), B12 over-supplemented folate deficient (BOFD)

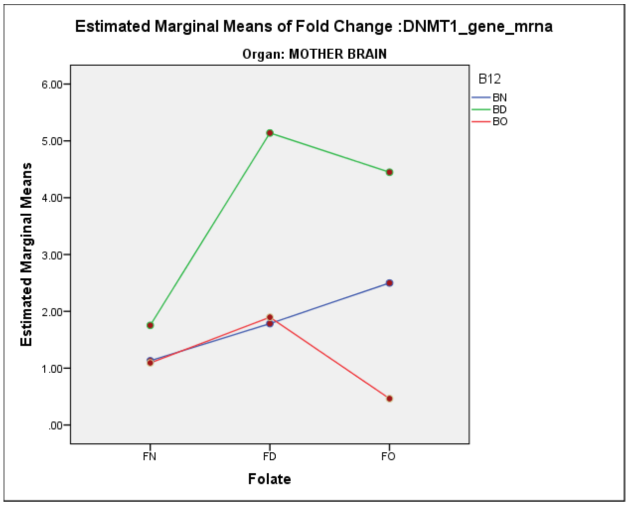
Estimated Marginal Means of Fold Change :DNMT1_gene_mrna
Organ: MOTHER BRAIN
B12
BN
BD
BO
Estimated Marginal Means
6.00
5.00
4.00
3.00
2.00
1.00
.00
FN
FD
FO
Folate

(A)

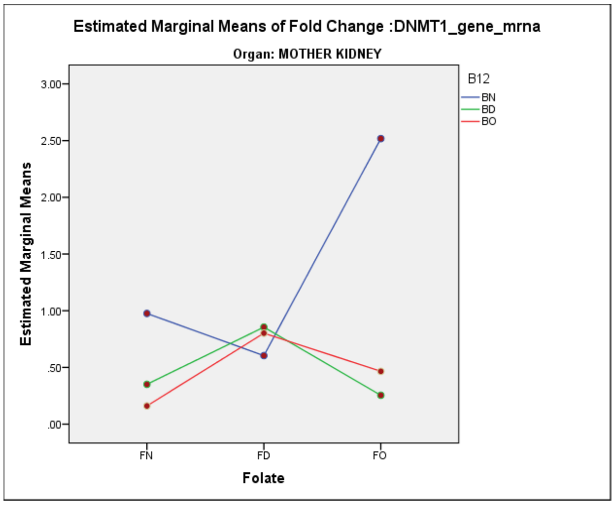
Estimated Marginal Means of Fold Change :DNMT1_gene_mrna
Organ: MOTHER KIDNEY
B12
BN
BD
BO
Estimated Marginal Means
3.00
2.50
2.00
1.50
1.00
.50
.00
FN
FD
FO
Folate

(B)

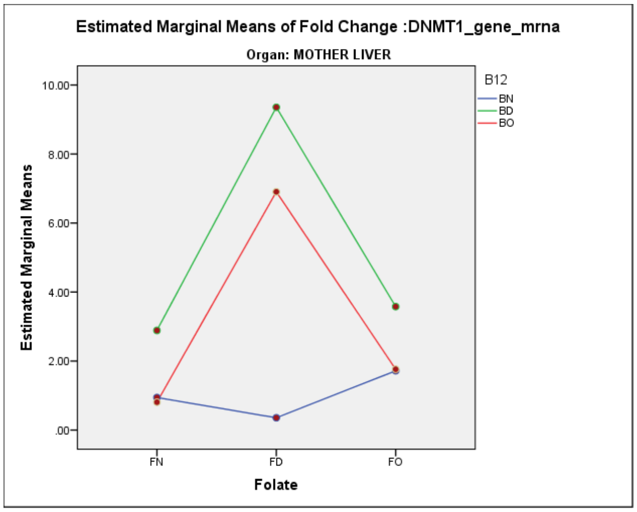
Estimated Marginal Means of Fold Change :DNMT1_gene_mrna
Organ: MOTHER LIVER
B12
BN
BD
BO
Estimated Marginal Means
10.00
8.00
6.00
4.00
2.00
.00
FN
FD
FO
Folate

(C)

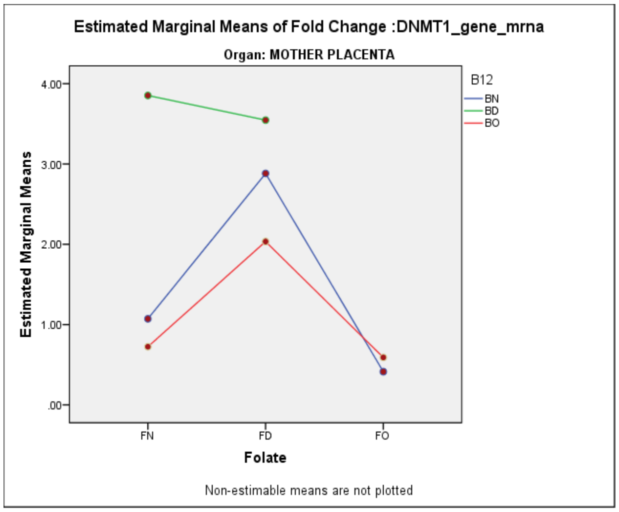
Estimated Marginal Means of Fold Change :DNMT1_gene_mrna
Organ: MOTHER PLACENTA
B12
BN
BD
BO
Estimated Marginal Means
4.00
3.00
2.00
1.00
.00
FN
FD
FO
Folate
Non-estimable means are not plotted

(D)

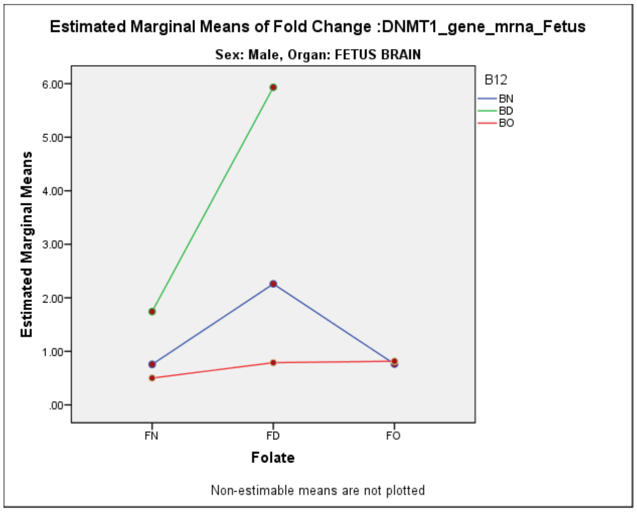
Estimated Marginal Means of Fold Change :DNMT1_gene_mrna_Fetus
Sex: Male, Organ: FETUS BRAIN
B12
BN
BD
BO
Estimated Marginal Means
6.00
5.00
4.00
3.00
2.00
1.00
.00
FN
FD
FO
Folate
Non-estimable means are not plotted

E (i)

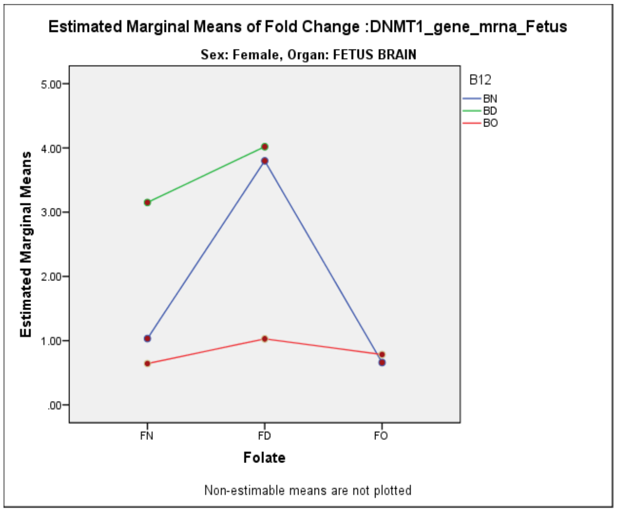
Estimated Marginal Means of Fold Change :DNMT1_gene_mrna_Fetus
Sex: Female, Organ: FETUS BRAIN
B12
BN
BD
BO
Estimated Marginal Means
5.00
4.00
3.00
2.00
1.00
.00
FN
FD
FO
Folate
Non-estimable means are not plotted

E (ii)

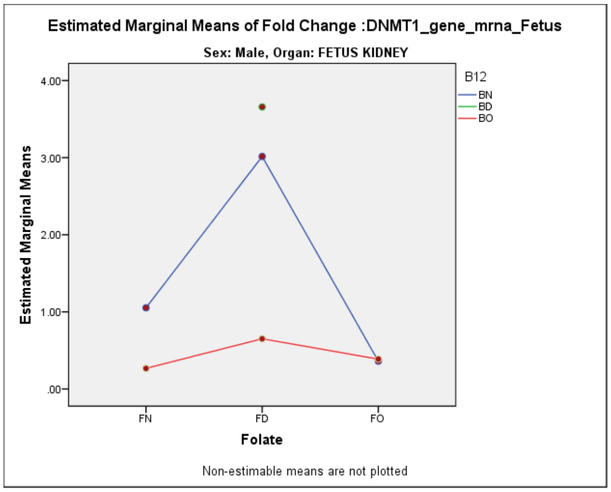

F (i)

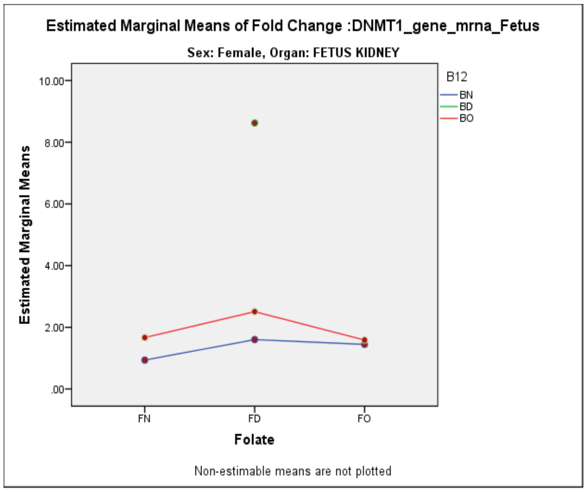

F (ii)

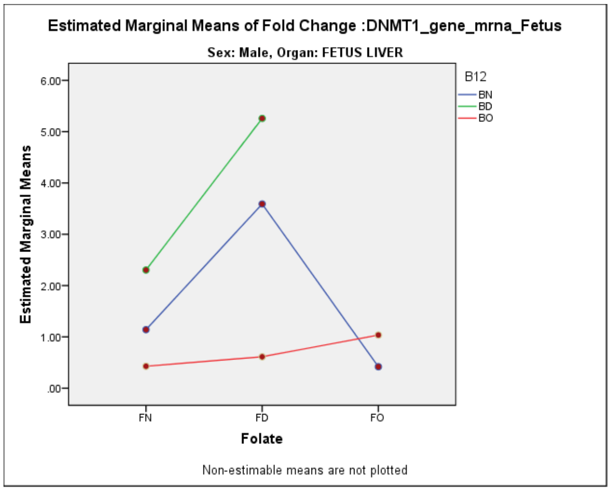

G (i)

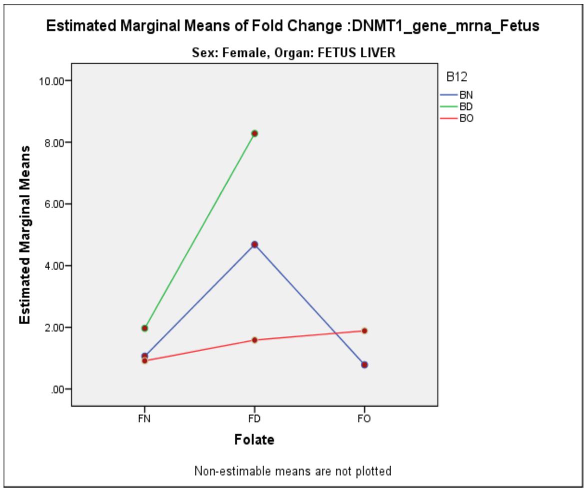

G (ii)

Supplementary figure 13: Profile plots **of mRNA expression of DNMT1 in maternal tissues (A) Brain (F=4.3, df=4) (B) Kidney (F=62.7, df=4) (C) Liver (F=20.7, df=4) (D) Placenta (F=9.3, df=3), fetal tissues (E) (i) Brain (male) (F=13.8, df=3) (ii) Brain (female) (F=6.8, df=3), (F) (i) Kidney (male) (F=6.9, df=2) (ii) Kidney (female) (F=0.19, df=2) (G) (i) Liver (male) (F=9.2, df=3) (ii) Liver (female) (F=17.1,df=3)**

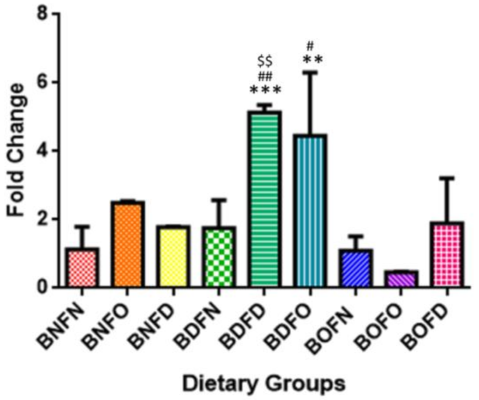

(A)

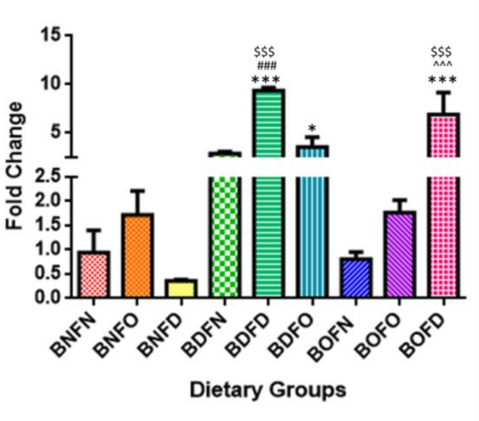

(B)

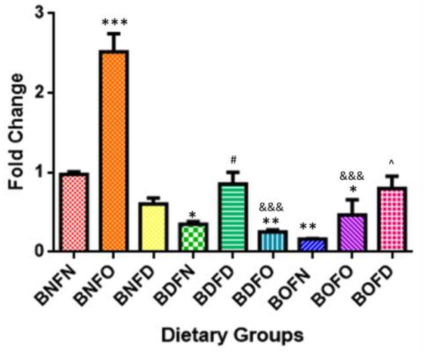

(C)

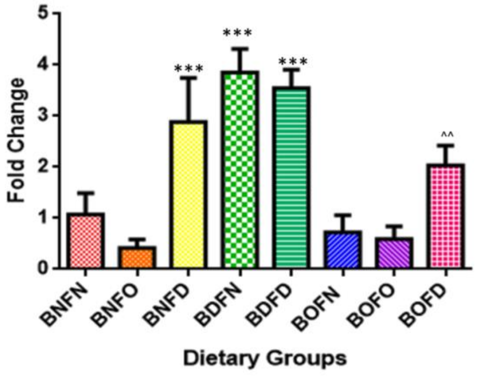

(D)

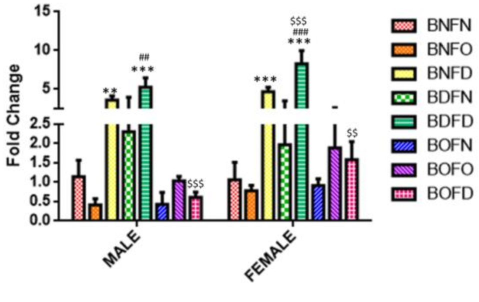

(E)

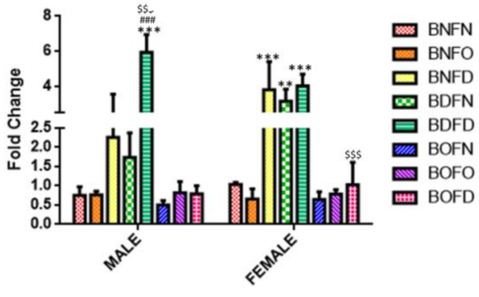

(F)

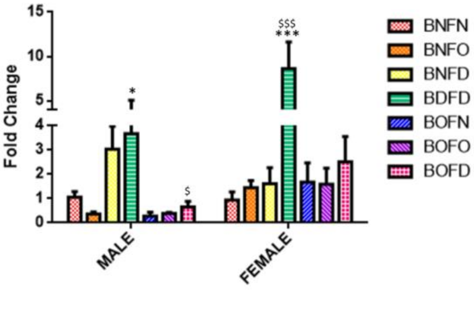

(G)

Supplementary figure 14: Fold change in mRNA of DNMT1 gene in maternal tissues (A) Brain (F= 10.16, df=8) **(B) Liver** (F= 37.14, df= 8) **(C) Kidney** (F= 64.60, df=8) **(D) Placenta** (F= 38.09, df=7)**, fetal tissues (E) Liver (Male) (F=20.4,df=7), (Female) (F=33.1, df=7) Brain (Male) (F=32.1,df=7), (Female) (F=17.9, df=7) (G) Kidney (Male) (F=18.5,df=6), (Female) (F=17.2, df=6) normalized with GAPDH.** *p < 0.05, **p < 0.01, ***p < 0.001 vs BNFN, #p < 0.05, ##p<0.01, ###p < 0.001 vs BDFN, ^p < 0.05, ^^p<0.01, ^^^p < 0.001 vs BOFN, $^{\$}$p < 0.05, $^{\$\$}$p<0.01, $^{\$\$\$}$ p < 0.001 vs BNFD and $^{\&}$p < 0.05, $^{\&\&}$p<0.01, $^{\&\&\&}$ p < 0.001 vs BNFO. The data is presented as mean ± SD. **(N=4)**

B12 normal folate normal (BNFN), B12 normal folate over-supplemented (BNFO), B12 normal folate deficient (BNFD), B12 deficient folate normal (BDFN), B12 deficient folate over-supplemented (BDFO), B12 deficient folate deficient (BDFD), B12 over-supplemented folate normal (BOFN), B12 over-supplemented folate over-supplemented (BOFO), B12 over-supplemented folate deficient (BOFD)

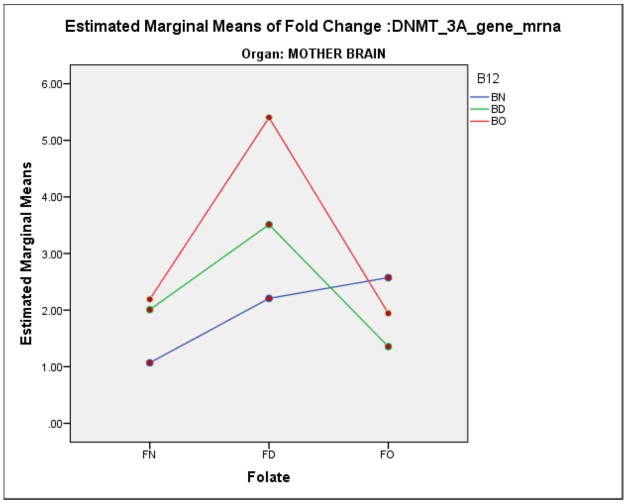
Estimated Marginal Means of Fold Change :DNMT_3A_gene_mrna
Organ: MOTHER BRAIN
Estimated Marginal Means
6.00
5.00
4.00
3.00
2.00
1.00
.00
FN
FD
FO
Folate
B12
BN
BD
BO

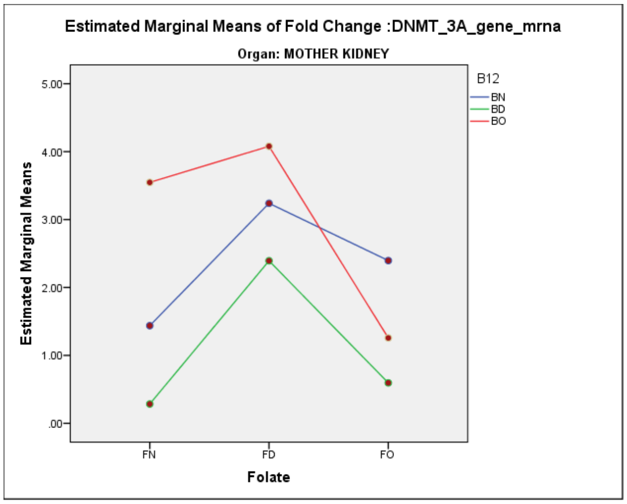
Estimated Marginal Means of Fold Change :DNMT_3A_gene_mrna
Organ: MOTHER KIDNEY
Estimated Marginal Means
5.00
4.00
3.00
2.00
1.00
.00
FN
FD
FO
Folate
B12
BN
BD
BO

(A)

(B)

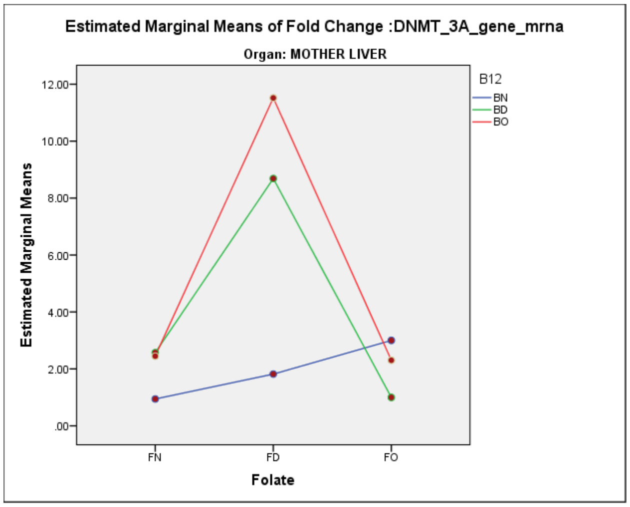
Estimated Marginal Means of Fold Change :DNMT_3A_gene_mrna
Organ: MOTHER LIVER
Estimated Marginal Means
12.00
10.00
8.00
6.00
4.00
2.00
.00
FN
FD
FO
Folate
B12
BN
BD
BO

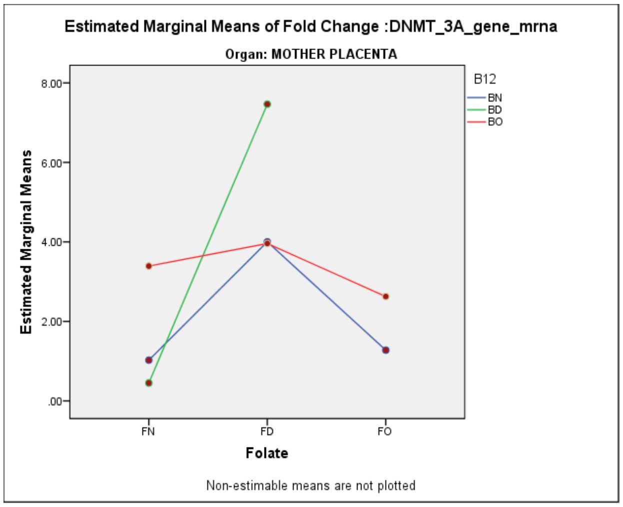
Estimated Marginal Means of Fold Change :DNMT_3A_gene_mrna
Organ: MOTHER PLACENTA
Estimated Marginal Means
8.00
6.00
4.00
2.00
.00
FN
FD
FO
Folate
Non-estimable means are not plotted
B12
BN
BD
BO

(C)

(D)

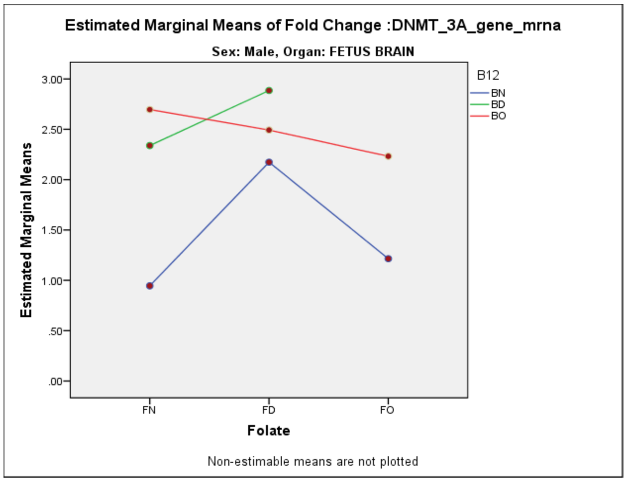
Estimated Marginal Means of Fold Change :DNMT_3A_gene_mrna
Sex: Male, Organ: FETUS BRAIN
Estimated Marginal Means
3.00
2.50
2.00
1.50
1.00
.50
.00
FN
FD
FO
Folate
Non-estimable means are not plotted
B12
BN
BD
BO

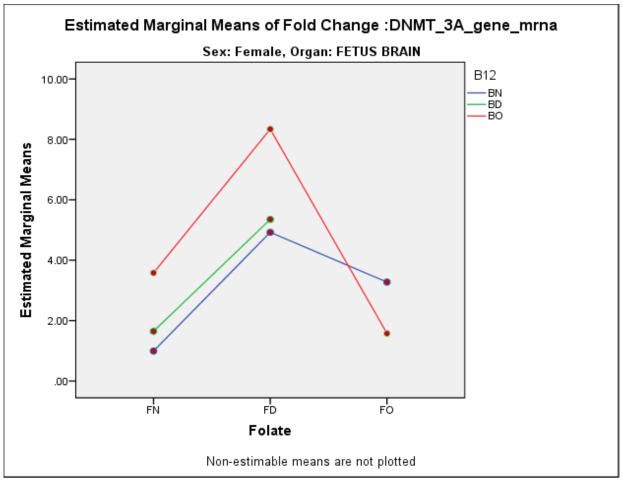
Estimated Marginal Means of Fold Change :DNMT_3A_gene_mrna
Sex: Female, Organ: FETUS BRAIN
Estimated Marginal Means
10.00
8.00
6.00
4.00
2.00
.00
FN
FD
FO
Folate
Non-estimable means are not plotted
B12
BN
BD
BO

E (i)

E (ii)

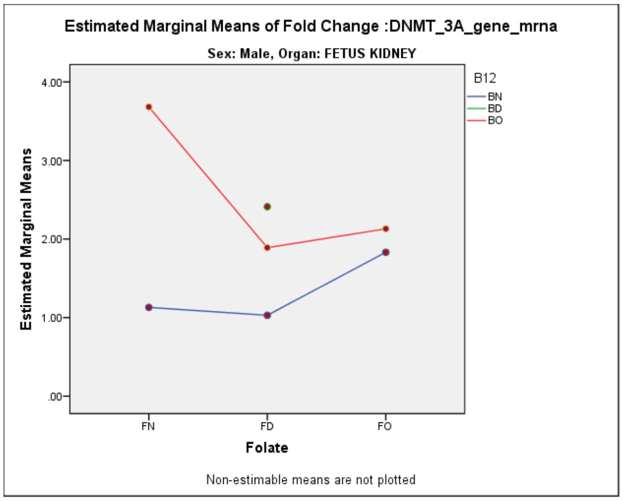

F (i)

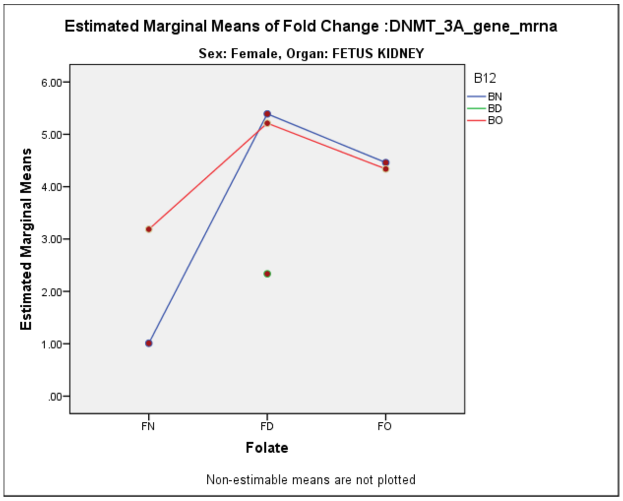

F (ii)

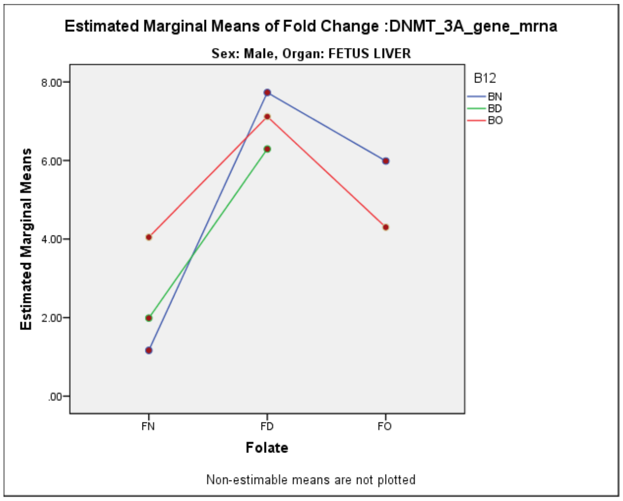

G (i)

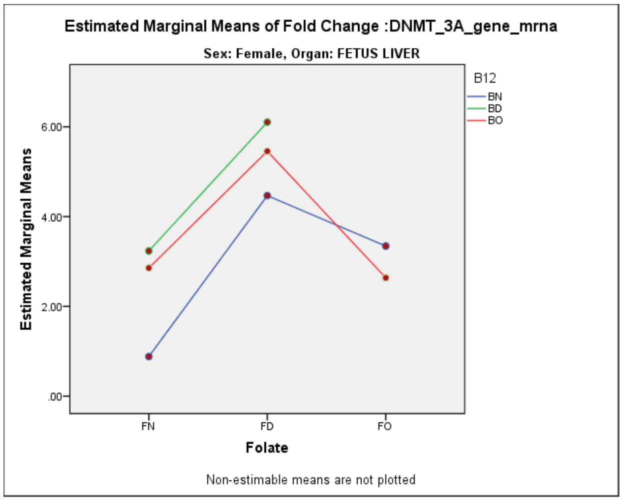

G (ii)

Supplementary figure 15: Profile plots **of mRNA expression of DNMT3A in maternal tissues (A) Brain (F=5.1, df=4) (B) Kidney (F=7.2, df=4) (C) Liver (F=54.7, df=4) (D) Placenta (F=16.4, df=3), fetal tissues (E) (i) Brain (male) (F=1.0, df=3) (ii) Brain (female) (F=6.9, df=3) (F) (i) Kidney (male) (F=5.3, df=2) (ii) Kidney (female) (F=2.18, df=2) (G) (i) Liver (male) (F=4.8, df=3) (ii) Liver (female) (F=1.86,df=3)**

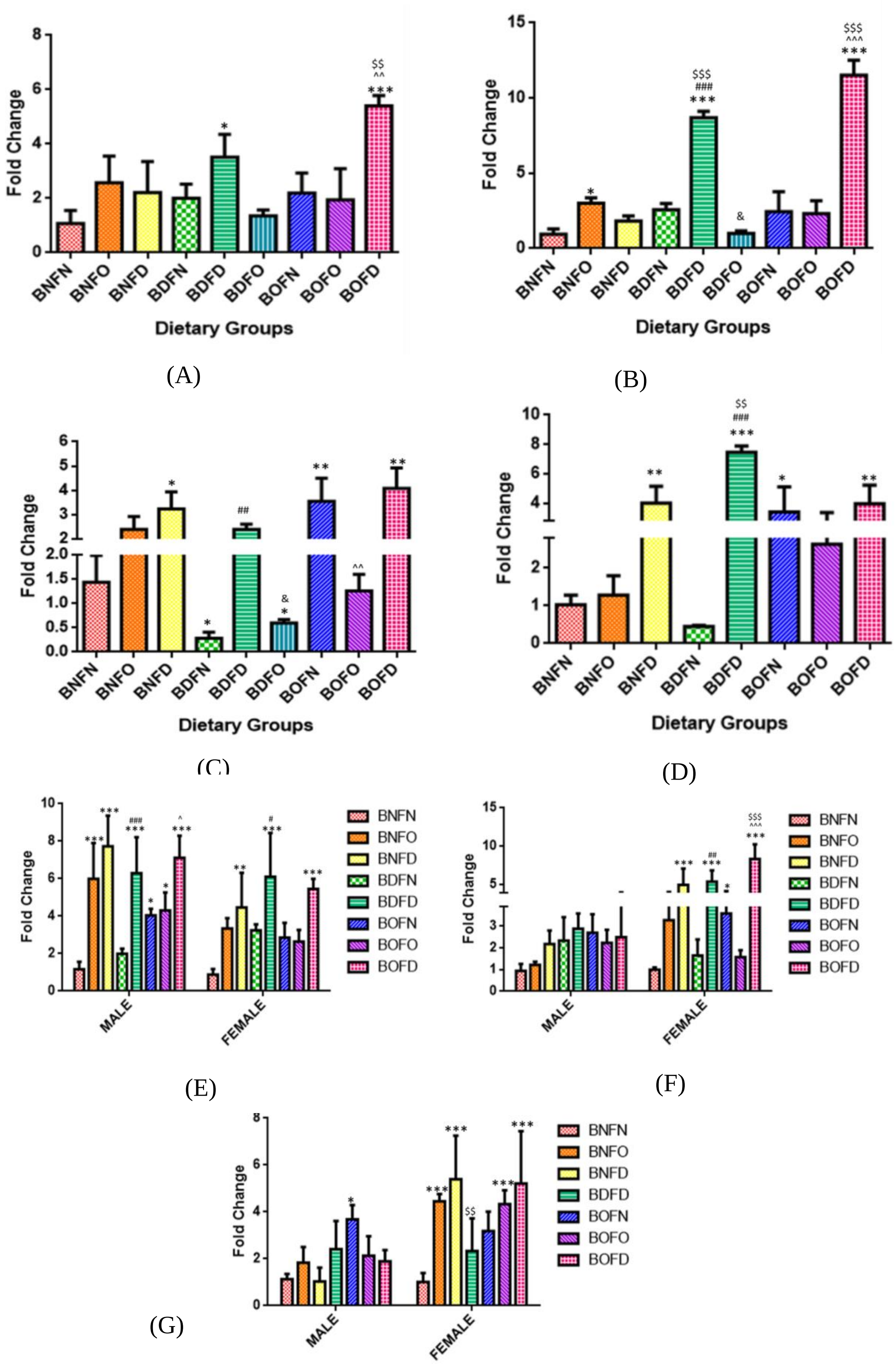

Supplementary figure 16: Fold change in mRNA of DNMT3A gene in maternal tissues (A) Brain (F= 8.21, df=8) **(B) Liver** (F= 86.47, df=8) **(C) Kidney** (F= 16.53, df=8) **(D) Placenta** (F= 23.55, df=7)**, fetal tissues (E) Liver (Male) (F=14.1, df=7), (Female) (F=8.39, df=7) (F) Brain (Male) (F=2.88, df=7), (Female) (F=16.3, df=7) (G) Kidney (Male) (F=6.17, df=6), (Female) (F=6.23, df=6) normalized with GAPDH.** *$p < 0.05$, **$p < 0.01$, ***$p < 0.001$ vs BNFN, #$p < 0.05$, ##$p<0.01$, ###$p < 0.001$ vs BDFN, ^$p < 0.05$, ^^$p<0.01$, ^^^$p < 0.001$ vs BOFN, $^{\$}p < 0.05$, $^{\$\$}p<0.01$, $^{\$\$\$}p < 0.001$ vs BNFD and $^{\&}p < 0.05$, $^{\&\&}p<0.01$, $^{\&\&\&}p < 0.001$ vs BNFO. **(N=4)**

The data is presented as mean ± SD. B12 normal folate normal (BNFN), B12 normal folate over-supplemented (BNFO), B12 normal folate deficient (BNFD), B12 deficient folate normal (BDFN), B12 deficient folate over-supplemented (BDFO), B12 deficient folate deficient (BDFD), B12 over-supplemented folate normal (BOFN), B12 over-supplemented folate over-supplemented (BOFO), B12 over-supplemented folate deficient (BOFD)

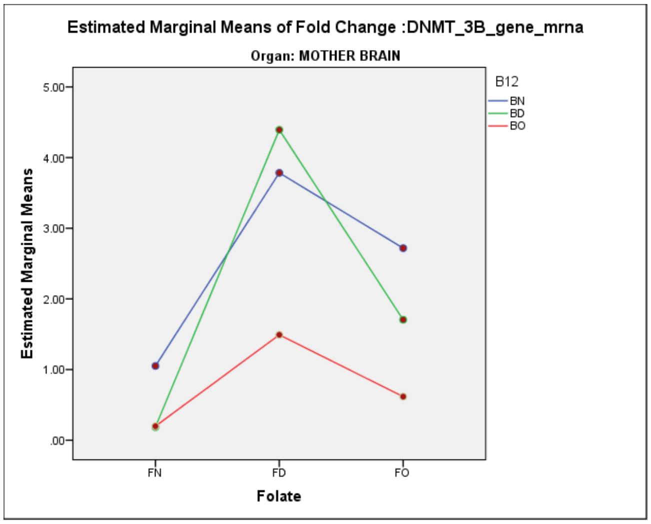
Estimated Marginal Means of Fold Change :DNMT_3B_gene_mrna
Organ: MOTHER BRAIN
B12
BN
BD
BO
Estimated Marginal Means
5.00
4.00
3.00
2.00
1.00
.00
FN
FD
FO
Folate

(A)

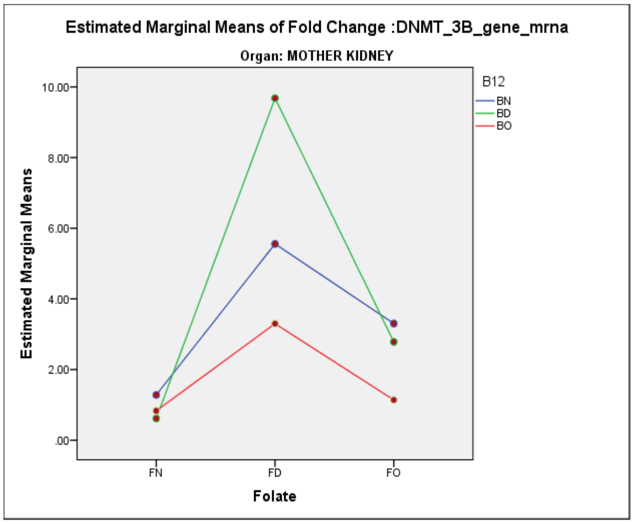
Estimated Marginal Means of Fold Change :DNMT_3B_gene_mrna
Organ: MOTHER KIDNEY
B12
BN
BD
BO
Estimated Marginal Means
10.00
8.00
6.00
4.00
2.00
.00
FN
FD
FO
Folate

(B)

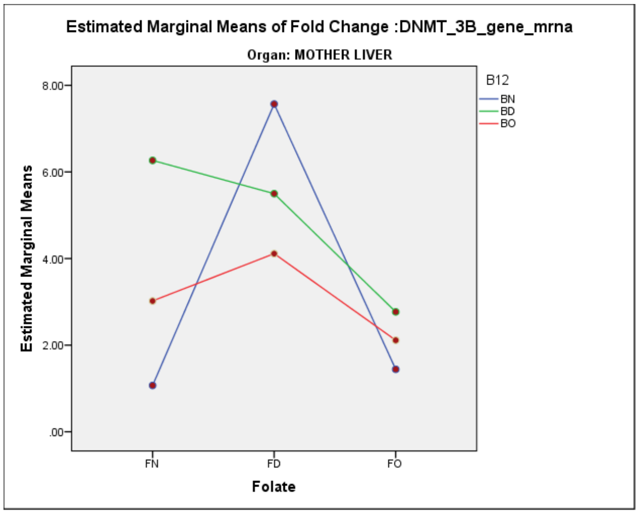
Estimated Marginal Means of Fold Change :DNMT_3B_gene_mrna
Organ: MOTHER LIVER
B12
BN
BD
BO
Estimated Marginal Means
8.00
6.00
4.00
2.00
.00
FN
FD
FO
Folate

(C)

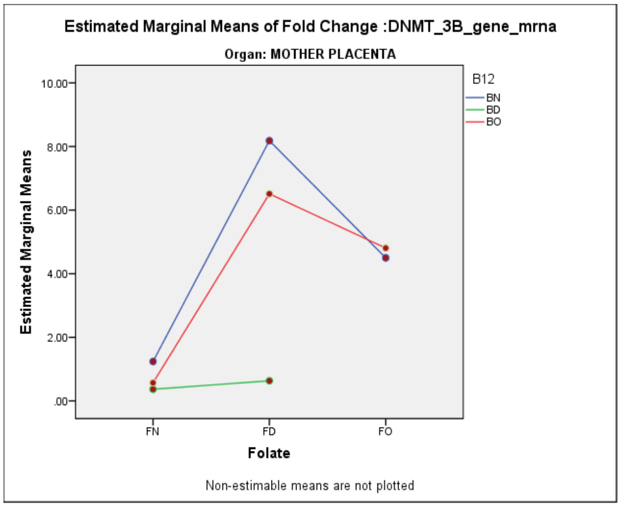
Estimated Marginal Means of Fold Change :DNMT_3B_gene_mrna
Organ: MOTHER PLACENTA
B12
BN
BD
BO
Estimated Marginal Means
10.00
8.00
6.00
4.00
2.00
.00
FN
FD
FO
Folate
Non-estimable means are not plotted

(D)

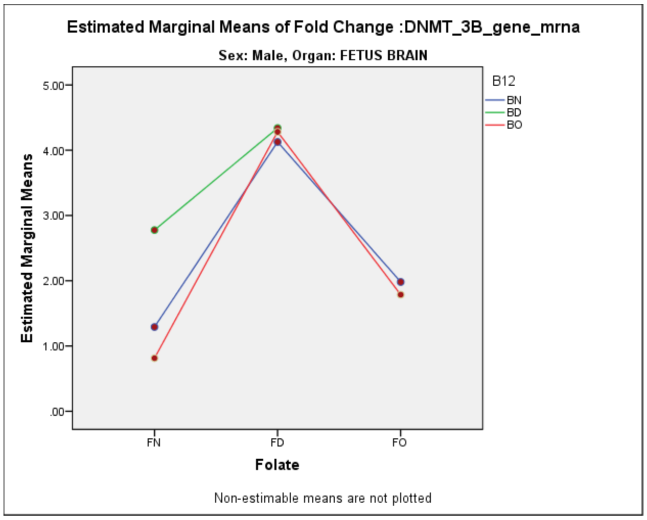
Estimated Marginal Means of Fold Change :DNMT_3B_gene_mrna
Sex: Male, Organ: FETUS BRAIN
B12
BN
BD
BO
Estimated Marginal Means
5.00
4.00
3.00
2.00
1.00
.00
FN
FD
FO
Folate
Non-estimable means are not plotted

E (i)

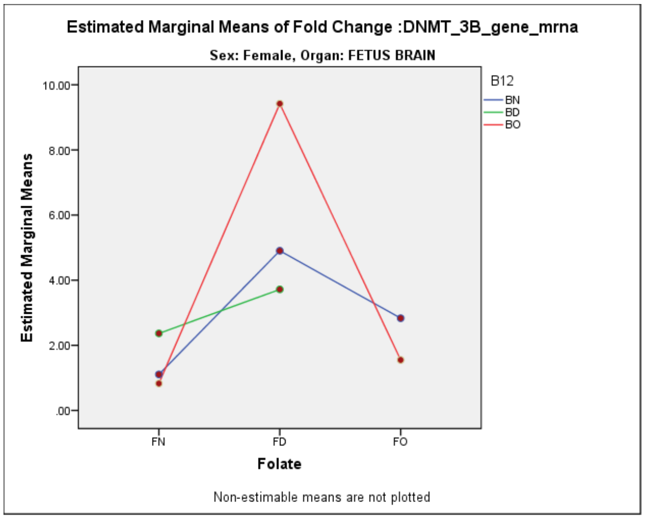
Estimated Marginal Means of Fold Change :DNMT_3B_gene_mrna
Sex: Female, Organ: FETUS BRAIN
B12
BN
BD
BO
Estimated Marginal Means
10.00
8.00
6.00
4.00
2.00
.00
FN
FD
FO
Folate
Non-estimable means are not plotted

E (ii)

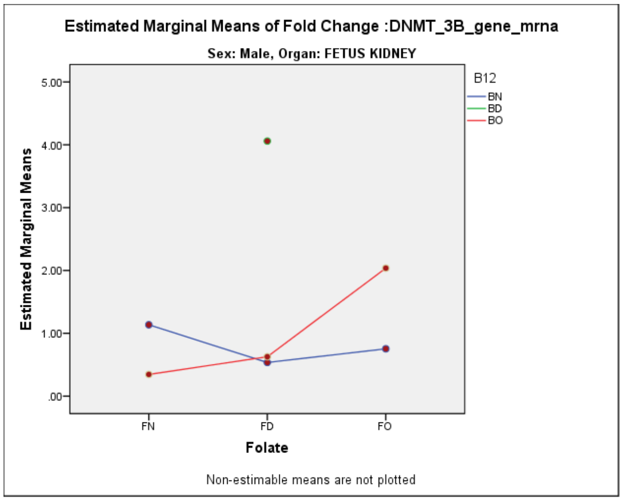

F (i)

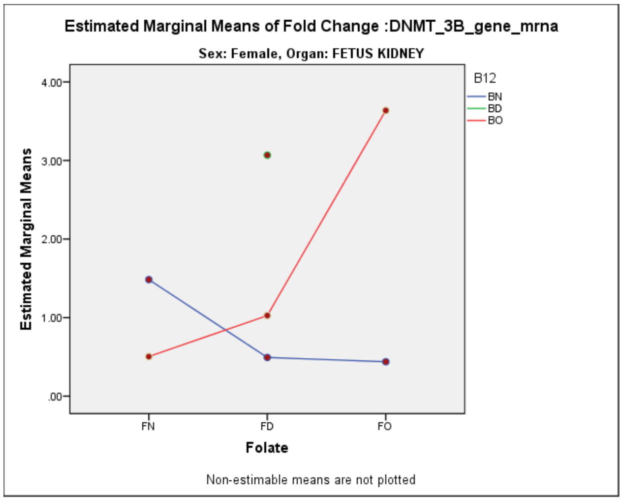

F (ii)

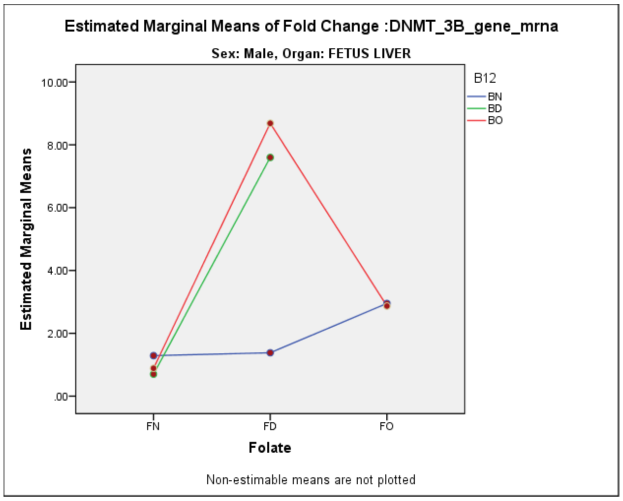

G (i)

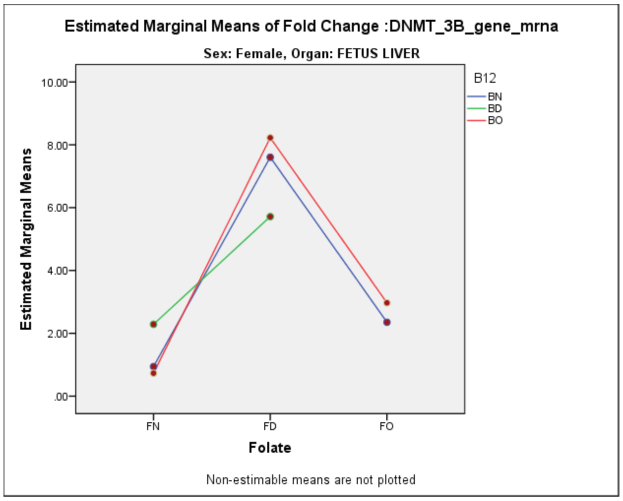

G (ii)

Supplementary figure 17: Profile plots **of mRNA expression of DNMT3B in maternal tissues (A) Brain (F=4.4, df=4) (B) Kidney (F=44.0, df=4) (C) Liver (F=13.9, df=4) (D) Placenta (F=24.9, df=3), fetal tissues (E) (i) Brain (male) (F=1.6, df=3) (ii) Brain (female) (F=28.1, df=3) (F) (i) Kidney (male) (F=8.3, df=2) (ii) Kidney (female) (F=32.7, df=2) (G) (i) Liver (male) (F=45.7, df=3) (ii) Liver (female) (F=11.5,df=3)**

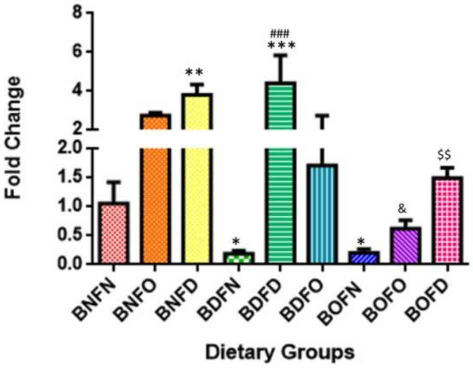

(A)

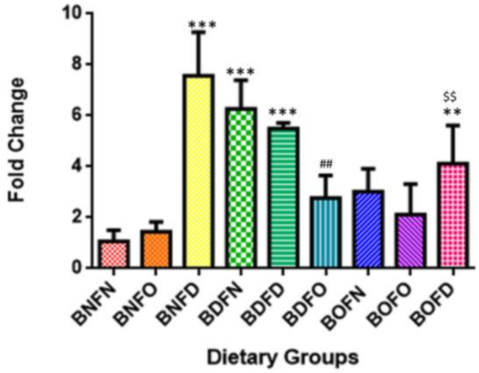

(B)

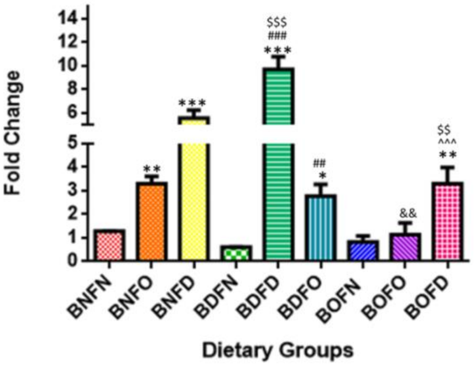

(C)

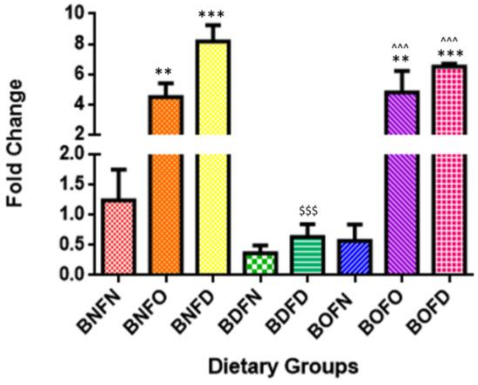

(D)

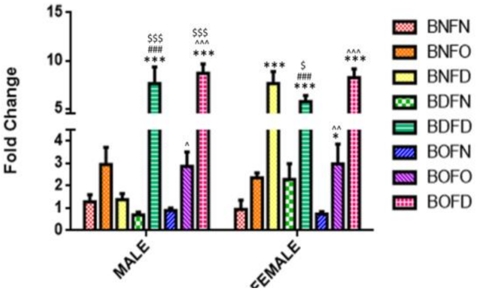

(E)

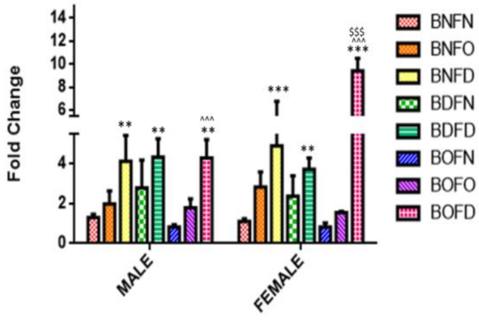

(F)

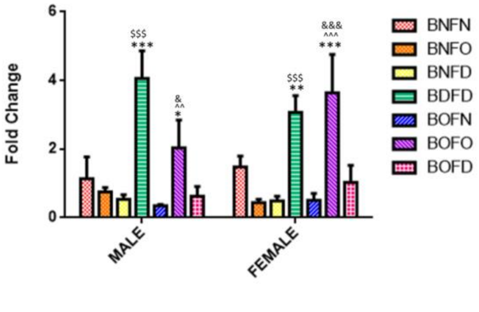

(G)

Supplementary figure 18: Fold change in mRNA of DNMT3B gene in maternal tissues (A) Brain (F= 17.61, df=8) **(B) Liver** (F= 18.61, df=8) **(C) Kidney** (F= 112.1, df=8) **(D) Placenta** (F= 50.89, df=7)**, fetal tissues (E) Liver (Male) (F=60.9, df=7), (Female) (F=63.8, df=7) (F) Brain (Male) (F=10.7, df=7), (Female) (F=38.1, df=7) (G) Kidney (Male) (F=26.6, df=6), (Female) (F=25.3, df=6) normalized with GAPDH.** *p < 0.05, **p < 0.01, ***p < 0.001 vs BNFN, #p < 0.05, ##p<0.01, ###p < 0.001 vs BDFN, ^p < 0.05, ^^p<0.01, ^^^p < 0.001 vs BOFN, $^{\$}$p < 0.05, $^{\$\$}$p<0.01, $^{\$\$\$}$ p < 0.001 vs BNFD and $^{\&}$p < 0.05, $^{\&\&}$p<0.01, $^{\&\&\&}$ p < 0.001 vs BNFO. The data is presented as mean ± SD. **(N=4)**

B12 normal folate normal (BNFN), B12 normal folate over-supplemented (BNFO), B12 normal folate deficient (BNFD), B12 deficient folate normal (BDFN), B12 deficient folate over-supplemented (BDFO), B12 deficient folate deficient (BDFD), B12 over-supplemented folate normal (BOFN), B12 over-supplemented folate over-supplemented (BOFO), B12 over-supplemented folate deficient (BOFD)

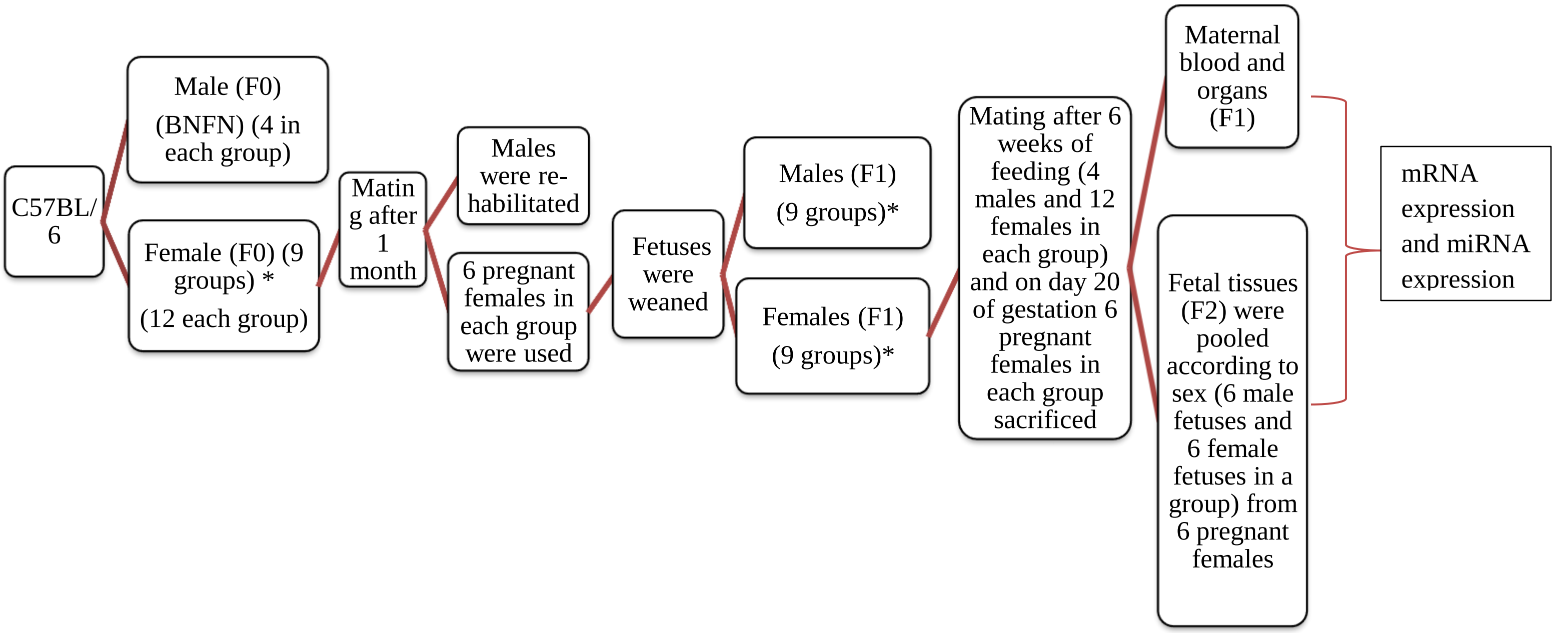

*B12 normal folate normal (BNFN), B12 normal folate over-supplemented (BNFO), B12 normal folate deficient (BNFD), B12 deficient folate normal (BDFN), B12 deficient folate over-supplemented (BDFO), B12 deficient folate deficient (BDFD), B12 over-supplemented folate normal (BOFN), B12 over-supplemented folate over-supplemented (BOFO), B12 over-supplemented folate deficient (BOFD)

Supplementary figure 19: Work flow of the study

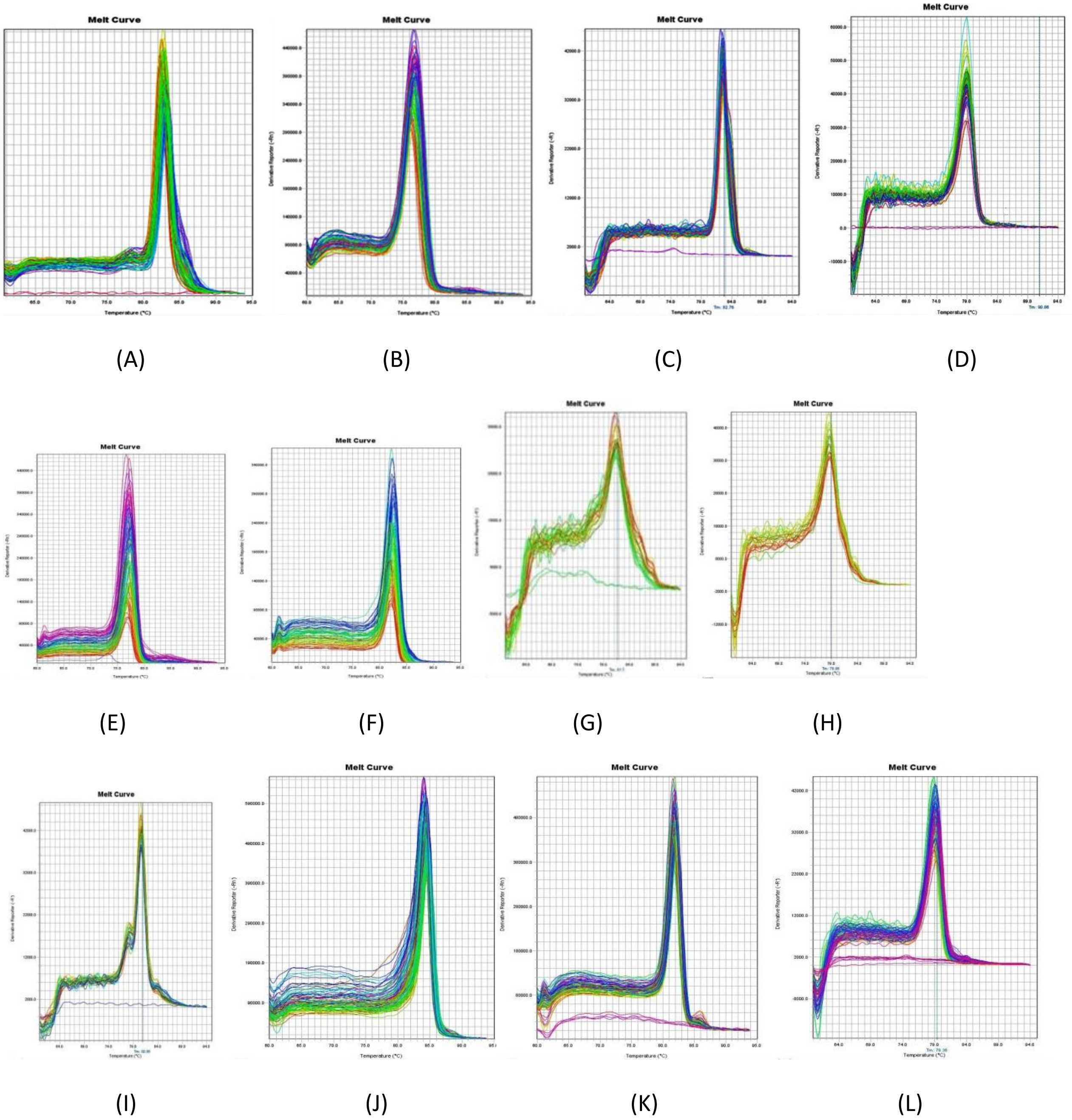

Supplementary figure 20: Melting profile of genes under study A) RFC, B) PCFT, C) FOLR1, D) GAPDH E) LMBRD1 F) TC-II G) miRNA-483, H) miRNA-221 I) miRNA-133 J) DNMT1 K) DNMT3A L) DNMT3B

Supplementary table 1: Primer sequences for SRY and MYOGENIN

Gene	Primer sequence	Length
**SRY**	**F:** 5'-TCATGAGACTGCCAACCACAG- 3'<br>**R:** 5'-CATGACCACCACCACCACCAA-3'	**441bp**
**MYOGENIN**	**F:** 5'-CGTGGGCATGTAAGGTGTGTA-3'<br>**R:** 5'-CCTGCGCTTCTCCCTCAGT-3'	**79bp**

Supplementary table 2: mRNA primer sequence

Gene	Primers sequence	Annealing Temp. (^{0}C)	Product
GAPDH	F: AGCTTGTCATCAACGGGAAG<br>R: TTTGATGTTAGTGGGGTCTCG	62^{0}C	61 bp
RFC	F- GGGTGTGCTACGTGACCTTT<br>R- ACGGAACTGATCACGGACTT	58^{0}C	211bp
PCFT	F- GAATGGTGGTCTTTGCGTTT<br>R- TCCGTACCCTGTGAACATGA	62^{0}C	55bp
FOLR1	F- GGCCCTGAGGACAATTTACA<br>R- TCGGGGAACACTCATAGAGG	61^{0}C	194bp
LMBRD1	F-AGCAGGAATAGATTCGGGTTTCA<br>R-TTTGTAACAACGGCAAAAGCA	60^{0}C	79bp
TC-II	F-AGGCCCCTGAGGGCTACTT<br>R-CCCGGATGCAGGAGATGTC	60^{0}C	79bp
DNMT1	F-CGGTCATTCCAGATGATTCCTC<br>R-TGCTGTGGATGTAGGAAAGCTG	60^{0}C	200bp
DNMT3A	F-TCCCGGGGCCGACTGCGA<br>R-TCCCCCACACCAGCTCTC	62^{0}C	400bp
DNMT3B	F-GAACATGCGCCTGCAAGA<br>R-GCACAGACTTCGGAGGCAAT	62^{0}C	204bp

Amp.Length bp=Amplicon length in base pairs.

Supplementary Table 3: miRNA primer sequence

micro RNA	**Sequence**
miR-483	ACGGGAGAAGAGAAGGGAGTGGTTTTTGGGTGCCTCACTCCTC
miR-221	ACCTGGCATACAATGTAGATTTCTGT
miR-133	GCTGGTCAAACGGAACCAAGTC
